# Solid-state mechanochemical cross-coupling of insoluble substrates into insoluble products by removable solubilizing silyl groups: uniform synthesis of nonsubstituted linear oligothiophenes†

**DOI:** 10.1039/d3ra05571j

**Published:** 2023-09-29

**Authors:** Koji Kubota, Keisuke Kondo, Tamae Seo, Mingoo Jin, Hajime Ito

**Affiliations:** a Division of Applied Chemistry, Graduate School of Engineering, Hokkaido University Sapporo Hokkaido Japan kbt@eng.hokudai.ac.jp hajito@eng.hokudai.ac.jp; b Institute for Chemical Reaction Design and Discovery (WPI-ICReDD), Hokkaido University Sapporo Hokkaido Japan

## Abstract

Conventional solution-based organic reactions that involve insoluble substrates are challenging and inefficient. Furthermore, even if the reaction is successful, the corresponding products are insoluble in most cases, making their isolation and subsequent transformations difficult. Hence, the conversion of insoluble compounds into insoluble products remains a challenge in practical synthetic chemistry. In this study, we showcase a potential solution to address these solubility issues by combining a mechanochemical cross-coupling approach with removable solubilizing silyl groups. Our strategy involves solid-state Suzuki–Miyaura cross-coupling reactions between organoboron nucleophiles bearing a silyl group with long alkyl chains and insoluble polyaromatic halides. The silyl group on the nucleophile can act as a solubilizing group that enables product isolation *via* silica gel column chromatography and can be easily removed by the addition of fluoride anions to form the desired insoluble coupling products with sufficient purity. Furthermore, we demonstrate that after aromatic electrophilic bromination of the desilylated products, sequential solid-state cross-coupling of the obtained insoluble brominated substrates, followed by desilylation, afforded further π-extended functional molecules. Using this conceptually new protocol, we achieved the first uniform synthesis of the longest nonsubstituted linear insoluble 9-*mer* oligothiophene.

## Abstract 2

We developed a new solid-state mechanochemical strategy using a removable and solubilizing silyl group that enables the organic transformation of insoluble substrates into insoluble products.

## Graphical abstract

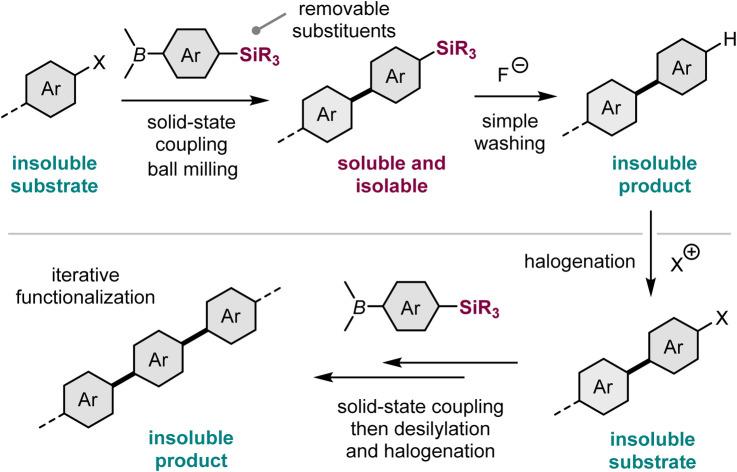

## Introduction

**Scheme 1:**
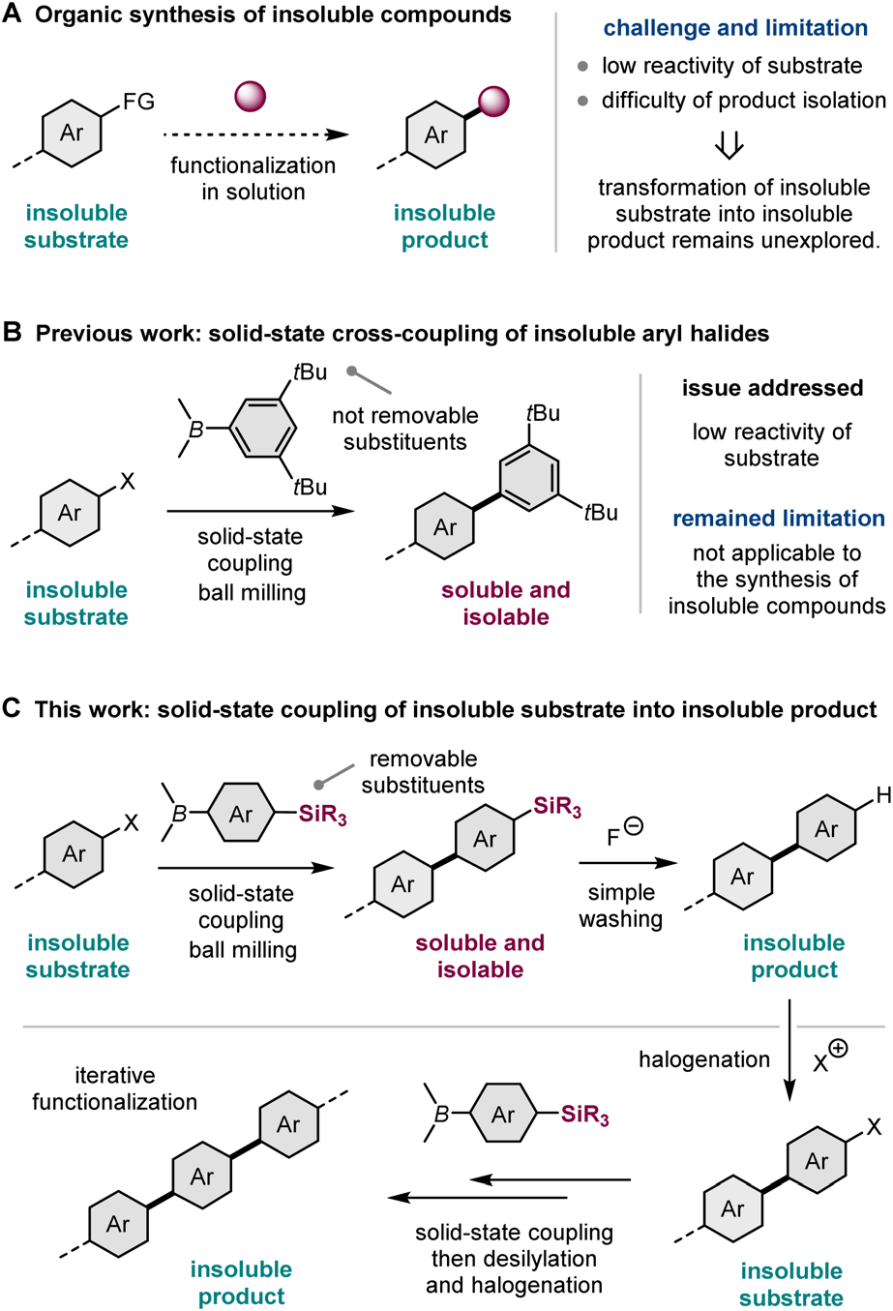
Strategy for the solid-state cross-coupling of insoluble substrates into insoluble products.

Solution-based organic synthesis generally uses organic solvents to dissolve reactants in a reaction flask. Accordingly, reactions of insoluble substrates, such as large polyaromatic molecules, often require a large amount of organic solvent, resulting in a significant decrease in the reaction rate (Scheme 1A).^1^ Therefore, it is often difficult to obtain synthetically acceptable yields even if such reactions are carried out using a slurry at an elevated temperature with a long reaction time. Furthermore, the corresponding products are also likely insoluble in most cases, making isolation and subsequent molecular transformations difficult (Scheme 1A).^1^ Therefore, the conversion of insoluble compounds into insoluble products represents one of the unsolved challenges of practical organic synthesis. The development of a new synthetic strategy to overcome these solubility issues would greatly expand the scope of synthesizable organic molecules.

Recently, mechanochemical synthesis using ball milling has emerged as a new approach for carrying out organic transformations under solvent-less solid-state conditions.^2^ We have previously reported fast and efficient solid-state Suzuki–Miyaura cross-coupling reactions that use a high-temperature ball-milling technique and are facilitated by a catalytic system consisting of Pd(OAc)_2_/SPhos/1,5-cyclooctadiene (1,5-cod) (Scheme 1B).^3–8^ Notably, this solid-state approach is applicable to the reactions of insoluble aryl halides that are unreactive under solution-based conditions.^3*a*^ The key to achieving this solid-state cross-coupling of insoluble compounds is not only the use of strong mechanical agitation achieved *via* ball milling at high temperature^9^ but also the addition of small amounts of 1,5-cod, which act as catalyst stabilizers to facilitate Suzuki–Miyaura coupling under solid-state reaction conditions.^3^ This mechanochemical approach provides a practical solution for the aforementioned solubility issue of starting materials. However, problems related to the insolubility of the reaction products remain unresolved. In our previous study, we used arylboron nucleophiles with bulky alkyl substituents, such as the *t*Bu group, to solubilize the coupling products in an organic solvent, which enabled their isolation *via* silica gel column chromatography (Scheme 1B).^3*a*^ When substrates without such solubilizing substituents were used, the corresponding products could not be isolated because of their significantly low solubility in organic solvents.^3*a*^

In this study, to address the solubility issue of the reaction products, we focused on the use of a removable solubilizing group (Scheme 1C).^10^ Our synthetic approach involves the solid-state Suzuki–Miyaura cross-coupling of organoboron nucleophiles bearing a silyl group with long alkyl chains, which can act as a solubilizing group, enabling product isolation *via* silica gel column chromatography or recrystallization. The remaining silyl groups were easily removed by the addition of fluoride anions, followed by simple washing to form the desired insoluble coupling products with sufficient purity. This two-step synthetic strategy expands the scope of synthesizable organic molecules *via* reactions involving insoluble substrates and insoluble products. Furthermore, we envisioned that if the aromatic electrophilic halogenation of the desilylated products was feasible, sequential solid-state cross-coupling of insoluble halogenated substrates followed by desilylation would afford further π-extended functional molecules (Scheme 1C). This iterative solid-state synthesis enables multistep molecular transformations of insoluble organic compounds that cannot be carried out using solution-based approaches.

**Scheme 2:**
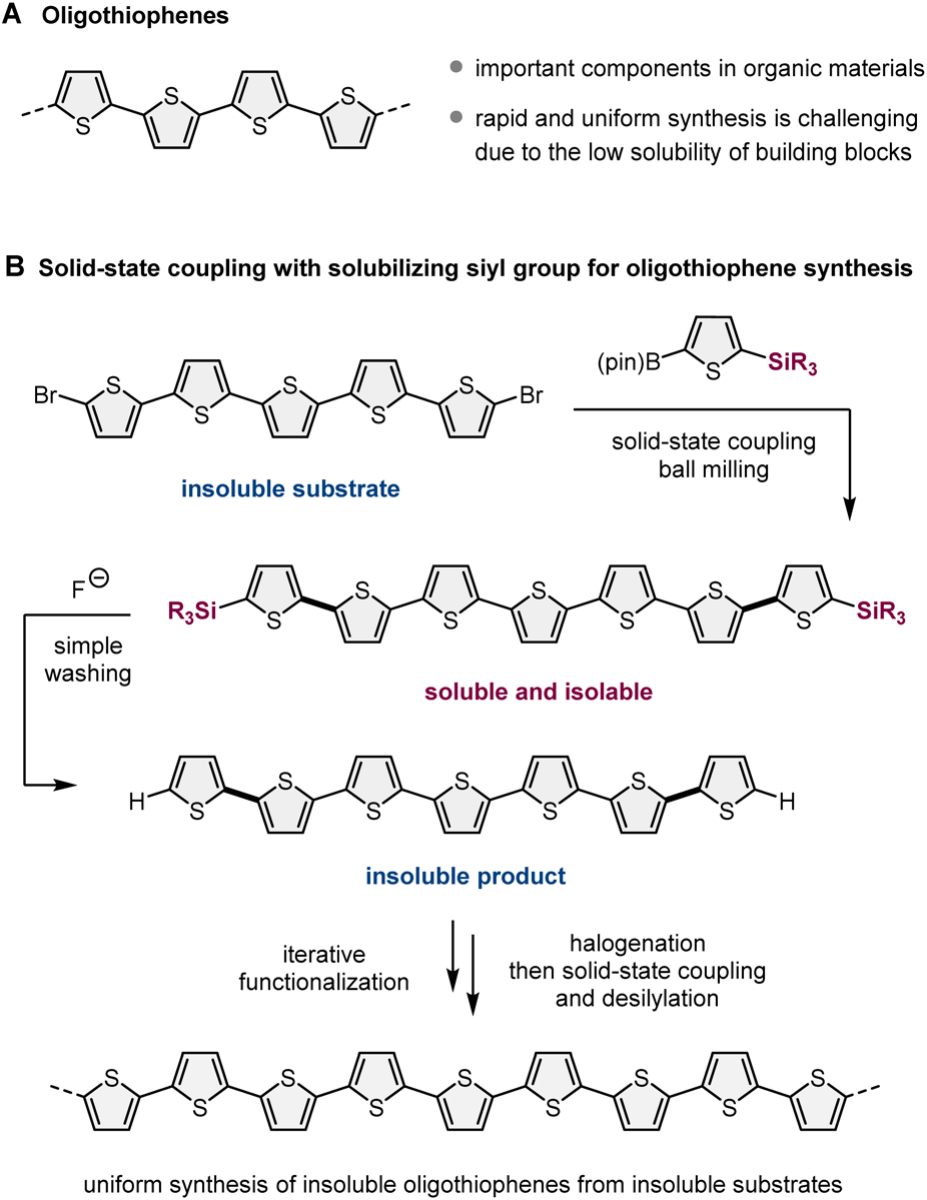
Uniform synthesis of nonsubstituted linear oligothiophenes and its derivatives *via* solid-state cross-coupling with a removable solubilizing silyl group.

For a proof-of-concept study, we selected oligothiophene and its derivatives as synthetic targets, which are important components in the design of organic materials, such as fluorescent materials, organic light-emitting devices, and photo-responsive anticancer agents (Scheme 2A).^11^ This choice was motivated by the following reasons (Scheme 2B): (1) the cross-coupling of halogenated oligothiophenes (>5 mers) is inefficient due to their significantly low solubility in organic solvents;^11^ (2) practical strategies for the uniform and systematic synthesis of relatively long linear oligothiophenes (>5 mers) are rare;^11^ (3) a silyl-based solubilizing group can be easily installed into thiophene-based boron nucleophiles *via* an iridium-catalyzed C–H silylation protocol;^12^ (4) desilylation of 2-silyl-substituted thiophenes is feasible under mild conditions;^13^ (5) an electrophilic bromination selectively occurs at the alpha-position of the sulfur atom.^14^ This mechanochemical strategy should enable the uniform synthesis of nonsubstituted linear oligothiophenes with various lengths and a diverse range of its derivatives, even if insoluble substrates and products are involved. Although Bäuerle *et al.* have reported the solution-based synthesis of 3D oligothiophene dendrons *via* an iterative synthetic approach using desilylation/cross-coupling sequences, the synthesized nonsubstituted 3D-dendrimeric oligothiophenes are soluble in organic solvents, and the insolubility of starting materials and reaction products was not a problem in their study.^13^ Despite some similarities between our strategy and theirs, the silyl group does not act as a solubilizing group in their study, and the essential object of our study is different.

## Results and discussion

**Scheme 3:**
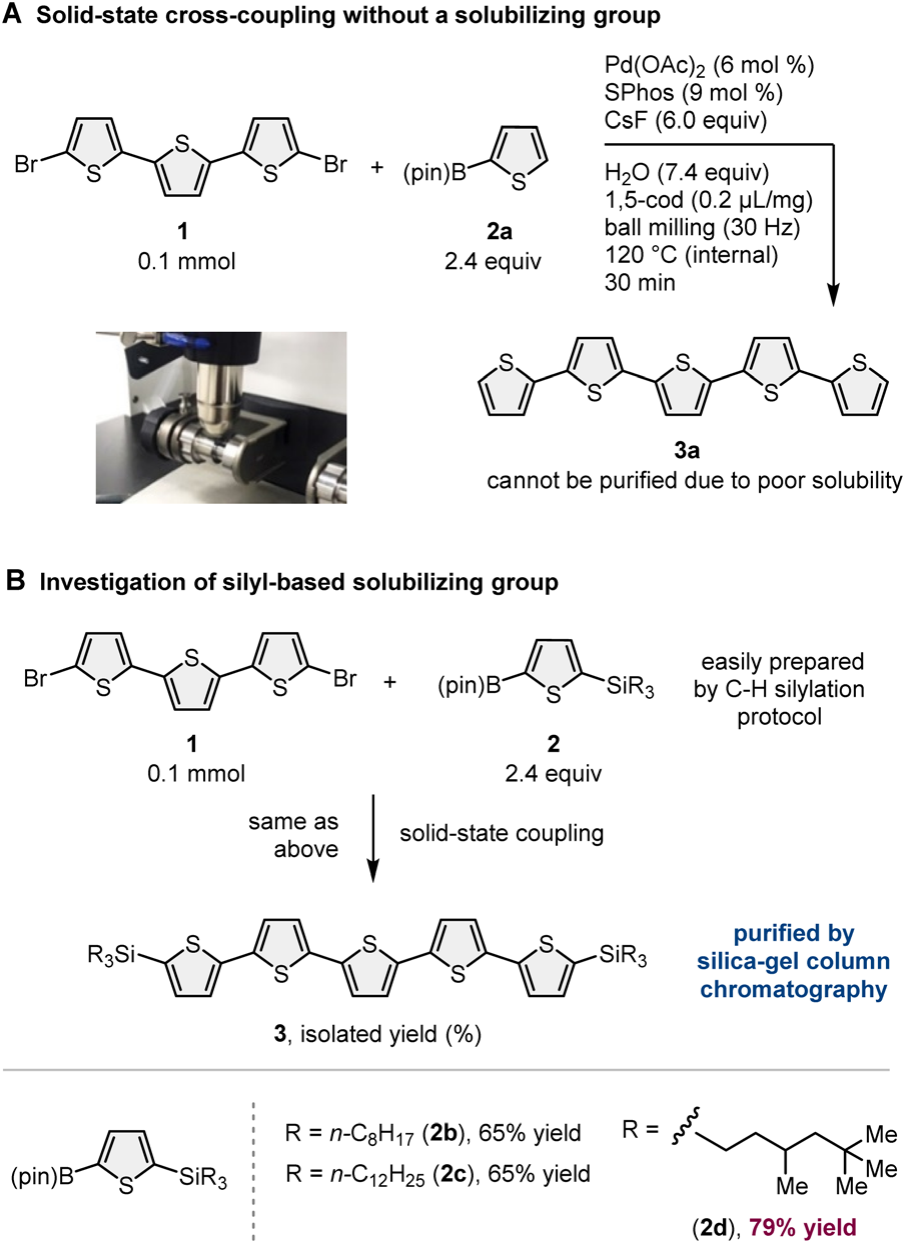
Optimization of silicon-based solubilizing group.

To demonstrate our synthetic strategy for the conversion of insoluble substrates into insoluble products, we began our study by optimizing a silicon-based solubilizing group that allowed the isolation of insoluble oligothiophene derivatives *via* silica gel column chromatography (Scheme 3). All mechanochemical reactions were conducted using a Retsch MM400 ball mill (stainless steel milling jar; stainless steel balls; 30 Hz). Initially, we investigated cross-coupling reactions of dibromo-terthiophene (1) with 2-boryl-thiophene without a solubilizing group (2a) in the presence of 6 mol% Pd(OAc)_2_, 6 mol% SPhos, CsF (3.0 equiv.), H_2_O (7.4 equiv.), and 1,5-cod (0.2 mL mg^−1^) as a liquid-assisted-grinding additive at 120 °C (temperature inside the jar) for 90 min.^3*a*^ We used a temperature-controllable heat gun to adjust the reaction temperature, which was confirmed by thermography immediately after opening the jar (see the ESI† for details).^3*a*^ As expected, the reaction proceeded, but the corresponding product 3a could not be isolated by silica gel column chromatography or recrystallization due to the significantly low solubility in organic solvent (Scheme 3A). We then prepared silylated thiophene-based boron nucleophile (Scheme 3B). The silyl group can be easily installed into 2a by an iridium-catalyzed C–H silylation reaction with the corresponding hydrosilanes.^11^ The solid-state coupling reaction with thienylboronate bearing a trioctylsilyl group (2b) afforded the desired bis-silylated quinquethiophene (3b) in good yield (65%). 3b is soluble in various organic solvents and can thus be isolated *via* silica gel column chromatography. The substrate bearing longer alkyl chains on the silyl group (2c) also provided the desired product 3c in good yield (65%). Interestingly, the introduction of a silyl group bearing branched alkyl chains (2d) further improved the efficiency, and the corresponding highly soluble product 3d was obtained in a higher yield (79%).

**Scheme 4:**
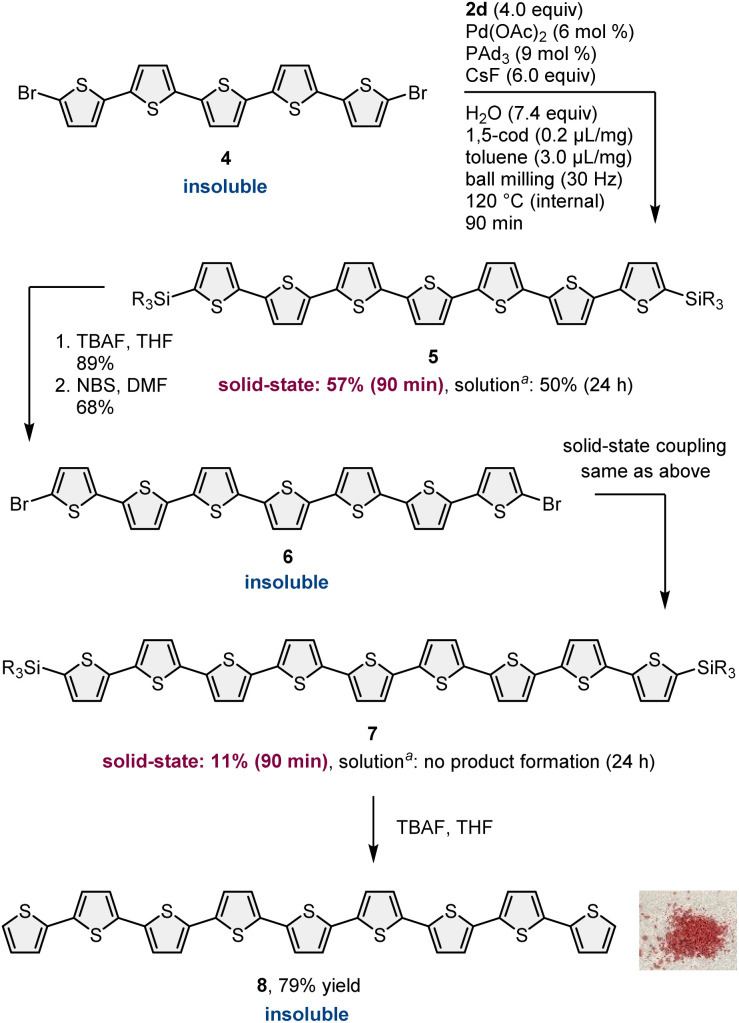
Solid-state cross-coupling of insoluble brominated substrates into insoluble oligothiophene products and the iterative approach for the first uniform synthesis of unsubstituted 9-*mer*-oligothiopene 8. ^*a*^The reaction was carried out as a slurry using toluene (1.0 mL) at 120 °C for 24 h.

After identifying the optimal solubilizing silyl group, the feasibility of the organic transformation of insoluble substrates into insoluble products was investigated (Scheme 4). Bis-silylated quinquethiophene (3d), which was obtained by the solid-state cross-coupling of 1, was subjected to a reaction with tetrabutylammonium fluoride (TBAF) and subsequent bromination with *N*-bromosuccinimide (NBS) to afford brominated quinquethiophene (4) in good yield (see the ESI† for details). Bromination can be performed under solution-based conditions and proceeds as a slurry. 4 is poorly soluble in organic solvents, with a solubility of <5 × 10^−5^ M in toluene at 23 °C; thus, its derivatization under solution conditions is inefficient. In fact, the cross-coupling between 4 and 2d in toluene at 120 °C provided the desired bis-silylated septithiophene 5 in moderate yield (50%) even after a long reaction time (24 h). Owing to the low solubility of 4, this solution-based reaction was performed as a slurry. In contrast, solid-state conditions using toluene as an additional liquid-assisted-grinding additive enabled the synthesis of 5 in higher yield (57%) in much shorter reaction times (90 min). Notably, coupling product 5 is soluble in common organic solvents and can be isolated by silica gel column chromatography. 5 was then subjected to further desilylation/bromination to form insoluble brominated septithiophene 6. Impurities derived from the silyl group were removed by washing with organic solvents, and the formation of 6 was confirmed by high resolution mass spectrometer (HRMS). Because of the significantly low solubility of 6, solution-based cross-coupling of 6 did not afford the corresponding coupling product 7. However, the solid-state cross-coupling reaction of 6 under high-temperature ball-milling conditions yielded 7 (11%). 7 was isolated by silica gel column chromatography. Desilylation of 7 with TBAF in solution allowed for the first uniform synthesis of nonsubstituted 9-*mer*-oligothiophene 8 as a red solid.

**Scheme 5:**
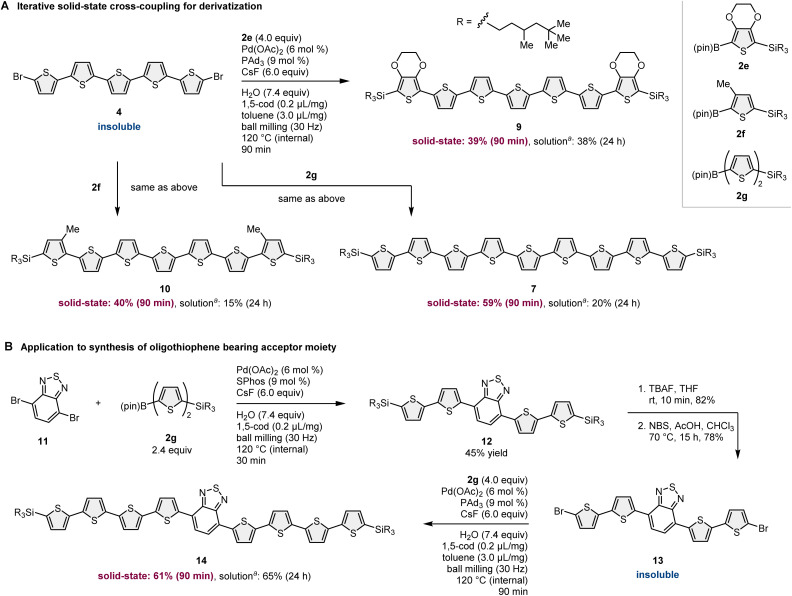
Solid-state cross-coupling approach for rapid derivatization of oligothiophenes. See the ESI† for the details of the reaction conditions. ^*a*^The reaction was carried out as a slurry using toluene (1.0 mL) at 120 °C for 24 h.

In addition to the synthesis of nonsubstituted linear oligothiophenes, this strategy has been applied to the derivatization of insoluble oligothiophene-based building blocks (Scheme 5A). Due to the low solubility of 4, the solution-based reactions of 4 with other 2-borylthiophene derivatives (2e–2g) resulted in very low yields of the corresponding products (7, 9, and 10; 15–38%) even after long reaction time. Although we tested many different solution-based conditions using different catalysts, solvents, and temperatures, the reaction efficiency did not improve. The solid-state mechanochemical conditions enabled the synthesis of oligothiophenes (7, 9, and 10) in higher yields (39–59%) compared to that under solution-based conditions with shorter reaction times (90 min). Notably, these silylated products were soluble in common organic solvents and could be isolated by silica gel column chromatography.

The developed iterative solid-state coupling methodology was further applied to the uniform synthesis of oligothiophenes bearing an acceptor moiety in the conjugated system (Scheme 5B).^11^ The solid-state cross-coupling of 11 with 2g proceeded smoothly to yield 12 in 45% yield. Subsequent desilylation/debromination provided the bis-brominated oligothiophene 13. 13 is hardly soluble in organic solvents; thus, cross-coupling between 13 and 2g under solution-based conditions required a long reaction time (24 h) to obtain a synthetically acceptable yield (65%). However, solid-state conditions enabled the synthesis of 14 in good yield (61%) in a much shorter reaction time (90 min), showing the better efficiency of the mechanochemical cross-coupling approach than that of conventional solution-based conditions.

**Fig. 1:**
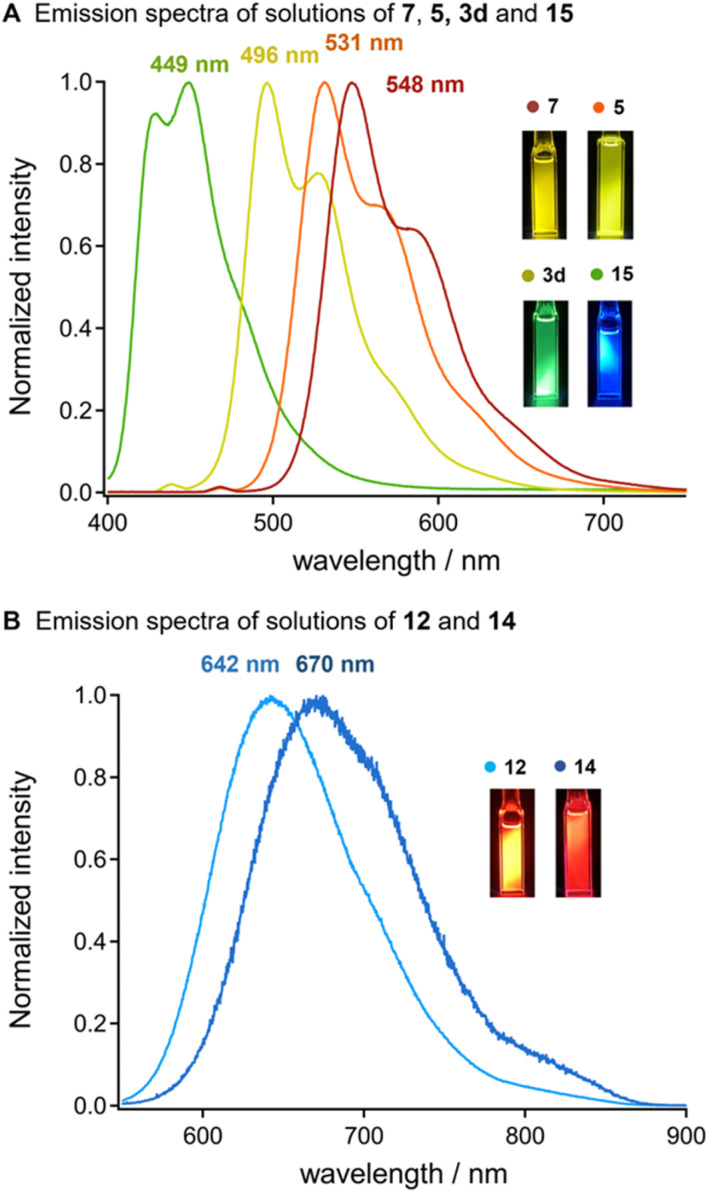
Optical properties of the newly synthesized oligothiophenes. (A) Emission spectra of the solutions of 3d (*c* = 4.1 × 10^−6^ M; *λ*_ex_ = 373 nm), 5 (*c* = 4.8 × 10^−6^ M; *λ*_ex_ = 373 nm), 7 (*c* = 3.3 × 10^−6^ M; *λ*_ex_ = 373 nm), and 15 (*c* = 3.6 × 10^−5^ M; *λ*_ex_ = 373 nm) in CHCl_3_. (B) Emission spectra of the solutions of 12 (*c* = 5.8 × 10^−6^ M; *λ*_ex_ = 373 nm) and 14 (*c* = 2.5 × 10^−6^ M; *λ*_ex_ = 373 nm) in CHCl_3_.

Oligothiophenes have served functional building block for developing emission property.^15^ We observed that the uniformly synthesized oligothiophenes bearing silyl groups exhibited strong photoluminescence in chloroform under ultraviolet (UV) irradiation (Fig. 1). A dilute solution of bis-silylated terthiophene (14) in chloroform showed strong blue emission with the maximum emission wavelength (*λ*_em, max_) at 449 nm, excited by UV light (*λ*_ex_ = 370 nm). A clear red shift in luminescence was observed with the elongation of the thiophene units. Silylated 9-*mer*-oligothiophene 7 showed a strong yellow emission (*λ*_em, max_ = 548 nm), excited by a light having 469 nm of *λ*_ex_. In addition, the silylated oligothiophenes with an acceptor moiety (12 and 14) showed a significant red shift in their emissions to the near-infrared region (*λ*_em_ = 642 and 670 nm, respectively) under the excitation lights (*λ*_ex_ = 500 and 530 nm, respectively). These results suggest that the diversification of oligothiophenes *via* our iterative solid-state coupling strategy allows for the development of new emissive organic materials with desirable photophysical properties.^11,15^

## Conclusions

In synthetic organic chemistry, the development of organic reactions for the conversion of insoluble compounds into insoluble products remains unexplored. This is due to the significantly low reactivity of insoluble substrates under conventional solution-based conditions and the difficulty in isolating the corresponding insoluble products with sufficient purity. These solubility issues significantly limit the scope of the synthesizable organic molecules and represent an unresolved challenge in organic synthesis. In this study, we developed a new solid-state mechanochemical strategy using a removable and solubilizing silyl group that enables the organic transformation of insoluble substrates into insoluble products. The utility of this approach was demonstrated by the rapid, efficient, and uniform synthesis of relatively long nonsubstituted linear oligothiophenes and their derivatives, which are difficult to prepare under conventional solution-based conditions. Notably, we achieved the first uniform synthesis of the longest nonsubstituted insoluble linear 9-*mer*-oligothiophene. In addition to its immediate utility in the synthesis of oligothiophene-based materials, our mechanochemical approach can be applied to the preparation of other classes of insoluble polyaromatic compounds, allowing for the discovery of new organic materials with interesting properties.

## Conflicts of interest

There are no conflicts to declare.

## Supplementary Material

RA-013-D3RA05571J-s001
