# Supplementary material for: Solid-state mechanochemical cross-coupling of insoluble substrates into insoluble products by removable solubilizing silyl groups: uniform synthesis of nonsubstituted linear oligothiophenes
Source: RSC Adv. 2023 Sep 29;13(41):28652–7. doi: 10.1039/d3ra05571j (PMC10540273; doi:10.1039/d3ra05571j)
Supplement: RA-013-D3RA05571J-s001 [file RA-013-D3RA05571J-s001.pdf]

Electronic Supplementary Material (ESI) for RSC Advances.
This journal is © The Royal Society of Chemistry 2023

Solid-state mechanochemical cross-coupling of insoluble substrates into insoluble products by removable solubilizing silyl groups: Uniform synthesis of nonsubstituted linear oligothiophenes

Koji Kubota*[a,b], Keisuke Kondo[a], Tamae Seo[a] , Mingoo Jin[b], and Hajime Ito*[a,b]

[a]*Division of Applied Chemistry, Graduate School of Engineering, Hokkaido University, Sapporo, Hokkaido, 060-8628, Japan.*

[b]*Institute for Chemical Reaction Design and Discovery (WPI-ICReDD), Hokkaido University, Sapporo, Hokkaido 060-8628, Japan.*

e-mail: hajito@eng.hokudai.ac.jp, kbt@eng.hokudai.ac.jp

Table of contents

1. **Chemicals and instrumentation**
2. **Substrate preparation**
3. **Example procedure for solid-state cross-coupling**
4. **Example procedure for cross-coupling reactions in solution**
5. **Example procedure for bromination**
6. **Example procedure for desilylation**
7. **Control experiments**
8. **Thermography observation for reaction temperature**
9. **Optical properties**
10. **References**
11. **NMR Spectra**

1. Chemicals and instrumentation

Materials were obtained from commercial suppliers and used as received. Solvents were also purchased from commercial suppliers and further dried over molecular sieves (MS 4A). All mechanochemical reactions were carried out using grinding vessels in a Retsch MM400 mill (Figure S1). Both jars (1.5 mL and 5.0 ml) and balls (5 mm, 10 mm) are made of stainless (SUS400B and SUS420J2, respectively) (Figure S2). The heat gun Takagi HG-1450B with a temperature control function was used for high-temperature ball-milling reactions (Figure S3). NMR spectra were recorded on JEOL JNM-EC X400P and JNM-ECS400 spectrometers (^{1}H: 392 or 396 or 399 or 401 MHz, ^{13}C: 99 or 100 MHz). Tetramethylsilane (^{1}H), $CDCl_3$ (^{13}C) were employed as external standards, respectively. Multiplicity was recorded as follows: s = singlet, brs = broad singlet, d = doublet, t = triplet, q = quartet, quint = quintet, sept = septet, o = octet, m = multiplet. 1,1,2,2-Tetrachloroethane was used as an internal standard to determine NMR yields. High-resolution mass spectra were recorded at the Global Facility Center, Hokkaido University, and GC-MS & NMR Laboratory, Research Faculty of Agriculture, Hokkaido University. Recycle preparative gel permeation chromatography (GPC) was conducted with a JAI LaboACE LC-5060 using $CHCl_3$ as an eluent with JAIGEL-1H. Absorption spectra were recorded on a Hitachi U-2910 spectrometer. Emission spectra were recorded on a Hitachi F-7000 spectrometer.

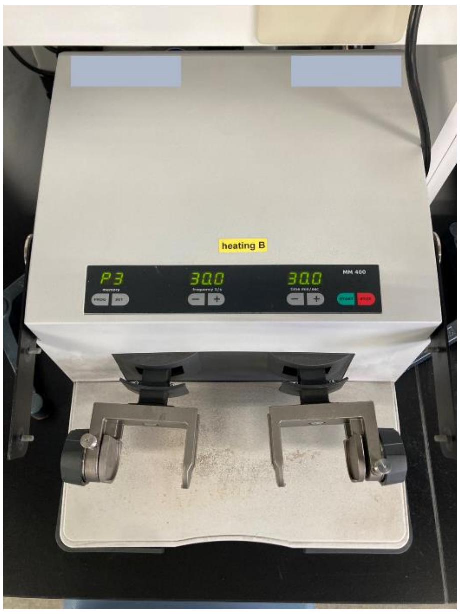

Figure S1. Retsch MM400 used in this study.

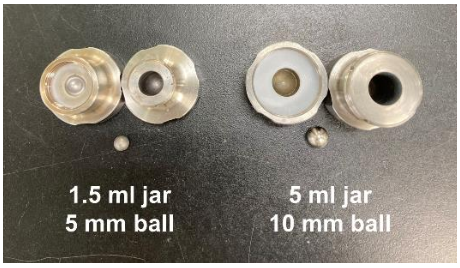

Figure S2. Stainless jar (1.5 mL and 5 ml) and ball (5 mm and 10 mm) used in this study.

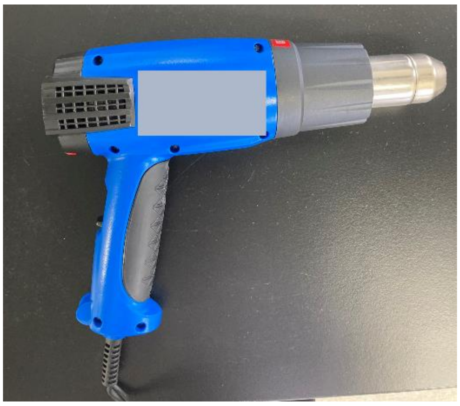

Figure S3. The temperature-controllable heat gun Takagi HG-1450B used in this study.

2. Substrate preparation

Preparation of 1-bromo-3,5,5-trimethylhexane.

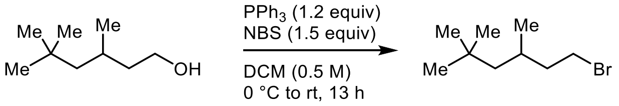

In a vacuum dried 50 mL three-necked round-bottomed flask, 3,5,5-trimethylhexan-1-ol (1.44g, 10 mmol) and triphenylphosphine (3.17 g, 12 mmol) were dissolved in dichloromethane (20 ml) and cooled to 0 °C. To the mixture, *N*-bromosuccinimide (NBS) (2.77 g, 15 mmol) was added slowly, then stirred at room temperature for 13 hours. After evaporation of solvents in vacuo, the residue was dissolved with hexane, and the solid material was filtered off through a pad of Celite and washed with hexane. The crude mixture was then purified by silica-gel column chromatography (hexane only) to give 1-bromo-3,5,5-trimethylhexane (1.27 g, 6.1 mmol, 61% yield) as a colorless oil. ^{1}H and ^{13}C NMR were in agreement with the literature.[1]

^{1}H NMR (399 MHz, $CDCl_3$, δ): 0.85–0.94 (m, 12H), 1.06–1.23 (m, 2H), 1.63–1.75 (m, 2H), 1.81–1.92 (m, 1H), 3.36–3.47 (m, 2H).^{13}C NMR (99 MHz, $CDCl_3$, δ): 22.1 (CH$_3$), 28.2 (CH), 30.1 (CH$_3$), 31.3 (C), 32.4 (CH$_2$), 42.4 (CH$_2$), 50.9 (CH$_2$). HRMS-EI (*m*/*z*): [M-Me]$^+$ calcd for $C_8H_{16}Br$, 191.0435; found, 191.0432.

Preparation of tris(3,5,5-trimethylhexyl)silane.

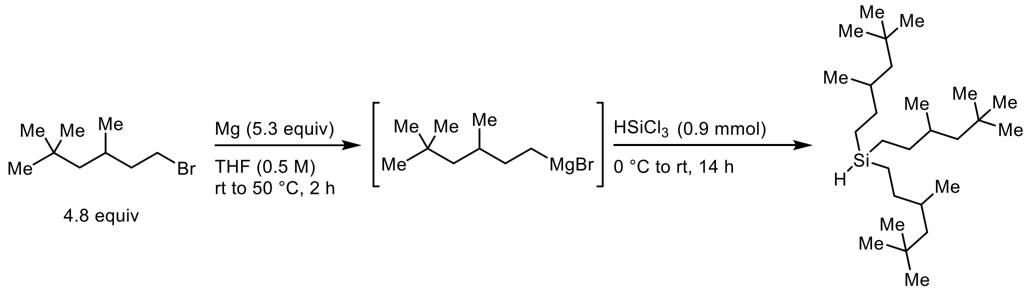

In a vacuum dried 50 mL two-necked round bottomed flask, magnesium (130.8 mg, 5.4 mmol) and a piece of iodine crystal were added to 10 mL anhydrous THF under a nitrogen atmosphere. 1-Bromo-3,5,5-trimethylhexane (985.9 mg, 4.8 mmol) was added over 30 min, and the mixture was warmed up to 50 °C. Then, the mixture was stirred for 2 h to yield the Grignard reagent. The prepared Grignard reagent was transferred into the other vacuum dried 100 mL two-necked round-bottomed flask. Trichlorosilane (121.0 mg, 0.89 mmol) was added slowly to a solution of the Grignard reagent at 0 °C, and then the mixture was then warmed to room temperature. After stirring for 14 h, the resulting suspension was quenched by the addition of saturated NH_4Cl aqueous solution. The mixture was extracted with Et_2O three times and dried over Mg_2SO_4. After filtration, the solvents were removed using a rotary evaporator. The residue was purified by silica-gel column chromatography (hexane

only) and recycling preparative GPC to give the corresponding silane (353.6 mg, 0.86 mmol, 96%) as a colorless oil.

^{1}H NMR (396 MHz, $CDCl_3$, δ): 0.47–0.64 (m, 6H), 0.77–0.95 (m, 36H), 0.97–1.05 (m, 3H), 1.09–1.46 (m, 12H), 3.61–3.68 (m, 1H). ^{13}C NMR (100 MHz, $CDCl_3$, δ): 8.5 (*C*H_2), 22.4 (*C*H_3), 30.3 (*C*H_3), 31.2 (*C*H), 32.3 (*C*H_2), 34.3 (*C*), 51.0 (*C*H_2). HRMS-FD (*m*/*z*): $[M]^+$ calcd for $C_{27}H_{58}Si$, 410.4308; found, 410.4292.

Preparation of tridodecylsilane.

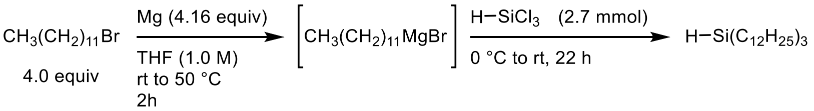

In a vacuum dried 50 mL two-necked round-bottomed flask, magnesium (278.7 mg, 11.5 mmol) and a piece of iodine crystal were added to 11 mL anhydrous THF under a nitrogen atmosphere. 1-Bromododecane (2.72 g, 10.9 mmol) was added over 30 min, and the mixture was warmed up to 50 °C. Then, the mixture was stirred for 2 h to yield the Grignard reagent. The prepared Grignard reagent was transferred into the other vacuum dried 50 mL one-necked round-bottomed flask. Trichlorosilane (370.1 mg, 2.7 mmol) was added slowly to a solution of the Grignard reagent at 0 °C, and then the mixture was warmed to room temperature. After stirring for 22 h, the resulting suspension was quenched by the addition of saturated NH_4Cl aqueous solution. The mixture was extracted with hexane three times and dried over Mg_2SO_4. After filtration, the solvents were removed using a rotary evaporator. The residue was purified by silica-gel column chromatography (hexane only) and recycling preparative GPC to give the corresponding silane (1.27 g, 2.4 mmol, 83%) as a colorless oil.

^{1}H NMR (396 MHz, $CDCl_3$, δ): 0.52–0.62 (m, 6H), 0.88 (t, *J* = 6.7 Hz, 9H), 1.20–1.39 (m, 60H), 3.62–3.69 (m, 1H). ^{13}C NMR (100 MHz, $CDCl_3$, δ): 11.5 (*C*H_2), 14.3 (*C*H_3), 22.9 (*C*H_2), 24.9 (*C*H_2), 29.6 (*C*H_2), 29.8 (*C*H_2), 29.9 (*C*H_2), 30.0 (*C*H_2), 32.2 (*C*H_2), 33.6 (*C*H_2). HRMS-FD (*m*/*z*): $[M]^+$ calcd for $C_{36}H_{76}Si$, 536.5716; found, 536.5715.

Example procedure for C–H silylation.

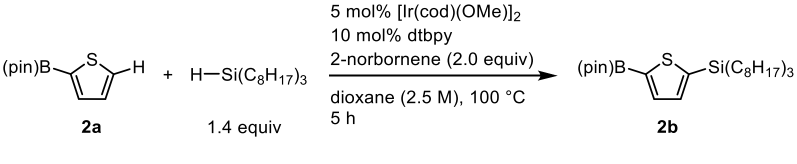

The reaction was performed according to the literature procedure.[2,3] **2a** (105.2 mg, 0.50 mmol), [Ir(cod)$(OMe)_2$] (16.5 mg, 0.025 mmol) and 4,4'-di-*tert*-butyl-2,2'-bipyridyl (dtbpy) (13.9 mg, 0.05 mmol) were placed in an oven-dried reaction vial. After the vial was sealed with a screw cap containing a Teflon-coated rubber septum, the vial was connected to a vacuum/nitrogen manifold through a

needle. It was evacuated and then backfilled with nitrogen. This cycle was repeated three times. 1,4-Dioxane (0.2 mL), 2-norbornene (94.7 mg, 1.0 mmol), and trioctylsilane (251.3 mg, 0.68 mmol) were added in the vial at room temperature. The resulting mixture was allowed to warm at 100 °C and stirred for 5 h. After the reaction, the mixture was passed through a short silica gel column eluting with Et_2O. The crude mixture was then purified by flash column chromatography (SiO_2, EtOAc/hexane, 0:100–10:90) to give **2b** as a brown oil (141 mg, 0.24 mmol, 49% yield).
^{1}H NMR (399 MHz, $CDCl_3$, δ): 0.74–0.82 (m, 6H), 0.87 (t, J = 7.0 Hz, 9H), 1.18–1.39 (m, 36H), 1.35 (s, 12), 7.30 (d, J = 3.2 Hz, 1H), 7.70 (d, J = 3.6 Hz, 1H). ^{13}C NMR (100 MHz, $CDCl_3$, δ): 13.6 (CH$_2$), 14.3 (CH$_3$), 22.8 (CH$_2$), 23.8 (CH$_2$), 24.9 (CH$_3$), 29.3 (CH$_2$), 29.4 (CH$_2$), 32.1 (CH$_2$), 33.8 (CH$_2$) 84.1 (C), 135.7 (CH), 137.9 (CH), 146.1 (C). HRMS-ESI (m/z): $[M+Na]^+$ calcd for $C_{34}H_{65}O_2BNaSSi$, 598.4496; found, 598.4493.

Tridodecyl[5-(4,4,5,5-tetramethyl-1,3,2-dioxaborolan-2-yl)thiophen-2-yl]silane (2c).

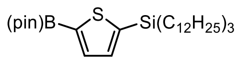

2c

The reaction was carried out with 105.6 mg (0.50 mmol) of **2a** and 333.0 mg (0.62 mmol) of tridodecylsilane. **2c** was obtained as a brown oil (188 mg, 0.25 mmol, 50% yield) after purification by silica-gel column chromatography (SiO_2, EtOAc/hexane, 0:100–10:90).
^{1}H NMR (396 MHz, $CDCl_3$, δ): 0.73–0.82 (m, 6H), 0.88 (t, J = 6.5 Hz, 9H), 1.18–1.40 (m, 60H), 1.35 (s, 12), 7.30 (d, J = 3.2 Hz, 1H), 7.70 (d, J = 3.2 Hz, 1H). ^{13}C NMR (100 MHz, $CDCl_3$, δ): 13.6 (CH$_2$), 14.3 (CH$_3$), 22.9 (CH$_2$), 23.9 (CH$_2$), 24.9 (CH$_3$), 29.4 (CH$_2$), 29.6 (CH$_2$), 29.78 (CH$_2$), 29.85 (CH$_2$), 29.9 (CH$_2$), 32.1 (CH$_2$), 33.9 (CH$_2$), 84.1 (C), 135.7 (CH), 137.9 (CH), 146.1 (C). HRMS-ESI (m/z): $[M+Na]^+$ calcd for $C_{46}H_{89}O_2BNaSSi$, 766.6374; found, 766.6369.

[5-(4,4,5,5-Tetramethyl-1,3,2-dioxaborolan-2-yl)thiophen-2-yl]tris(3,5,5-trimethylhexyl)silane (2d).

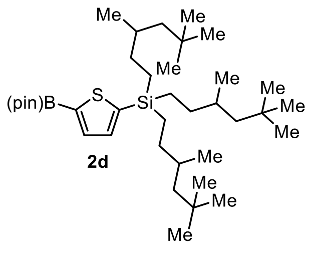

2d

The reaction was carried out with 419.9 mg (2.0 mmol) of **2a** and 1264.2 mg (3.1 mmol) of tris(3,5,5-trimethylhexyl)silane. **2d** was obtained as a brown oil (935 mg, 1.5 mmol, 76% yield) after purification by silica-gel column chromatography (SiO_2, EtOAc/hexane, 0:100–10:90).

^{1}H NMR (392 MHz, $CDCl_3$, δ): 0.67–0.94 (m, 42H), 0.94–1.04 (m, 3H), 1.07–1.45 (m, 12H), 1.35 (s, 12), 7.30 (d, *J* = 3.5 Hz, 1H), 7.70 (d, *J* = 3.1 Hz, 1H). ^{13}C NMR (100 MHz, $CDCl_3$, δ): 10.5 (CH_2), 22.3 (CH_3), 24.9 (CH_3), 30.2 (CH_3), 31.1 (*C*H), 32.4 (CH_2), 33.1 (*C*), 50.8 (CH_2), 84.0 (*C*), 135.6 (*C*H), 137.9 (*C*H), 145.7 (*C*). HRMS-ESI (m/z): $[M+Na]^+$ calcd for $C_{37}H_{71}O_2BNaSSi$, 640.4966; found, 640.4954.

[7-(4,4,5,5-Tetramethyl-1,3,2-dioxaborolan-2-yl)-2,3-dihydrothieno[3,4-b][1,4]dioxin-5-yl]tris(3,5,5-trimethylhexyl)silane (2e).

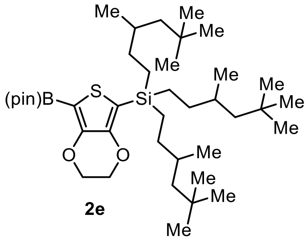

2e

The reaction was carried out with 133.5 mg (0.50 mmol) of 2-(2,3-dihydrothieno[3,4-b][1,4]dioxin-5-yl)-4,4,5,5-tetramethyl-1,3,2-dioxaborolane and 309.9 mg (0.75 mmol) of tris(3,5,5-trimethylhexyl)silane. **2e** was obtained as a brown oil (188 mg, 0.28 mmol, 56% yield) after purification by silica-gel column chromatography (SiO_2, EtOAc/hexane, 0:100–10:90).
^{1}H NMR (399 MHz, $CDCl_3$, δ): 0.68–0.94 (m, 42H), 0.94–1.04 (m, 3H), 1.07–1.44 (m, 12H), 1.34 (s, 12), 4.08–4.16 (m, 2H), 4.22–4.28 (m, 2H). ^{13}C NMR (99 MHz, $CDCl_3$, δ): 9.6 (CH_2), 22.2 (CH_3), 24.8 (CH_3), 30.2 (CH_3), 31.1 (*C*H), 32.3 (CH_2), 33.0 (*C*), 50.8 (CH_2), 64.1 (CH_2), 64.9 (CH_2), 83.7 (*C*), 118.6 (*C*), 147.6 (*C*), 149.4 (*C*). HRMS-ESI (m/z): $[M+Na]^+$ calcd for $C_{39}H_{73}O_4BNaSSi$, 698.5020; found, 698.5011.

[4-Methyl-5-(4,4,5,5-tetramethyl-1,3,2-dioxaborolan-2-yl)thiophen-2-yl]tris(3,5,5-trimethylhexyl)silane (2f).

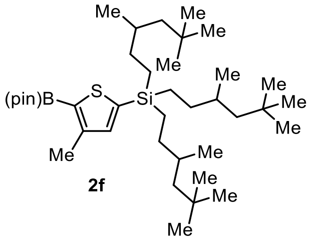

2f

The reaction was carried out with 112.0 mg (0.50 mmol) of 4,4,5,5-tetramethyl-2-(3-methylthiophen-2-yl)-1,3,2-dioxaborolane and 303.5 mg (0.74 mmol) of tris(3,5,5-trimethylhexyl)silane. **2f** was obtained as a brown oil (207 mg, 0.33 mmol, 65% yield) after purification by silica-gel column chromatography (SiO_2, EtOAc/hexane, 0:100–10:90).

^{1}H NMR (399 MHz, CDCl$_3$, δ): 0.65–0.93 (m, 42H), 0.95–1.04 (m, 3H), 1.09–1.43 (m, 12H), 1.33 (s, 12), 2.47 (s, 3H), 7.07 (s, 1H). ^{13}C NMR (100 MHz, CDCl$_3$, δ): 10.5 (*C*H$_2$), 15.9 (*C*H$_3$), 22.3 (*C*H$_3$), 25.0 (*C*H$_3$), 30.2 (*C*H$_3$), 31.2 (*C*), 32.4 (*C*H$_3$), 33.1 (*C*H$_2$), 50.8 (*C*H$_2$), 83.6 (*C*), 138.9 (*C*H), 144.7 (*C*), 149.7 (*C*). HRMS-ESI (m/z): [M+Na]$^+$ calcd for $C_{38}H_{73}O_2BNaSSi$, 654.5122; found, 698.5110.

[5'-(4,4,5,5-Tetramethyl-1,3,2-dioxaborolan-2-yl)-[2,2'-bithiophen]-5-yl]tris(3,5,5-trimethylhexyl)silane (2g).

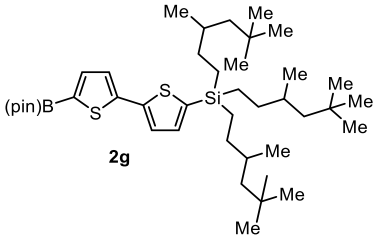

2g

The reaction was carried out with 147.6 mg (0.51 mmol) of 2-([2,2'-bithiophen]-5-yl)-4,4,5,5-tetramethyl-1,3,2-dioxaborolane and 308.7 mg (0.75 mmol) of tris(3,5,5-trimethylhexyl)silane. **2g** was obtained as a brown oil (206 mg, 0.29 mmol, 58% yield) after purification by silica-gel column chromatography (SiO$_2$, EtOAc/hexane, 0:100–10:90).

^{1}H NMR (392 MHz, CDCl$_3$, δ): 0.48–0.95 (m, 42H), 0.97–1.05 (m, 3H), 1.10–1.46 (m, 12H), 1.35 (s, 12), 7.11 (d, J = 3.1 Hz, 1H), 7.25 (d, J = 3.5 Hz, 1H), 7.29 (d, J = 3.1 Hz, 1H), 7.52 (d, J = 3.5 Hz, 1H). ^{13}C NMR (99 MHz, CDCl$_3$, δ): 10.5 (*C*H$_2$), 22.3 (*C*H$_3$), 24.9 (*C*H$_3$), 30.2 (*C*H$_3$), 31.2 (*C*H), 32.4 (*C*H$_2$), 33.2 (*C*), 50.8 (*C*H$_2$), 84.2 (*C*), 125.1 (*C*H), 125.6 (*C*H), 135.5 (*C*H), 137.9 (*C*), 138.1 (*C*H), 142.3 (*C*), 144.4 (*C*). HRMS-ESI (m/z): [M+Na]$^+$ calcd for $C_{41}H_{73}O_2BNaS_2Si$, 722.4843; found, 722.4822.

3. Example procedure for solid-state cross-coupling

1) Procedure A

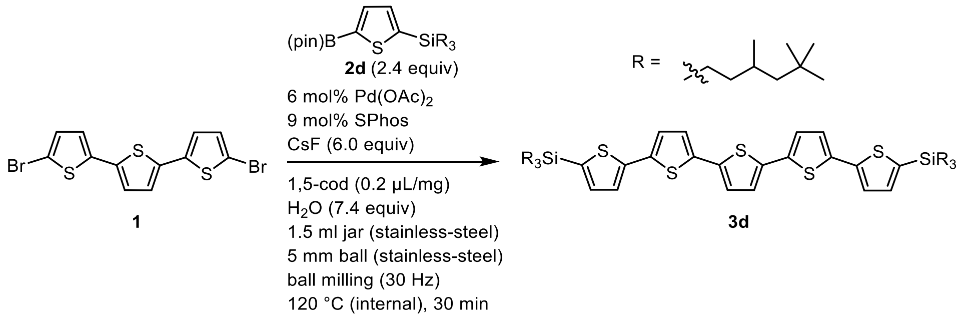

1 (80.9 mg, 0.2 mmol), **2d** (299.6 mg, 0.24 mmol), $Pd(OAc)_2$ (2.7 mg, 0.012 mmol), and SPhos (7.3 mg, 0.018 mmol), CsF (183.3 mg, 1.2 mmol) were placed in a ball milling vessel (stainless, 1.5 mL) loaded with one grinding ball (stainless, diameter: 5 mm) under air. Then, H_2O (27 μL, 7.4 equiv) and 1,5-cod (0.2 μL/mg) were added via a syringe. After the vessel was closed without purging with inert gas, the vessel was placed in the ball mill (Retsch MM400, 30 min at 30 Hz) and a heat gun (the preset temperature at 250 °C). After 30 min, the mixture was passed through a short silica gel column eluting with CH_2Cl_2 to remove inorganic salts. The crude mixture was then purified by flash column chromatography (SiO_2, CH_2Cl_2/hexane, 0:100–10:90) to give the corresponding coupling product **3d** as a yellow solid (166.0 mg, 0.14 mmol, 68% yield).

^{1}H NMR (392 MHz, $CDCl_3$, δ): 0.68–0.94 (m, 84H), 0.98–1.06 (m, 6H), 1.11–1.47 (m, 24H), 7.06–7.14 (m, 8H), 7.24 (d, J = 3.9 Hz, 2H). ^{13}C NMR (100 MHz, $CDCl_3$, δ): 10.3 (CH_2), 22.1 (CH_3), 30.0 (CH_3), 31.0 (C), 32.2 (CH), 33.0 (CH_2), 50.7 (CH_2), 124.1 (CH), 124.2 (CH), 124.3 (CH), 124.8 (CH), 135.3 (CH), 135.7 (C), 135.9 (C), 136.4 (C), 137.3 (C), 141.9 (C). HRMS-APCI (m/z): $[M+H]^+$ calcd for $C_{74}H_{125}S_5Si_2$, 1229.7918; found, 1229.7900. mp 65–68 °C.

2) Procedure B

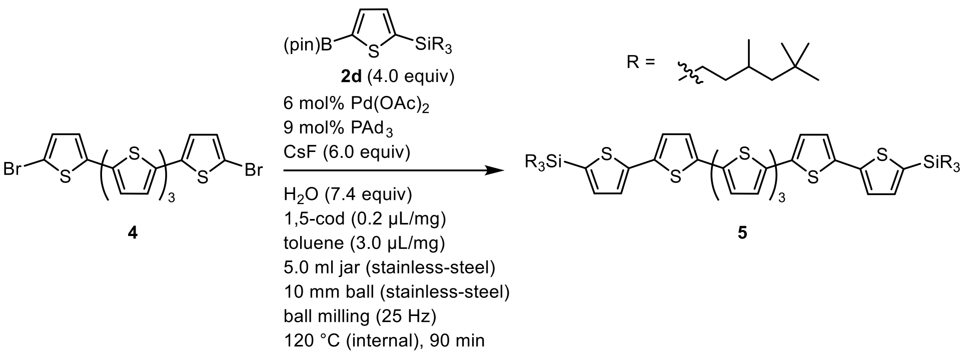

4 (57.1 mg, 0.1 mmol), **2d** (251.6 mg, 0.41 mmol), $Pd(OAc)_2$ (1.3 mg, 0.006 mmol), and PAd_3 (4.0 mg, 0.009 mmol), CsF (90.6 mg, 0.6 mmol) were placed in a ball milling vessel (stainless, 5.0 mL) loaded with one grinding ball (stainless, diameter: 10 mm) in air. Then, H_2O (13 μL, 7.4 equiv), toluene (3.0 μL/mg), and 1,5-cod (0.2 μL/mg) were added via a syringe. After the vessel was closed without purging with inert gas, the vessel was placed in the ball mill (Retsch MM400, 90 min at 25 Hz) and a heat gun (the preset temperature at 250 °C). After 90 min, the mixture was passed through a short silica gel column eluting with CH_2Cl_2 to remove inorganic salts. The crude mixture was then purified by flash column chromatography (SiO_2, CH_2Cl_2/hexane, typically 0:100–10:90) and recycling preparative GPC to give the corresponding coupling product **5** as an orange solid (80.0 mg, 0.057 mmol, 57% yield).

^{1}H NMR (401 MHz, $CDCl_3$, δ): 0.69–0.94 (m, 84H), 0.98–1.05 (m, 6H), 1.13–1.46 (m, 24H), 7.07–7.13 (m, 12H), 7.24 (d, J = 3.6 Hz, 2H). ^{13}C NMR (99 MHz, $CDCl_3$, δ): 10.5 (CH$_2$), 22.3 (CH$_3$), 30.2 (CH$_3$), 31.2 (C), 32.4 (CH), 33.2 (CH$_2$), 50.9 (CH$_2$), 124.4 (CH), 124.5 (CH), 125.0 (CH), 135.5 (CH), 135.8 (C), 135.9 (C), 136.1 (C), 136.3 (C), 136.7 (C), 137.6 (C), 142.0 (C). HRMS-APCI (m/z): $[M+H]^+$ calcd for $C_{82}H_{129}S_7Si_2$, 1393.7672; found, 1393.7663. mp 91–95 °C.

The heat gun was fixed with clamps and placed directly above the ball milling jar (distance between the heat gun and ball milling jar: ca. 1 cm) (Figure S4). The set-up procedure for high-temperature ball-milling reactions is shown in Figure S5. First, one grinding ball (stainless, diameter: 5 mm or 10 mm) was loaded in a ball milling jar (stainless, 1.5 mL, or 5 mL). Then solid and liquid materials were added to the jar. After the ball-milling jar was closed, the jar was placed in the ball mill (Retsch MM400), and a heat gun was placed directly above the ball-milling jar. The mechanochemical cross-coupling reactions were conducted while applying heated air to the outside of the milling jar (the preset temperature at 250 °C)

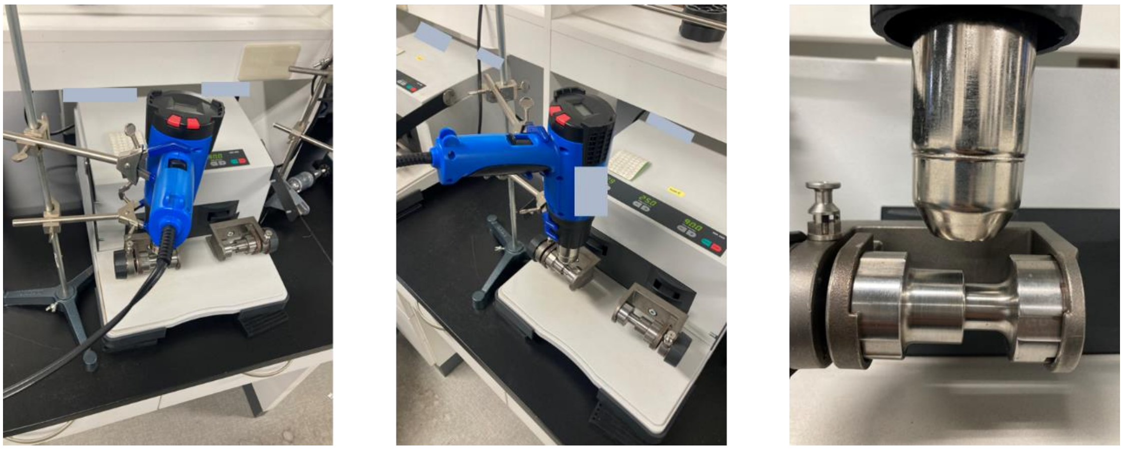

Figure S4. The set-up procedure for a heat gun on MM400.

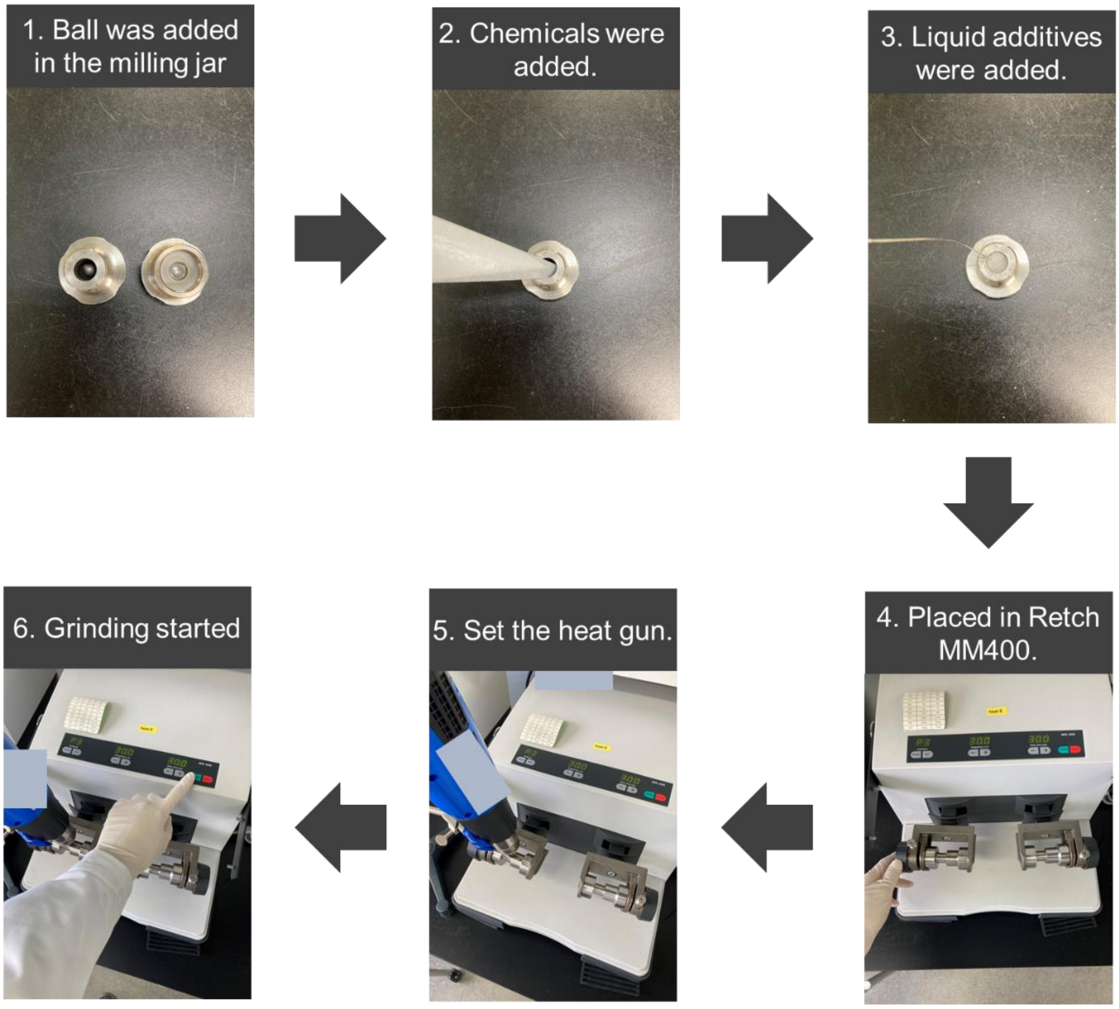

,

Figure S5. The set-up procedure for the high-temperature solid-state cross-coupling.

Disilylated novithiophene (7).

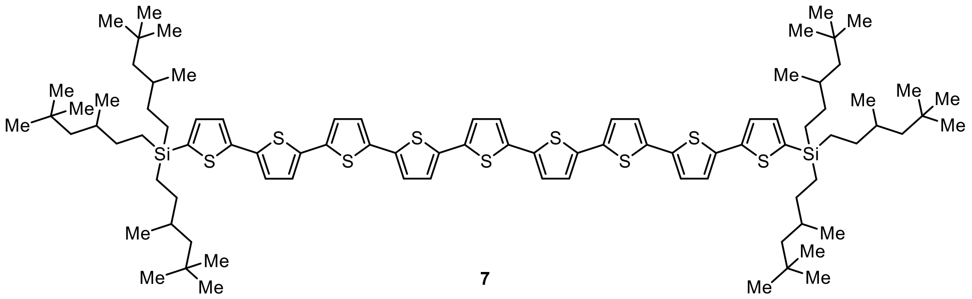

7

7 was synthesized from two different pathways, as described in the main text.

Path 1: The reaction was performed according to the example procedure B from **4**. The reaction was performed with 57.1 mg (0.1 mmol) of **4** and 288.0 mg (0.41 mmol) of **2g**. **7** was obtained as a red solid (91.8 mg, 0.059 mmol, 59% yield) after purification by silica-gel column chromatography (SiO_2, CH_2Cl_2/hexane, 0:100–10:90) and recycle preparative GPC.

Path 2: The reaction was performed according to the general procedure B from **6**. The reaction was carried out with 73.5 mg (0.1 mmol) of **6** and 251.3 mg (0.41 mmol) of **2d**. **7** was obtained as a red solid (16.9 mg, 0.011 mmol, 11% yield) after purification by silica-gel column chromatography (SiO_2, CH_2Cl_2/hexane, 0:100–10:90) and recycle preparative GPC.

^{1}H NMR (392 MHz, $CDCl_3$, δ): 0.67–0.97 (m, 84H), 0.97–1.07 (m, 6H), 1.12–1.47 (m, 24H), 7.07–7.13 (m, 16H), 7.24 (d, J = 3.1 Hz, 2H). ^{13}C NMR (99 MHz, $CDCl_3$, δ): 10.5 (CH_2), 22.3 (CH_3), 30.2 (CH_3), 31.2 (*C*), 32.4 (*C*H), 33.2 (CH_2), 50.9 (CH_2), 124.4 (*C*H), 124.5 (*C*H), 125.0 (*C*H), 135.5 (*C*H), 135.8 (*C*), 135.9 (*C*), 136.0 (*C*), 136.05 (*C*), 136.10 (*C*), 136.3 (*C*), 136.7 (*C*), 137.6 (*C*), 142.0 (*C*). HRMS-ESI (*m*/*z*): [M]$^+$ calcd for $C_{90}H_{132}S_9Si_2$, 1556.7354; found, 1556.7330. mp 115–123 °C.

Disilylated septithiophene derivative bearing ether groups (9).

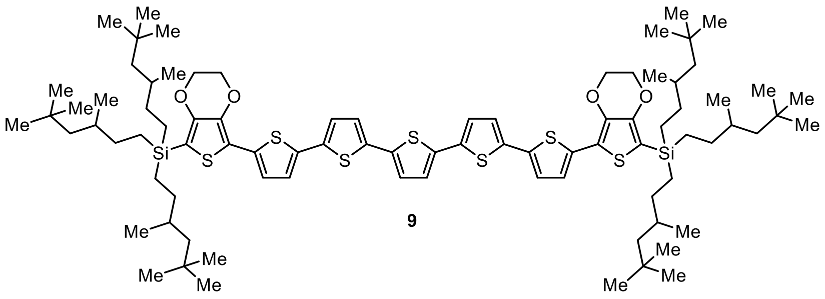

9

The reaction was performed according to example procedure B. The reaction was carried out with 57.1 mg (0.1 mmol) of **4** and 274.0 mg (0.40 mmol) of **2e**. **9** was obtained as a red solid (58.9 mg, 0.039 mmol, 39% yield) after purification by silica-gel column chromatography (SiO_2, CH_2Cl_2/hexane, 0:100–10:90) and recycle preparative GPC.

^{1}H NMR (401 MHz, $CDCl_3$, δ): 0.69–0.94 (m, 84H), 0.98–1.05 (m, 6H), 1.10–1.46 (m, 24H), 4.19–

4.25 (m, 4H), 4.31–4.36 (m, 4H), 7.06–7.10 (m, 8H), 7.13 (d, J = 3.6 Hz, 2H). ^{13}C NMR (99 MHz, $CDCl_3$, δ): 10.0 (CH_2), 22.3 (CH_3), 30.2 (CH_3), 31.2 (C), 32.4 (CH), 33.2 (CH_2), 50.9 (CH_2), 64.5 (CH_2), 65.0 (CH_2), 107.4 (C), 117.0 (C), 123.3 (CH), 124.0 (CH), 124.4 (CH), 124.5 (CH), 134.6 (C), 135.0 (C), 135.5 (C), 136.1 (C), 136.7 (C), 138.4 (C), 147.4 (C). HRMS-APCI (m/z): $[M+H]^+$ calcd for $C_{86}H_{133}O_4S_7Si_2$, 1509.7782; found, 1509.7773. mp 131–134 °C.

Disilylated septithiophene derivative bearing methyl groups (10).

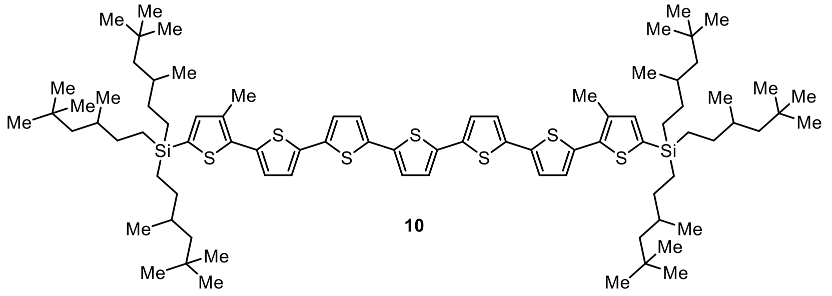

The reaction was performed according to example procedure B. The reaction was carried out with 56.9 mg (0.1 mmol) of **4** and 253.7 mg (0.40 mmol) of **2f**. **10** was obtained as a solid (56.4 mg, 0.040 mmol, 40% yield) after purification by silica-gel column chromatography (SiO_2, CH_2Cl_2/hexane, 0:100–10:90) and recycle preparative GPC.

^{1}H NMR (396 MHz, $CDCl_3$, δ): 0.66–0.96 (m, 84H), 0.98–1.06 (m, 6H), 1.11–1.48 (m, 24H), 2.43 (s, 6H), 6.98 (s, 2H), 7.04–7.15 (m, 10H). ^{13}C NMR (99 MHz, $CDCl_3$, δ): 10.5 (CH_2), 15.6 (CH_3), 22.3 (CH_3), 30.2 (CH_3), 31.2 (C), 32.4 (CH), 33.2 (CH_2), 50.9 (CH_2), 124.2 (CH), 124.3 (CH), 124.47 (CH), 124.53 (CH), 125.9 (CH), 135.3 (C), 135.8 (C), 136.08 (C), 136.11 (C), 136.3 (C), 136.4 (C), 139.3 (C). HRMS-APCI (m/z): $[M+H]^+$ calcd for $C_{84}H_{133}S_7Si_2$, 1421.7985; found, 1421.7971. mp 104–106 °C.

Disilylated oligothiophene derivative bearing acceptor moiety (12).

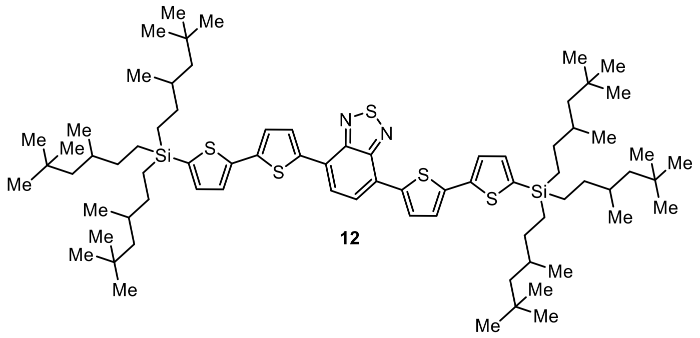

12

The reaction was performed according to example procedure A. The reaction was carried out with 58.5 mg (0.20 mmol) of **11** and 341.4 mg (0.48 mmol) of **2g**. **12** was obtained as a purple solid (114.7 mg, 0.089 mmol, 45% yield) after purification by silica-gel column chromatography (SiO_2, CH_2Cl_2/hexane, 0:100–3:97).

^{1}H NMR (399 MHz, $CDCl_3$, δ): 0.68–0.95 (m, 84H), 0.99–1.06 (m, 6H), 1.13–1.47 (m, 24H), 7.16 (d, *J* = 4.0 Hz, 2H), 7.29 (d, *J* = 4.0 Hz, 2H), 7.36 (d, *J* = 3.2 Hz, 2H), 7.86 (s, 2H), 8.06 (d, *J* = 4.0 Hz, 2H). ^{13}C NMR (100 MHz, $CDCl_3$, δ): 10.5 (CH_2), 22.3 (CH_3), 30.2 (CH_3), 31.2 (*C*), 32.4 (*C*H), 33.2 (CH_2), 50.8 (CH_2), 124.7 (*C*H), 125.2 (*C*H), 125.3 (*C*H), 125.6 (*C*), 128.4 (*C*H), 135.6 (*C*H), 137.9 (*C*), 138.1 (*C*), 139.1 (*C*), 142.2 (*C*), 152.6 (*C*). HRMS-ESI (*m*/*z*): $[M+Na]^+$ calcd for $C_{76}H_{124}N_2NaS_5Si_2$, 1303.7799; found, 1303.7786. mp 70–72 °C.

Disilylated oligothiophene derivative bearing acceptor moiety (14).

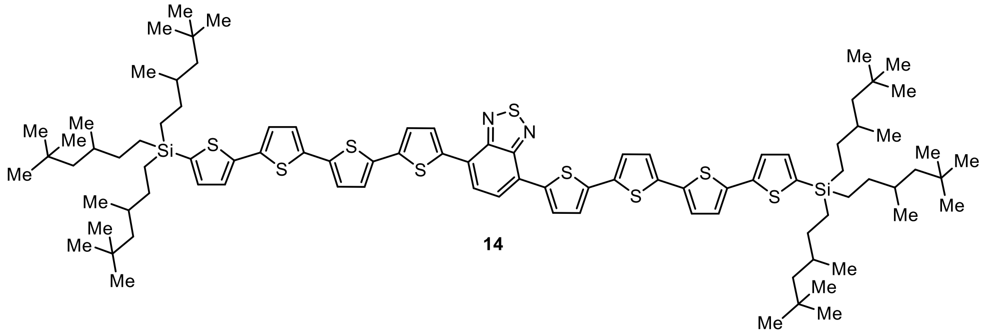

14

The reaction was performed according to example procedure B. The reaction was carried out with 37.5 mg (0.06 mmol) of **13** and 192.7 mg (0.27 mmol) of **2g**. **12** was obtained as a dark purple solid (59.6 mg, 0.037 mmol, 61% yield) after purification by silica-gel column chromatography (SiO_2, CH_2Cl_2/hexane, 0:100–30:70) and recycle preparative GPC. We observed that ^{1}H NMR spectrum changes depending on the concentration of the sample, probably because of its aggregation. We carried out ^{1}H NMR analysis at ca. 3×10^{-3} M and ^{13}C spectrum at ca. 4×10^{-2} M.

^{1}H NMR (401 MHz, $CDCl_3$, δ): 0.68–0.97 (m, 84H), 0.98–1.06 (m, 6H), 1.11–1.46 (m, 24H), 7.05–7.15 (m, 6H), 7.18–7.29 (m, 8H), 7.87 (s, 2H), 8.06 (d, *J* = 4.0 Hz, 2H). ^{13}C NMR (100 MHz, $CDCl_3$,

δ): 10.5 (CH_2), 22.3 (CH_3), 30.2 (CH_3), 31.2 (*C*), 32.4 (*C*H), 33.2 (CH_2), 50.8 (CH_2), 124.4 (*C*H), 124.5 (*C*H), 124.8 (*C*H), 125.1 (*C*H), 125.2 (*C*H), 125.4 (*C*), 128.4 (*C*H), 135.5 (*C*H), 135.8 (*C*), 136.1 (*C*), 136.6 (*C*), 136.7 (*C*), 137.7 (*C*), 138.3 (*C*), 138.6 (*C*), 142.0 (*C*), 152.5 (*C*). HRMS-ESI (*m*/*z*): $[M]^+$ calcd for $C_{92}H_{132}N_2S_9Si_2$, 1608.7415; found, 1608.7377. mp 128–130 °C.

Disilylated terthiophene (15).

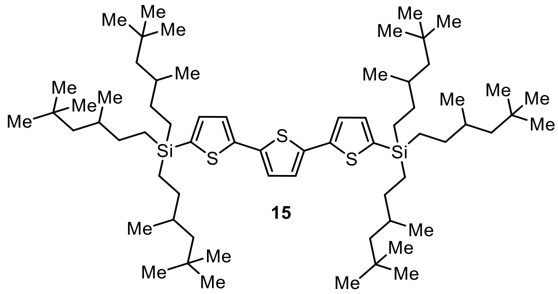

The reaction was performed according to the example procedure A. The reaction was carried out with 48.0 mg (0.2 mmol) of 2,5-dibromothiophene and 301.4 mg (0.49 mmol) of **2g**. **15** was obtained as a yellow oil (162.3 mg, 0.15 mmol, 77% yield) after purification by silica-gel column chromatography (SiO_2, CH_2Cl_2/hexane, 0:100−1:99) and recycle preparative GPC.

^{1}H NMR (392 MHz, $CDCl_3$, δ): 0.72–0.94 (m, 84H), 0.97–1.05 (m, 6H), 1.12–1.46 (m, 24H), 7.09 (s, 2H), 7.11 (d, *J* = 3.5 Hz, 2H), 7.23 (d, *J* = 3.1 Hz, 2H). ^{13}C NMR (99 MHz, $CDCl_3$, δ): 10.5 (CH_2), 22.3 (CH_3), 30.2 (CH_3), 31.2 (*C*), 32.4 (*C*H), 33.2 (CH_2), 50.9 (CH_2), 124.5 (*C*H), 124.9 (*C*H), 135.5 (*C*H), 136.4 (*C*), 137.3 (*C*), 142.3 (*C*). HRMS-APCI (*m*/*z*): $[M+H]^+$ calcd for $C_{66}H_{121}S_3Si_2$, 1065.8164; found, 1065.8147.

4. Example procedure for cross-coupling reactions in solution

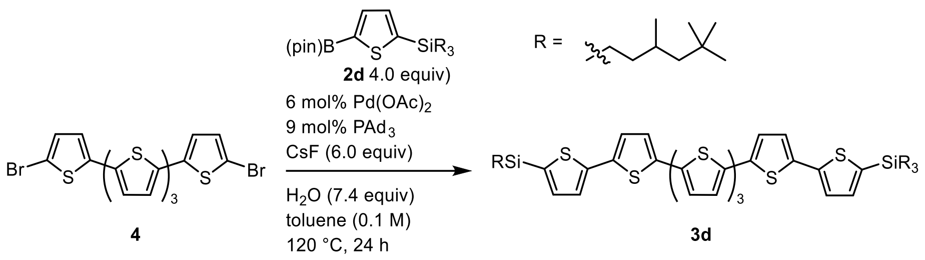

4 (57.0 mg, 0.10 mmol), **2d** (253.7 mg, 0.41 mmol), $Pd(OAc)_2$ (1.5 mg, 0.006 mmol), and PAd_3 (3.9 mg, 0.009 mmol), CsF (97.7 mg, 0.64 mmol) were placed in an oven-dried reaction vial. After the vial was sealed with a screw cap containing a Teflon-coated rubber septum, the vial was connected to a vacuum/nitrogen manifold through a needle. It was evacuated and then backfilled with nitrogen. This cycle was repeated three times. After toluene (1.0 mL) was added into the vial through the rubber septum. Then H_2O (13 μL, 7.4 equiv) was added to the mixture at 120 °C using an oil bath. After 24 hours, the mixture was filtration with CH_2Cl_2 to remove the starting material and inorganic salts. The crude mixture was then purified by flash column chromatography (SiO_2, typically DCM/hexane, typically 0-10:90) and recycling preparative GPC to give the corresponding coupling product **3d** as an orange solid (65.4 mg, 0.050 mmol, 50% yield).

5. Example procedure for desilylation

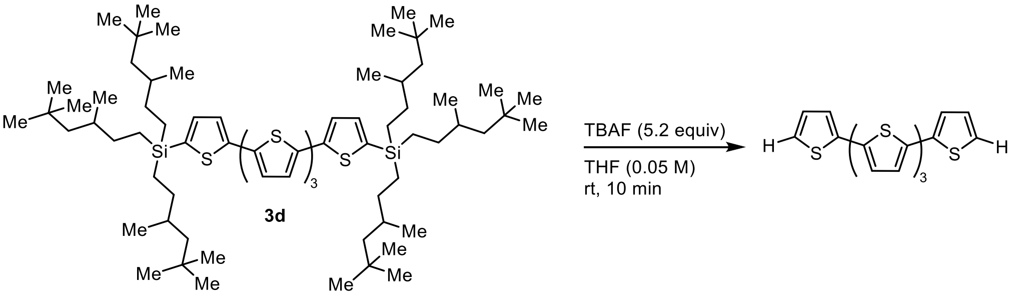

In a 50 mL one-necked round-bottomed flask, **3d** (182.5 mg, 0.15 mmol) was dissolved in THF (2.1 ml). To the reaction mixture, tetrabutylammonium fluoride (TBAF) (*ca*. 1.0 M in THF) (0.77 ml, 5.2 equiv) was added. After stirring for 10 min, the solvent was removed by evaporation, and the remaining solid was washed by Et_2O and MeOH to afford nonsubstituted quinquethiophene as a yellow solid (54.5 mg, 0.13 mmol, 89% yield). ^{1}H NMR was in agreement with the literature.[4] ^{13}C NMR peaks were barely detected because of the low solubility of this compound. ^{1}H NMR (396 MHz, $CDCl_3$, δ): 7.02–7.06 (m, 2H), 7.09 (s, 6H), 7.18–7.27 (m, 4H). HRMS-EI (*m*/*z*): $[M]^+$ calcd for $C_{20}H_{12}S_5$, 411.9543; found, 411.9538. Anal. Calcd for $C_{20}H_{12}S_5$: C, 58.22; H, 2.93. Found: C, 57.66; H, 2.82. mp 203–204 °C.

Nonsubstituted septithiophene.

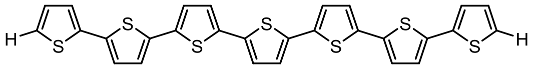

The reaction was carried out with 311.5 mg (0.24 mmol) of **5**, 1.25 ml (1.25 mmol) of TBAF (*ca* 1 M in THF), and 2.0 ml of THF. Nonsubstituted septithiophene was obtained as a bright orange solid (98.7 mg, 0.17 mmol, 72% yield). ^{1}H and ^{13}C NMR peaks were barely detected because of the low solubility of this compound. HRMS-EI (*m*/*z*): $[M]^+$ calcd for $C_{28}H_{16}S_7$, 575.9297; found, 575.9295. mp >300 °C.

Nonsubstituted novithiophene (8).

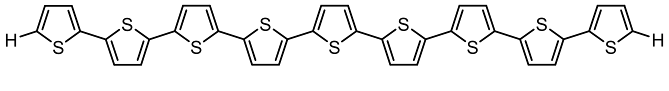

8

The reaction was carried out with 254.3 mg (0.16 mmol) of **7**, 0.85 ml (0.85 mmol) of TBAF (*ca* 1 M in THF), and 0.60 ml of THF. **8** was obtained as a red solid (94.1 mg, 0.13 mmol, 79% yield). ^{1}H and ^{13}C NMR peaks were barely detected because of the low solubility of this compound. HRMS-ESI (*m*/*z*): $[M+H]^+$ calcd for $C_{36}H_{21}S_9$, 740.9124; found, 740.9117. mp >300 °C.

Nonsubstituted oligothiophene derivative bearing accepter moiety.

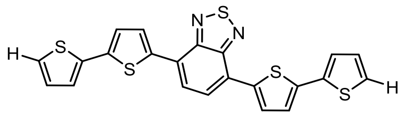

The reaction was carried out with 91.6 mg (0.07 mmol) of **12,** 0.37 ml (0.37 mmol) of TBAF (*ca* 1 M in THF), and 0.7 ml of THF. Nonsubstituted oligothiophene derivative bearing acceptor moiety was obtained as a purple solid (27.3 mg, 0.059 mmol, 82% yield). ^{1}H NMR was in agreement with the literature.[5]

^{1}H NMR (396 MHz, $CDCl_3$, δ): 7.07 (t, *J* = 3.6 Hz, 2H), 7.20–7.39 (m, 6H), 7.87 (s, 2H), 8.06 (d, *J* = 4.0 Hz, 2H). ^{13}C NMR (99 MHz, $CDCl_3$, δ): 124.2 (*C*H), 124.7 (*C*H), 125.0 (*C*H), 125.4 (*C*H), 125.7 (*C*), 128.2 (*C*H), 128.4 (*C*H), 137.4 (*C*), 138.2 (*C*), 139.1 (*C*), 152.6 (*C*). HRMS-EI (*m*/*z*): $[M]^+$ calcd for $C_{22}H_{12}N_2S_5$, 463.9604; found, 463.9596. mp 203–204 °C.

6. Example procedure for bromination

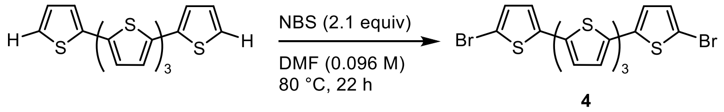

N-Bromosuccinimide (NBS) (178.6mg, 1.00 mmol) and nonsubstituted quinquethiophene (200.6 mg, 0.48 mmol) were dissolved in DMF (5.0 mL) at room temperature under nitrogen atmosphere. The mixture was stirred for 22 h at 80 °C and then cooled to room temperature. MeOH (10 mL) was added to the mixture, and the crude orange solid was precipitated, filtered, and washed with CH_2Cl_2 and MeOH to afford dibromominated quinquethiophene (**4**) as an orange solid (201.0 mg, 0.35 mmol, 73% yield). HRMS-EI (*m*/*z*): $[M]^+$ calcd for $C_{20}H_{10}Br_2S_5$, 567.7753; found, 567.7743. mp 292–294 °C.

Dibromominated septithiophene (6).

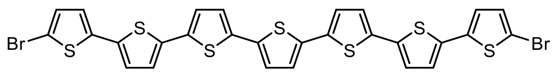

The reaction was carried out with 259.8 mg (0.45 mmol) of nonsubstituted septithiophene. Dibrominated septithiophene (**6**) was obtained as a red solid (205.3 mg, 0.28 mmol, 62% yield). ^1H and ^{13}C NMR peaks were barely detected because of the low solubility of **6**. HRMS-EI (*m*/*z*): $[M]^+$ calcd for $C_{28}H_{14}Br_2S_7$, 731.7507; found, 731.7493. mp >300 °C.

Preparation of dibrominated oligothiophene derivative bearing acceptor moiety (13).

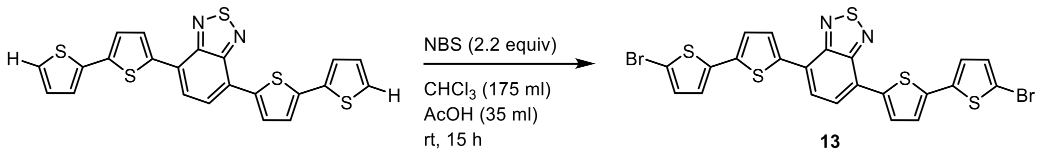

The reaction was performed according to the literature procedure.[5] In a 500 ml round-bottom flask, nonsubstituted oligothiophene derivative bearing acceptor moiety (163.2 mg, 0.35 mmol) was dissolved in a mixed solvent of $CHCl_3$ (175 mL) and acetic acid (35 mL), and the flask was covered with aluminum foil. NBS (136.5 mg, 2.2 mmol) was added in portions, and the reaction mixture was stirred for 15 h at room temperature. Then, methanol (50 mL) was added, and the mixture was filtered and washed with water, methanol, and CH_2Cl_2. The precipitate was collected as a dark blue solid (170.1 mg, 0.27 mmol, 78% yield). ^1H and ^{13}C NMR peaks were barely detected because of the low solubility of **13**. HRMS-ESI (*m*/*z*): $[M]^-$ calcd for $C_{22}H_{10}N_2Br_2S_5$, 619.7820; found, 619.7831. mp 246–248 °C.

7. Control experiments

Investugations of the effect of ball milling

To clearfy the effect of ball-milling process, we conducted some additional control experiments.

The reaction between **1** and **2d** was carried out in a jar without milling while applying a heat gun, resulted in no product formation. In addition, we also checked the reaction in a test tube with stirring bar (1020 rpm) and **3d** was obtained in 33% yield, which was much lower than that of the ball-milling conditions (79%). These results suggest that the ball milling process is essential to achieve the high reaction efficiency.

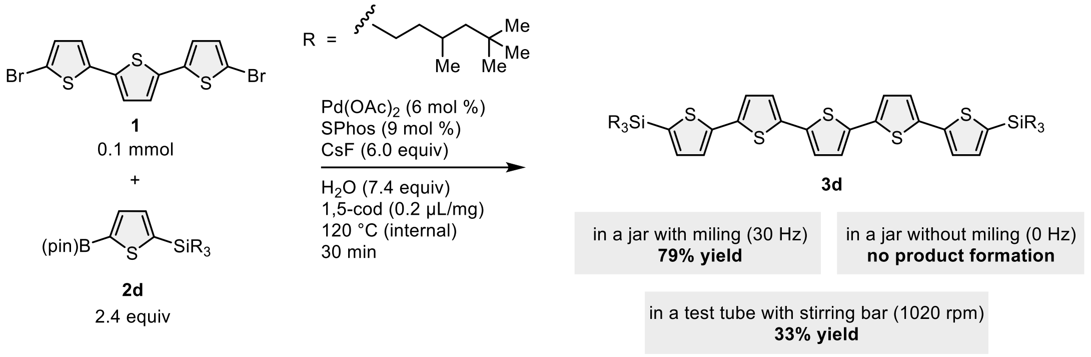

The use of trimethylsilyl group (TMS) as a solubilizing group

The cross-coupling reaction between **1** and 2-boryl thiophene bearing a TMS group was investigated. The reaction proceeded to give the corresponding product, however, the solubility of the product is very low and the purification by column chromatography was sluggish. The product was obtained in only 12% yield. This result suggests that the use of a silyl group with long alkyl chains as a solubilizing group is essential for this strategy.

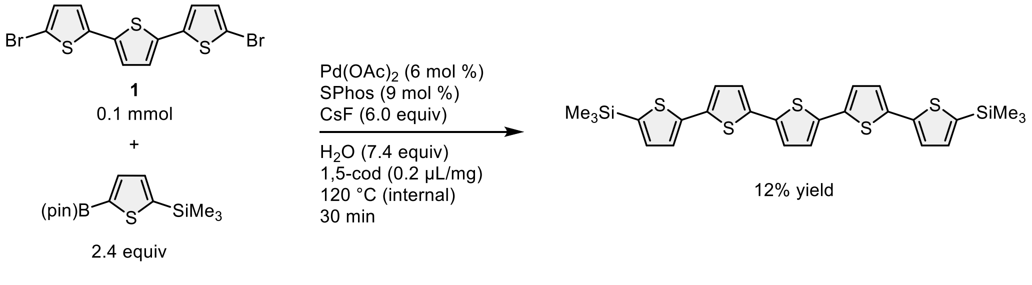

8. Thermography Observation for Reaction Temperature

The temperature inside the milling jar after the solid-state coupling reactions was confirmed by observation with a thermography camera immediately after opening the milling jar (Figures S6 and S7). When the preset temperature of the heat gun was 250 °C for a 1.5 mL stainless jar and 5 mm ball (30 Hz, 30 min), the internal temperature was determined to be 122.8 °C (Figure S6). On the other hand, when a 5 mL stainless jar and 10 mm ball (25 Hz, 90 min) were used, the internal temperature was determined to be 121.7 °C (Figure S7).

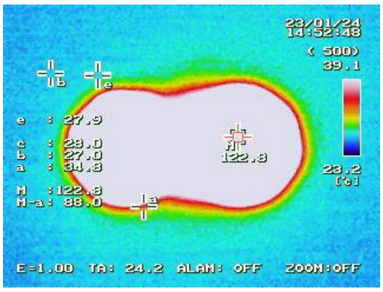

Figure S6. Thermography image inside the milling jar (1.5 ml) after grinding for 30 min at 30 Hz.

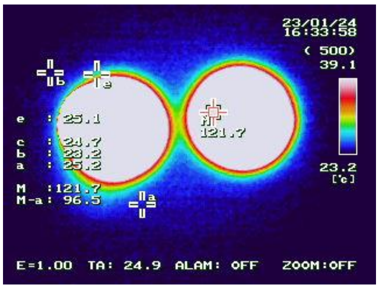

Figure S7. Thermography image inside the milling jar (5.0 ml) after grinding for 90 min at 25 Hz.

9. Optical properties

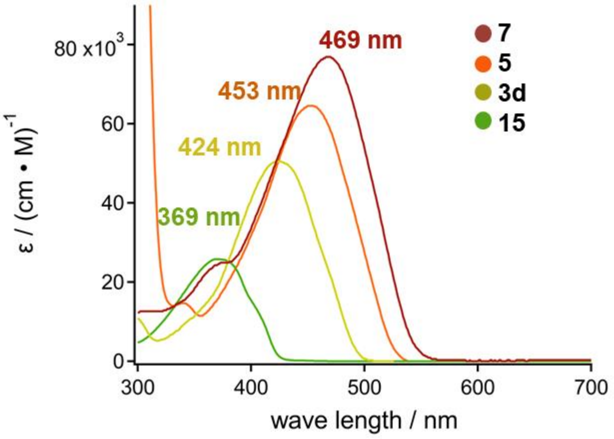

Figure S8. Absorption spectra of $CHCl_3$ solution of **3d** (brown line), **5** (orange line), **7** (yellow line), **15** (green line).

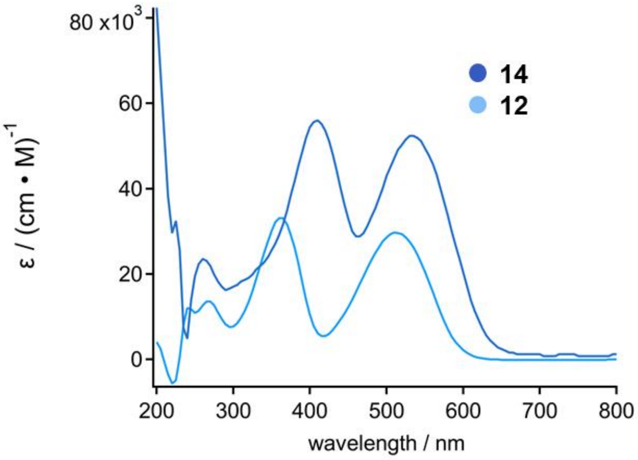

Figure S9. Absorption spectra of $CHCl_3$ solution of **14** (blue line), **12**(light blue line).

10. References

1. Raymond, J. E.; Bhaskar, A.; Goodson, T.; Makiuchi, N.; Ogawa, K.; Kobuke, Y. *J. Am. Chem. Soc.* **2008**, *130*, 17212.
2. Lu, B.; Falck, J. R. *Angew. Chem. Int. Ed.* **2008**, *47*, 7508.
3. Karmel, C.; Chen, Z.; Hartwig, J. F. *J. Am. Chem. Soc.* **2019**, *141*, 7063.
4. Martínez, M. M.; Peña-López, M.; Sestelo, J. P.; Sarandeses, L. A. *Org. Biomol. Chem*. **2012**, *10*, 3892.
5. Zhou, E.; Yamakawa, S.; Zhang, Y.; Tajima, K.; Yang, C.; Hashimoto, K. *J. Mater. Chem.* **2009**, *19*, 7730.

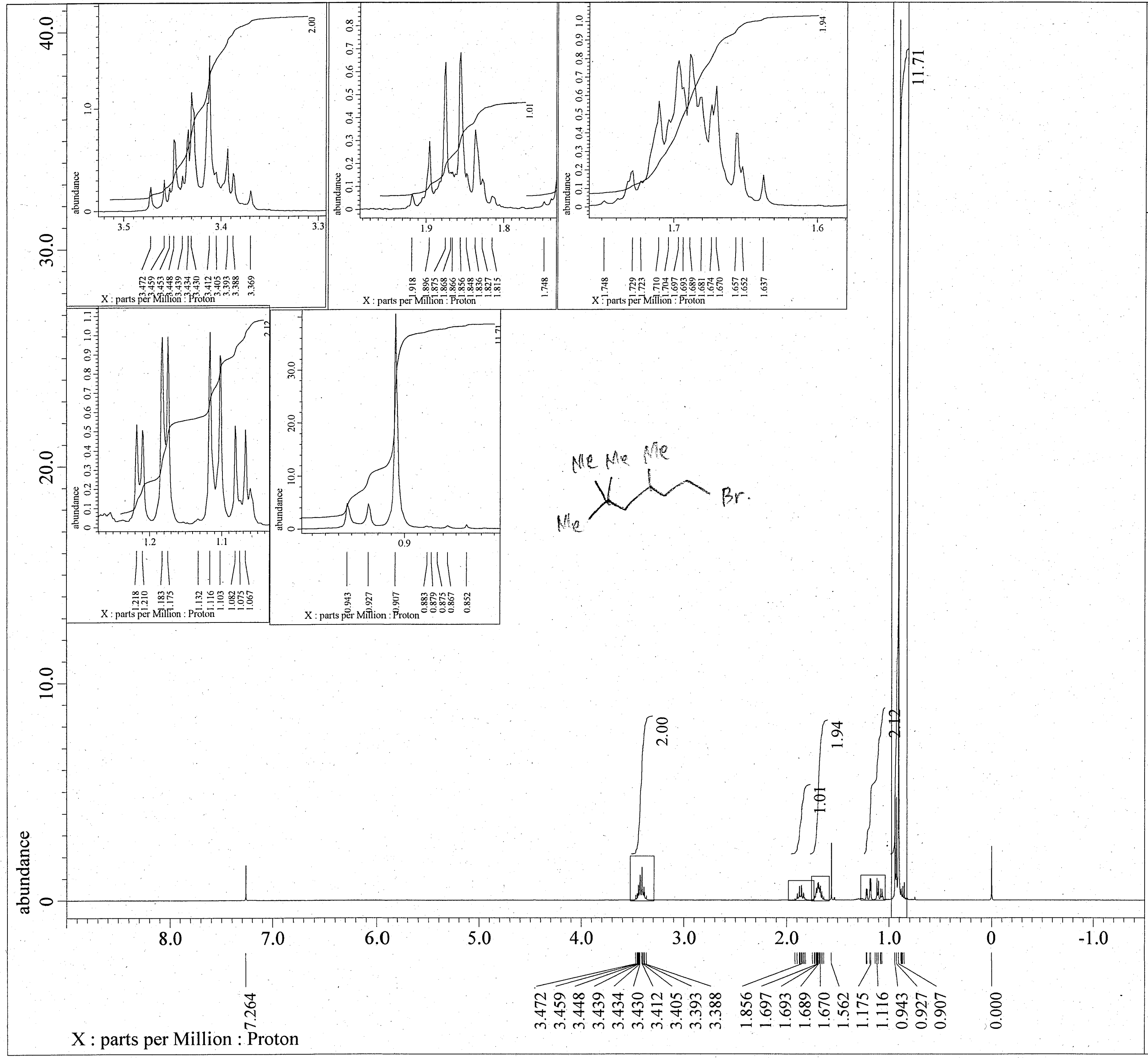


```
---- PROCESSING PARAMETERS ----
dc_balance( 0, FALSE )
sexp( 0.2[Hz], 0.0[s] )
trapezoid( 0[%], 0[%], 80[%], 100[%] )
zerofill( 1 )
fft( 1, TRUE, TRUE )
machinephase
ppm

Derived from: KND_580_pure_Proton-1-1.jdf
```

```
Filename            = KND_580_pure_Proton-1-2.j
Author              = element
Experiment          = proton.jxp
Sample_Id           = KND_580_pure
Solvent             = CHLOROFORM-D
Actual_Start_Time   =  5-OCT-2022 12:45:02
Revision_Time       =  7-SEP-2023 10:31:12

Comment             = single_pulse
Data_Format         = 1D COMPLEX
Dim_Size            = 13107
X_Domain            = Proton
Dim_Title           = Proton
Dim_Units           = [ppm]
Dimensions          = X
Site                = JNM-ECS400
Spectrometer        = DELTA2_NMR

Field_Strength      = 9.37221[T] (400[MHz])
X_Acq_Duration      = 2.1889024[s]
X_Domain            = 1H
X_Freq              = 399.03472754[MHz]
X_Offset            = 5.0[ppm]
X_Points            = 16384
X_Prescans          = 1
X_Resolution        = 0.45684997[Hz]
X_Sweep             = 7.48502994[kHz]
X_Sweep_Clipped     = 5.98802395[kHz]
Irr_Domain          = Proton
Irr_Freq            = 399.03472754[MHz]
Irr_Offset          = 5.0[ppm]
Tri_Domain          = Proton
Tri_Freq            = 399.03472754[MHz]
Tri_Offset          = 5.0[ppm]
Clipped             = FALSE
Scans               = 8
Total_Scans         = 8

Relaxation_Delay    = 5[s]
Recvr_Gain          = 36
Temp_Get            = 17.9[dC]
X_90_Width          = 6.6[us]
X_Acq_Time          = 2.1889024[s]
X_Angle             = 45[deg]
X_Atn               = 1[dB]
X_Pulse             = 3.3[us]
Irr_Mode            = Off
Tri_Mode            = Off
Dante_Presat        = FALSE
Initial_Wait        = 1[s]
Repetition_Time     = 7.1889024[s]
```

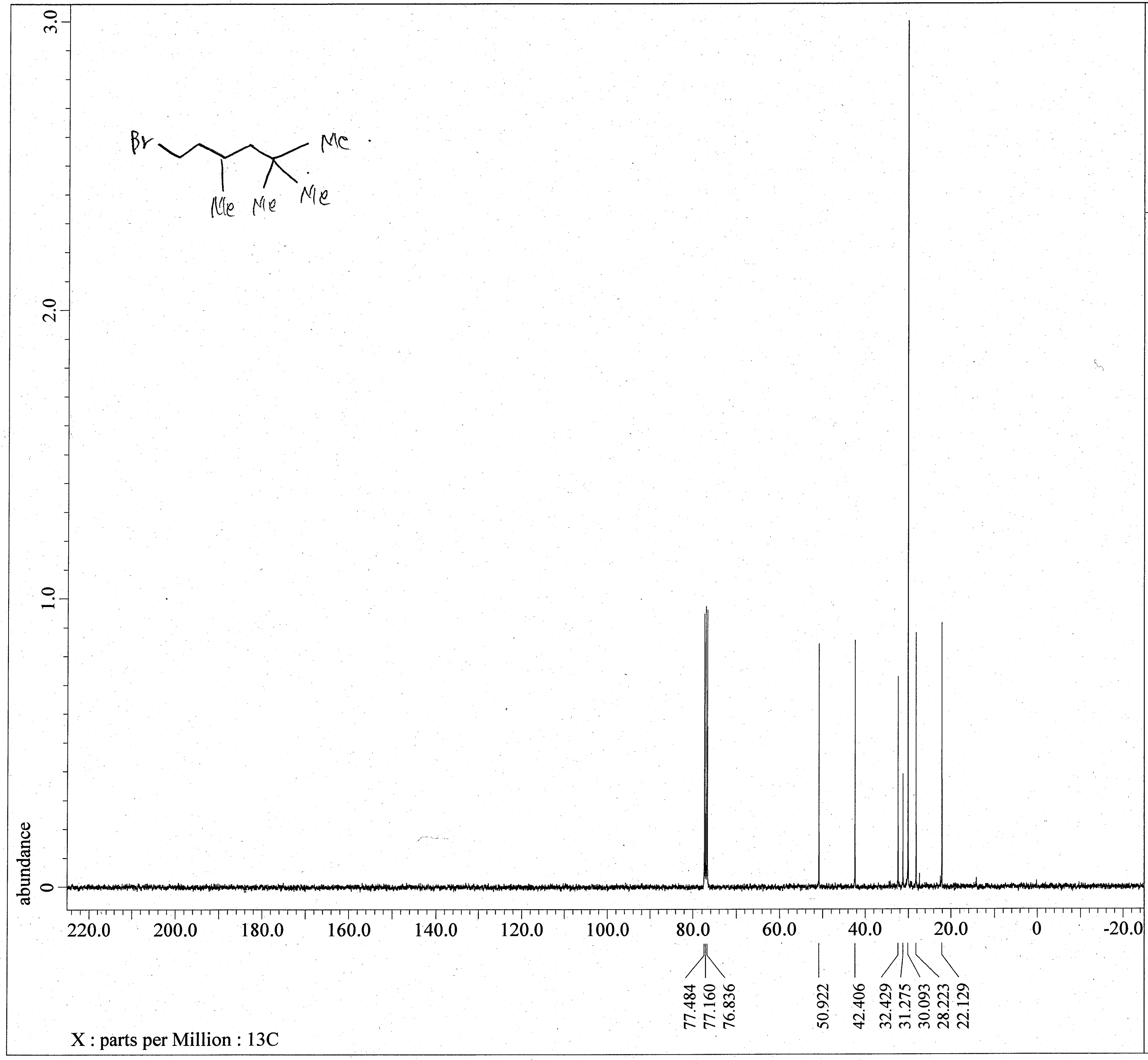


```
---- PROCESSING PARAMETERS ----
dc_balance( 0, FALSE )
sexp( 2.0[Hz], 0.0[s] )
trapezoid3( 0[%], 80[%], 100[%] )
zerofill( 1 )
fft( 1, TRUE, TRUE )
machinephase
ppm

Derived from: KND_580_13C-1.jdf
```

```
Filename             = KND_580_13C-2.jdf
Author               = element
Experiment           = single_pulse_dec
Sample_Id            = 1
Solvent              = CHLOROFORM-D
Actual_Start_Time    =  5-OCT-2022 18:47:33
Revision_Time        =  8-OCT-2022 15:05:24

Comment              = single pulse decoupled ga
Data_Format          = 1D COMPLEX
Dim_Size             = 26214
X_Domain             = 13C
Dim_Title            = 13C
Dim_Units            = [ppm]
Dimensions           = X
Site                 = ECS 400
Spectrometer         = JNM-ECS400

Field_Strength       = 9.20197068[T] (390[MHz])
X_Acq_Duration       = 1.06430464[s]
X_Domain             = 13C
X_Freq               = 98.51479726[MHz]
X_Offset             = 100[ppm]
X_Points             = 32768
X_Prescans           = 4
X_Resolution         = 0.93958061[Hz]
X_Sweep              = 30.78817734[kHz]
Irr_Domain           = 1H
Irr_Freq             = 391.78655441[MHz]
Irr_Offset           = 5[ppm]
Clipped              = FALSE
Scans                = 200
Total_Scans          = 200

Relaxation_Delay     = 2[s]
Recvr_Gain           = 60
Temp_Get             = 19[dC]
X_90_Width           = 8.7[us]
X_Acq_Time           = 1.06430464[s]
X_Angle              = 30[deg]
X_Atn                = 4.9[dB]
X_Pulse              = 2.9[us]
Irr_Atn_Dec          = 22.45[dB]
Irr_Atn_Noe          = 22.45[dB]
Irr_Noise            = WALTZ
Decoupling           = TRUE
Initial_Wait         = 1[s]
Noe                  = TRUE
Noe_Time             = 2[s]
Repetition_Time      = 3.06430464[s]
```

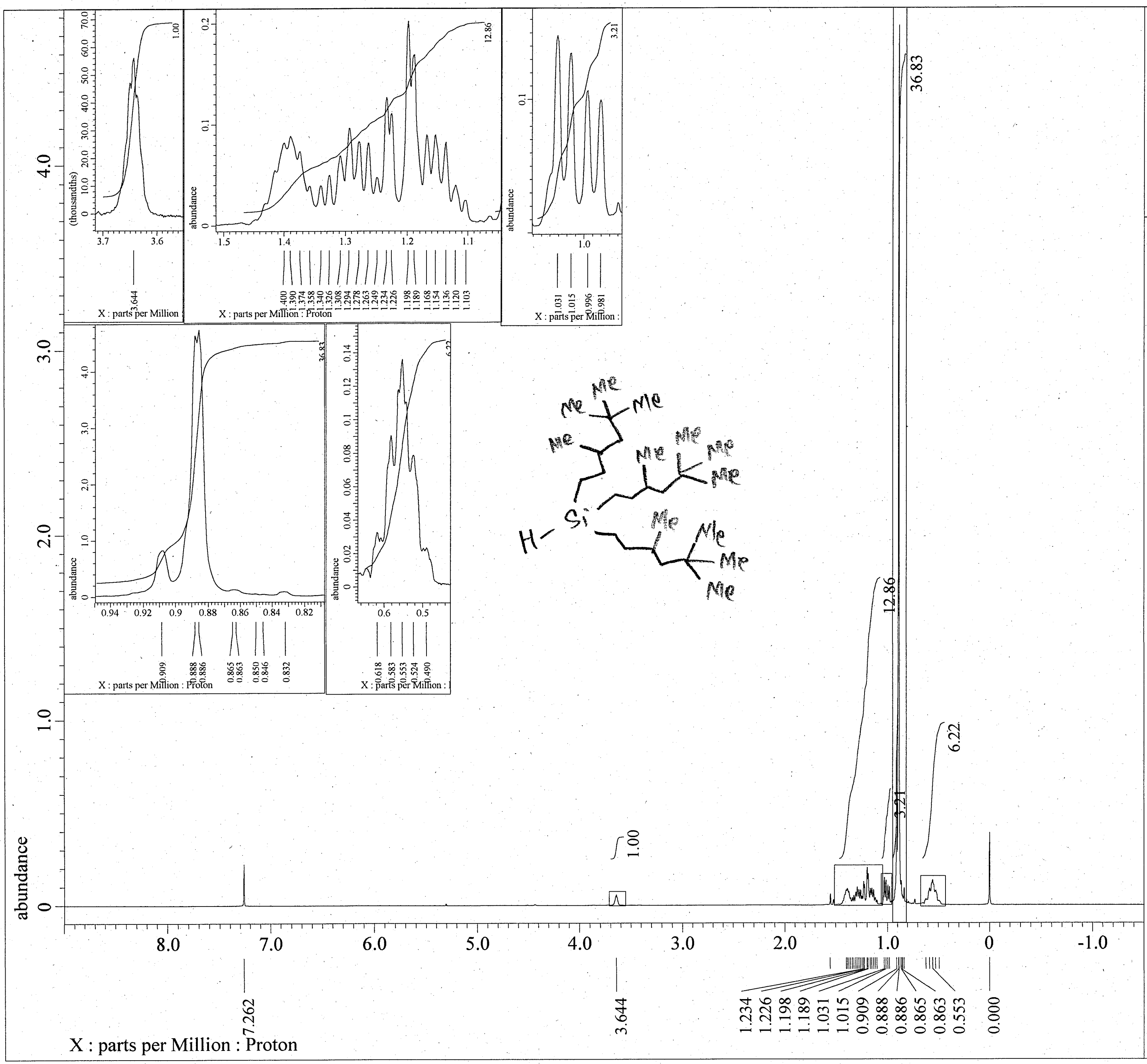

---- PROCESSING PARAMETERS ----
sexp(0.2[Hz], 0.0[s])
trapezoid(0[%], 0[%], 80[%], 100[%])
zerofill(1)
fft(1, TRUE, TRUE)
machinephase
ppm

Derived from: KND_661_pure2_Proton-1-1.jdf

Filename	= KND_661_pure2_Proton-
Author	= element
Experiment	= proton_auto.jxp
Sample_Id	= KND_661_pure2
Solvent	= CHLOROFORM-D
Actual_Start_Time	= 7-NOV-2022 18:06:48
Revision_Time	= 7-SEP-2023 15:43:33
Comment	= single_pulse
Data_Format	= 1D COMPLEX
Dim_Size	= 13107
X_Domain	= Proton
Dim_Title	= Proton
Dim_Units	= [ppm]
Dimensions	= X
Spectrometer	= DELTA2_NMR
Field_Strength	= 9.2982153[T] (400[MHz
X_Acq_Duration	= 2.20725248[s]
X_Domain	= Proton
X_Freq	= 395.88430144[MHz]
X_Offset	= 5[ppm]
X_Points	= 16384
X_Prescans	= 1
X_Resolution	= 0.45305193[Hz]
X_Sweep	= 7.42280285[kHz]
X_Sweep_Clipped	= 5.93824228[kHz]
Irr_Domain	= Proton
Irr_Freq	= 395.88430144[MHz]
Irr_Offset	= 5[ppm]
Tri_Domain	= Proton
Tri_Freq	= 395.88430144[MHz]
Tri_Offset	= 5[ppm]
Blanking	= 2[us]
Clipped	= TRUE
Scans	= 8
Total_Scans	= 8
Relaxation_Delay	= 5[s]
Recvr_Gain	= 56
Temp_Get	= 17.5[dC]
X_90_Width	= 9.5[us]
X_Acq_Time	= 2.20725248[s]
X_Angle	= 45[deg]
X_Atn	= 3.4[dB]
X_Pulse	= 4.75[us]
Irr_Mode	= Off
Tri_Mode	= Off
Dante_Loop	= 500
Dante_Presat	= FALSE
Decimation_Rate	= 0
Experiment_Path	= C:\Program Files\JEOL
Initial_Wait	= 1[s]
Phase	= {0, 90, 270, 180, 180
Presat_Time	= 5[s]

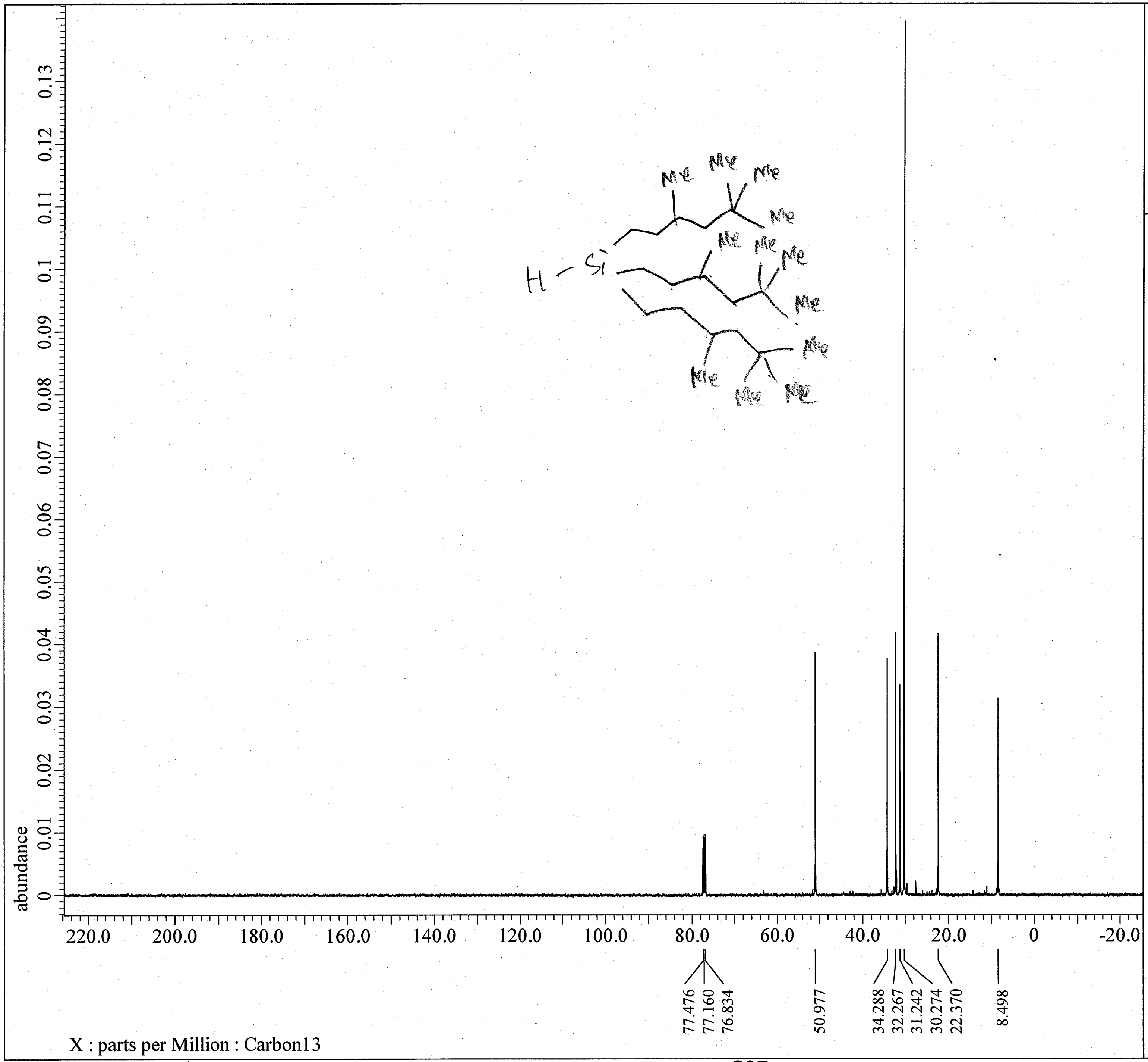


```
---- PROCESSING PARAMETERS ----
sexp( 2.0[Hz], 0.0[s] )
trapezoid( 0[%], 0[%], 80[%], 100[%] )
zerofill( 1 )
fft( 1, TRUE, TRUE )
machinephase
ppm

Derived from: KND_661_13C_Carbon-1-1.jdf
```

```
Filename                    = KND_661_13C_Carbon
Author                      = element
Experiment                  = carbon_auto.jxp
Sample_Id                   = KND_661_13C
Solvent                     = CHLOROFORM-D
Actual_Start_Time           =  7-NOV-2022 18:19:
Revision_Time               = 11-NOV-2022 20:04:

Comment                     = single pulse decou
Data_Format                 = 1D COMPLEX
Dim_Size                    = 26214
X_Domain                    = Carbon13
Dim_Title                   = Carbon13
Dim_Units                   = [ppm]
Dimensions                  = X
Spectrometer                = DELTA2_NMR

Field_Strength              = 9.2982153[T] (400[
X_Acq_Duration              = 1.048576[s]
X_Domain                    = Carbon13
X_Freq                      = 99.54517646[MHz]
X_Offset                    = 100[ppm]
X_Points                    = 32768
X_Prescans                  = 4
X_Resolution                = 0.95367432[Hz]
X_Sweep                     = 31.25[kHz]
X_Sweep_Clipped             = 25[kHz]
Irr_Domain                  = Proton
Irr_Freq                    = 395.88430144[MHz]
Irr_Offset                  = 5[ppm]
Blanking                    = 5[us]
Clipped                     = FALSE
Scans                       = 100
Total_Scans                 = 100

Relaxation_Delay            = 2[s]
Recvr_Gain                  = 50
Temp_Get                    = 17.3[dC]
X_90_Width                  = 9.65[us]
X_Acq_Time                  = 1.048576[s]
X_Angle                     = 30[deg]
X_Atn                       = 8[dB]
X_Pulse                     = 3.21666667[us]
Irr_Atn_Dec                 = 25.059[dB]
Irr_Atn_Dec_Calc            = 25.059[dB]
Irr_Atn_Dec_Default_Calc    = 25.059[dB]
Irr_Atn_Noe                 = 25.059[dB]
Irr_Dec_Bandwidth_Hz        = 4.7826087[kHz]
Irr_Dec_Bandwidth_Ppm       = 12.08082432[ppm]
Irr_Dec_Freq                = 395.88430144[MHz]
Irr_Dec_Merit_Factor        = 2.2
Irr_Decoupling              = TRUE
Irr_Noe                     = TRUE
Irr_Noise                   = WALTZ
Irr_Offset_Default          = 5[ppm]
```

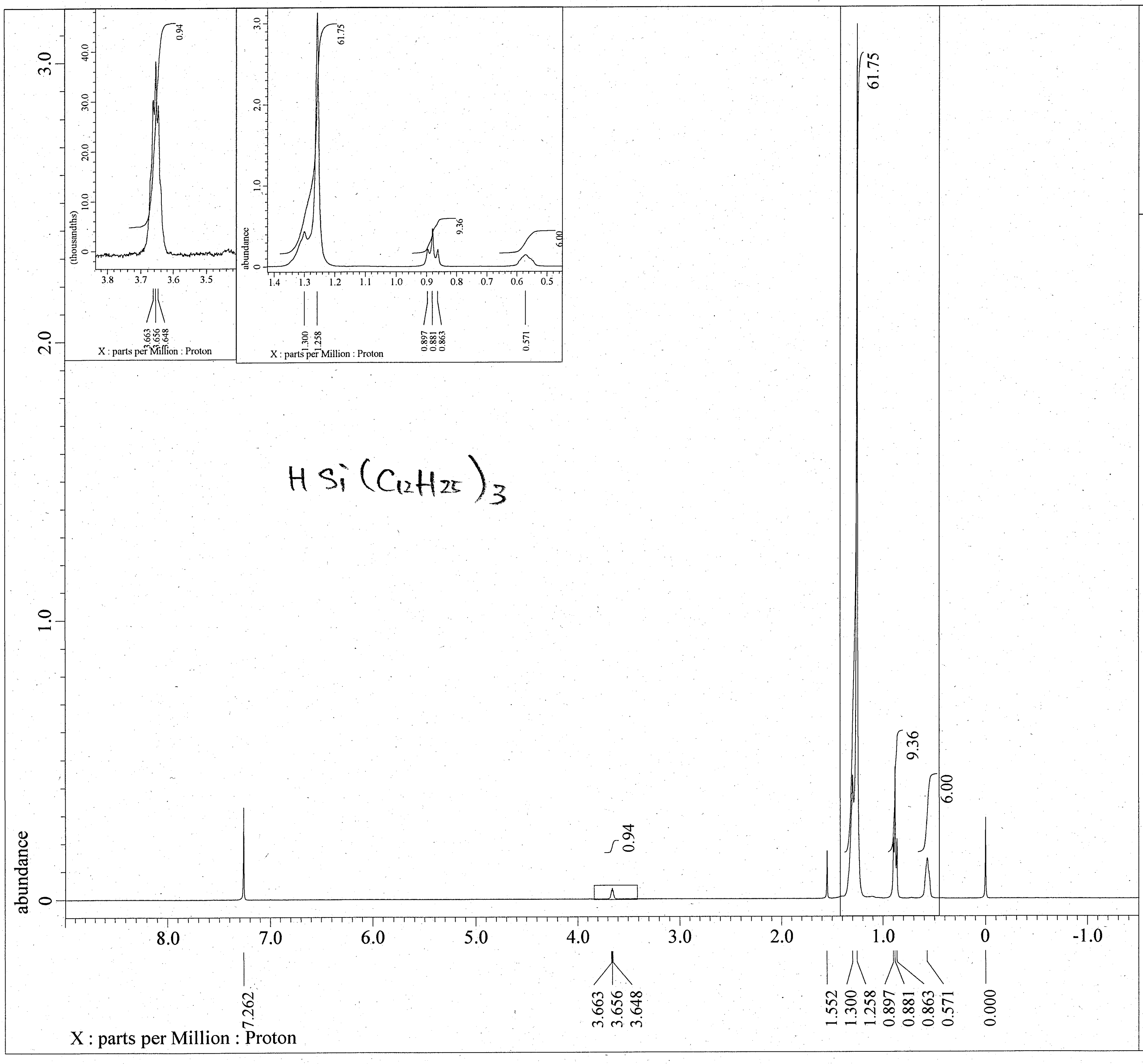


```
---- PROCESSING PARAMETERS ----
sexp( 0.2[Hz], 0.0[s] )
trapezoid( 0[%], 0[%], 80[%], 100[%] )
zerofill( 1 )
fft( 1, TRUE, TRUE )
machinephase
ppm

Derived from: KND_908_pure_Proton-1-1.jdf
```

```
Filename             = KND_908_pure_Proton-1
Author               = element
Experiment           = proton_auto.jxp
Sample_Id            = KND_908_pure
Solvent              = CHLOROFORM-D
Actual_Start_Time    = 19-JAN-2023 23:14:08
Revision_Time        =  7-SEP-2023 15:50:19

Comment              = single_pulse
Data_Format          = 1D COMPLEX
Dim_Size             = 13107
X_Domain             = Proton
Dim_Title            = Proton
Dim_Units            = [ppm]
Dimensions           = X
Spectrometer         = DELTA2_NMR

Field_Strength       = 9.2982153[T] (400[MHz
X_Acq_Duration       = 2.20725248[s]
X_Domain             = Proton
X_Freq               = 395.88430144[MHz]
X_Offset             = 5[ppm]
X_Points             = 16384
X_Prescans           = 1
X_Resolution         = 0.45305193[Hz]
X_Sweep              = 7.42280285[kHz]
X_Sweep_Clipped      = 5.93824228[kHz]
Irr_Domain           = Proton
Irr_Freq             = 395.88430144[MHz]
Irr_Offset           = 5[ppm]
Tri_Domain           = Proton
Tri_Freq             = 395.88430144[MHz]
Tri_Offset           = 5[ppm]
Blanking             = 2[us]
Clipped              = TRUE
Scans                = 8
Total_Scans          = 8

Relaxation_Delay     = 5[s]
Recvr_Gain           = 56
Temp_Get             = 18.4[dC]
X_90_Width           = 9.5[us]
X_Acq_Time           = 2.20725248[s]
X_Angle              = 45[deg]
X_Atn                = 3.4[dB]
X_Pulse              = 4.75[us]
Irr_Mode             = Off
Tri_Mode             = Off
Dante_Loop           = 500
Dante_Presat         = FALSE
Decimation_Rate      = 0
Experiment_Path      = C:\Program Files\JEOL
Initial_Wait         = 1[s]
Phase                = {0, 90, 270, 180, 180
Presat_Time          = 5[s]
```

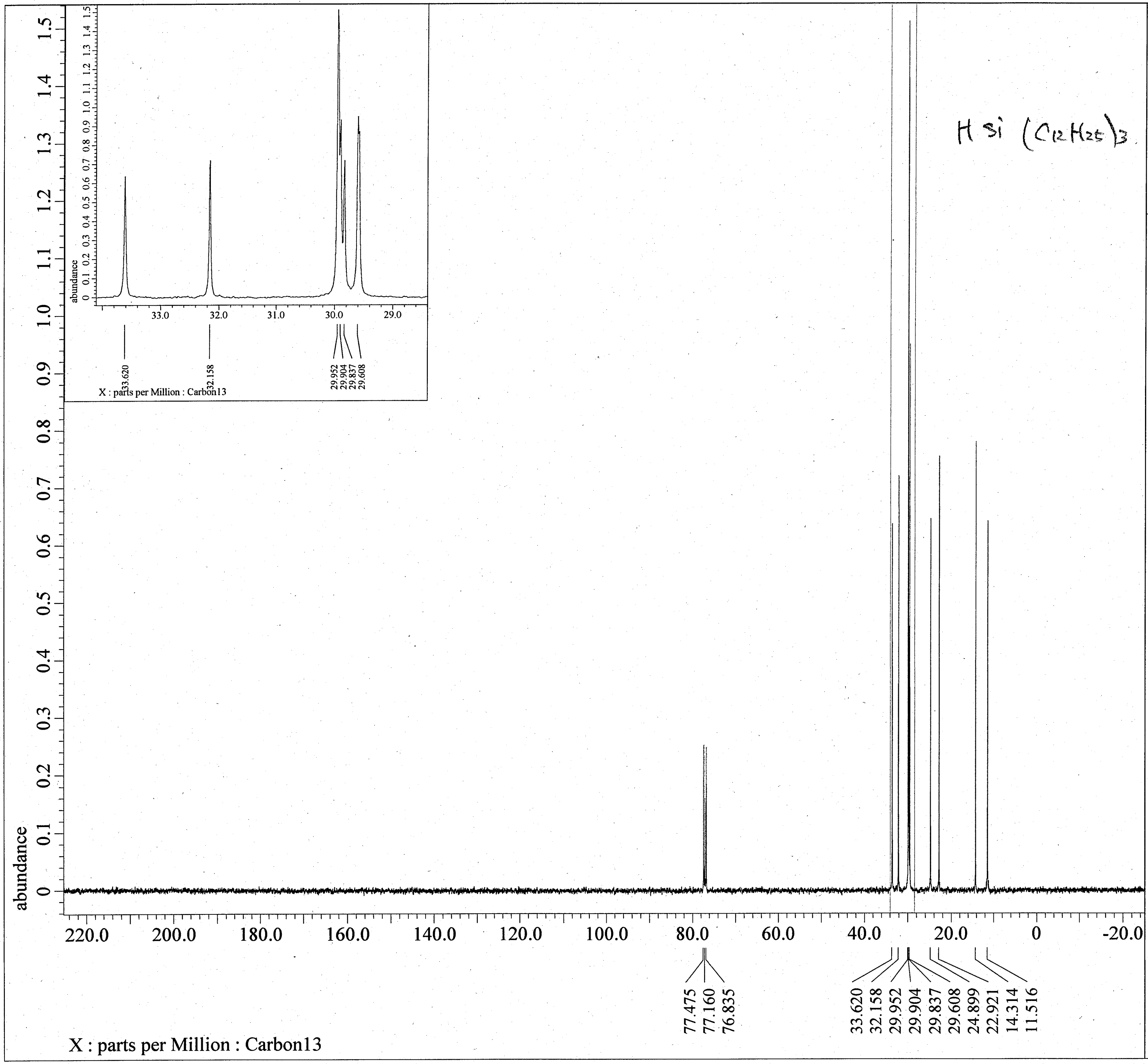


```
---- PROCESSING PARAMETERS ----
dc_balance( 0, FALSE )
sexp( 2.0[Hz], 0.0[s] )
trapezoid( 0[%], 0[%], 80[%], 100[%] )
zerofill( 1 )
fft( 1, TRUE, TRUE )
machinephase
ppm

Derived from: KND_908_13C_Carbon-1-1.jdf

Filename            = KND_908_13C_Carbon-1-2.jd
Author              = element
Experiment          = carbon.jxp
Sample_Id           = KND_908_13C
Solvent             = CHLOROFORM-D
Actual_Start_Time   = 20-JAN-2023 00:26:35
Revision_Time       = 20-JAN-2023 23:17:47

Comment             = single pulse decoupled ga
Data_Format         = 1D COMPLEX
Dim_Size            = 26214
X_Domain            = Carbon
Dim_Title           = Carbon13
Dim_Units           = [ppm]
Dimensions          = X
Site                = JNM-ECS400
Spectrometer        = DELTA2_NMR

Field_Strength      = 9.37221[T] (400[MHz])
X_Acq_Duration      = 1.04333312[s]
X_Domain            = 13C
X_Freq              = 100.33735165[MHz]
X_Offset            = 100.0[ppm]
X_Points            = 32768
X_Prescans          = 4
X_Resolution        = 0.95846665[Hz]
X_Sweep             = 31.40703518[kHz]
X_Sweep_Clipped     = 25.12562814[kHz]
Irr_Domain          = Proton
Irr_Freq            = 399.03472754[MHz]
Irr_Offset          = 5.0[ppm]
Clipped             = FALSE
Scans               = 100
Total_Scans         = 100

Relaxation_Delay    = 2[s]
Recvr_Gain          = 50
Temp_Get            = 19.4[dC]
X_90_Width          = 10.9[us]
X_Acq_Time          = 1.04333312[s]
X_Angle             = 30[deg]
X_Atn               = 5.4[dB]
X_Pulse             = 3.63333333[us]
Irr_Atn_Dec         = 25.823[dB]
Irr_Atn_Noe         = 25.823[dB]
Irr_Noise           = WALTZ
Irr_Pwidth          = 0.115[ms]
Decoupling          = TRUE
Initial_Wait        = 1[s]
Noe                 = TRUE
Noe_Time            = 2[s]
Repetition_Time     = 3.04333312[s]
```

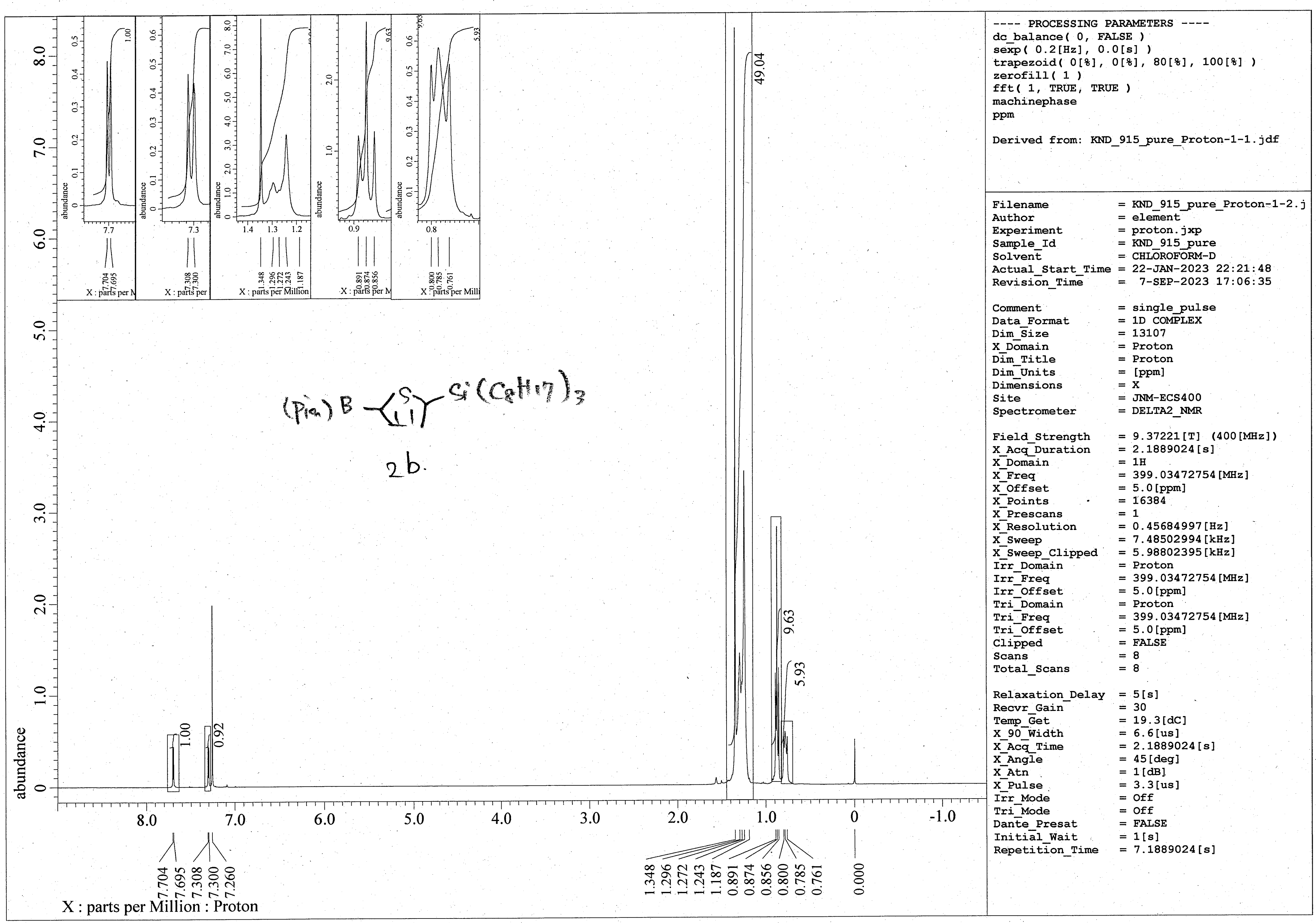
---- PROCESSING PARAMETERS ----
dc_balance(0, FALSE)
sexp(0.2[Hz], 0.0[s])
trapezoid(0[%], 0[%], 80[%], 100[%])
zerofill(1)
fft(1, TRUE, TRUE)
machinephase
ppm
Derived from: KND_915_pure_Proton-1-1.jdf
Filename = KND_915_pure_Proton-1-2.j
Author = element
Experiment = proton.jxp
Sample_Id = KND_915_pure
Solvent = CHLOROFORM-D
Actual_Start_Time = 22-JAN-2023 22:21:48
Revision_Time = 7-SEP-2023 17:06:35
Comment = single_pulse
Data_Format = 1D COMPLEX
Dim_Size = 13107
X_Domain = Proton
Dim_Title = Proton
Dim_Units = [ppm]
Dimensions = X
Site = JNM-ECS400
Spectrometer = DELTA2_NMR
Field_Strength = 9.37221[T] (400[MHz])
X_Acq_Duration = 2.1889024[s]
X_Domain = 1H
X_Freq = 399.03472754[MHz]
X_Offset = 5.0[ppm]
X_Points = 16384
X_Prescans = 1
X_Resolution = 0.45684997[Hz]
X_Sweep = 7.48502994[kHz]
X_Sweep_Clipped = 5.98802395[kHz]
Irr_Domain = Proton
Irr_Freq = 399.03472754[MHz]
Irr_Offset = 5.0[ppm]
Tri_Domain = Proton
Tri_Freq = 399.03472754[MHz]
Tri_Offset = 5.0[ppm]
Clipped = FALSE
Scans = 8
Total_Scans = 8
Relaxation_Delay = 5[s]
Recvr_Gain = 30
Temp_Get = 19.3[dC]
X_90_Width = 6.6[us]
X_Acq_Time = 2.1889024[s]
X_Angle = 45[deg]
X_Atn = 1[dB]
X_Pulse = 3.3[us]
Irr_Mode = Off
Tri_Mode = Off
Dante_Presat = FALSE
Initial_Wait = 1[s]
Repetition_Time = 7.1889024[s]
(Pin) B Si(C8H17)3
2b.
1.00
0.92
49.04
9.63
5.93
7.704 7.695 7.308 7.300 7.260
1.348 1.296 1.272 1.243 1.187 0.891 0.874 0.856 0.800 0.785 0.761
0.000
abundance
X : parts per Million : Proton

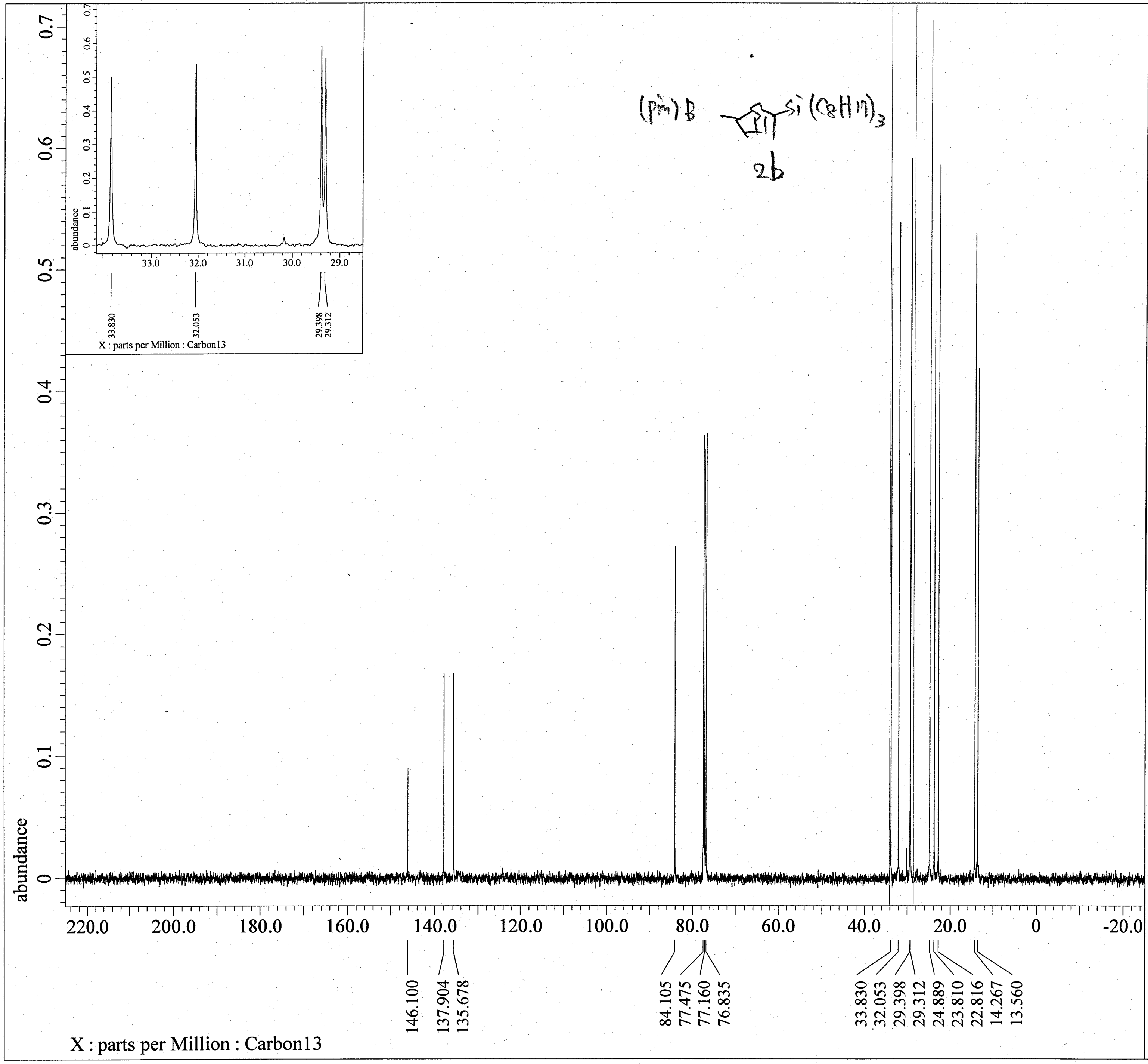

---- PROCESSING PARAMETERS ----
dc_balance(0, FALSE)
sexp(2.0[Hz], 0.0[s])
trapezoid(0[%], 0[%], 80[%], 100[%])
zerofill(1)
fft(1, TRUE, TRUE)
machinephase
ppm

Derived from: KND_915_13C_Carbon-1-1.jdf

Filename	= KND_915_13C_Carbon-1-2.jd
Author	= element
Experiment	= carbon.jxp
Sample_Id	= KND_915_13C
Solvent	= CHLOROFORM-D
Actual_Start_Time	= 22-JAN-2023 22:46:53
Revision_Time	= 22-JAN-2023 22:52:56
Comment	= single pulse decoupled ga
Data_Format	= 1D COMPLEX
Dim_Size	= 26214
X_Domain	= Carbon
Dim_Title	= Carbon13
Dim_Units	= [ppm]
Dimensions	= X
Site	= JNM-ECS400
Spectrometer	= DELTA2_NMR
Field_Strength	= 9.37221[T] (400[MHz])
X_Acq_Duration	= 1.04333312[s]
X_Domain	= 13C
X_Freq	= 100.33735165[MHz]
X_Offset	= 100.0[ppm]
X_Points	= 32768
X_Prescans	= 4
X_Resolution	= 0.95846665[Hz]
X_Sweep	= 31.40703518[kHz]
X_Sweep_Clipped	= 25.12562814[kHz]
Irr_Domain	= Proton
Irr_Freq	= 399.03472754[MHz]
Irr_Offset	= 5.0[ppm]
Clipped	= FALSE
Scans	= 100
Total_Scans	= 100
Relaxation_Delay	= 2[s]
Recvr_Gain	= 50
Temp_Get	= 18.8[dC]
X_90_Width	= 10.9[us]
X_Acq_Time	= 1.04333312[s]
X_Angle	= 30[deg]
X_Atn	= 5.4[dB]
X_Pulse	= 3.63333333[us]
Irr_Atn_Dec	= 25.823[dB]
Irr_Atn_Noe	= 25.823[dB]
Irr_Noise	= WALTZ
Irr_Pwidth	= 0.115[ms]
Decoupling	= TRUE
Initial_Wait	= 1[s]
Noe	= TRUE
Noe_Time	= 2[s]
Repetition_Time	= 3.04333312[s]

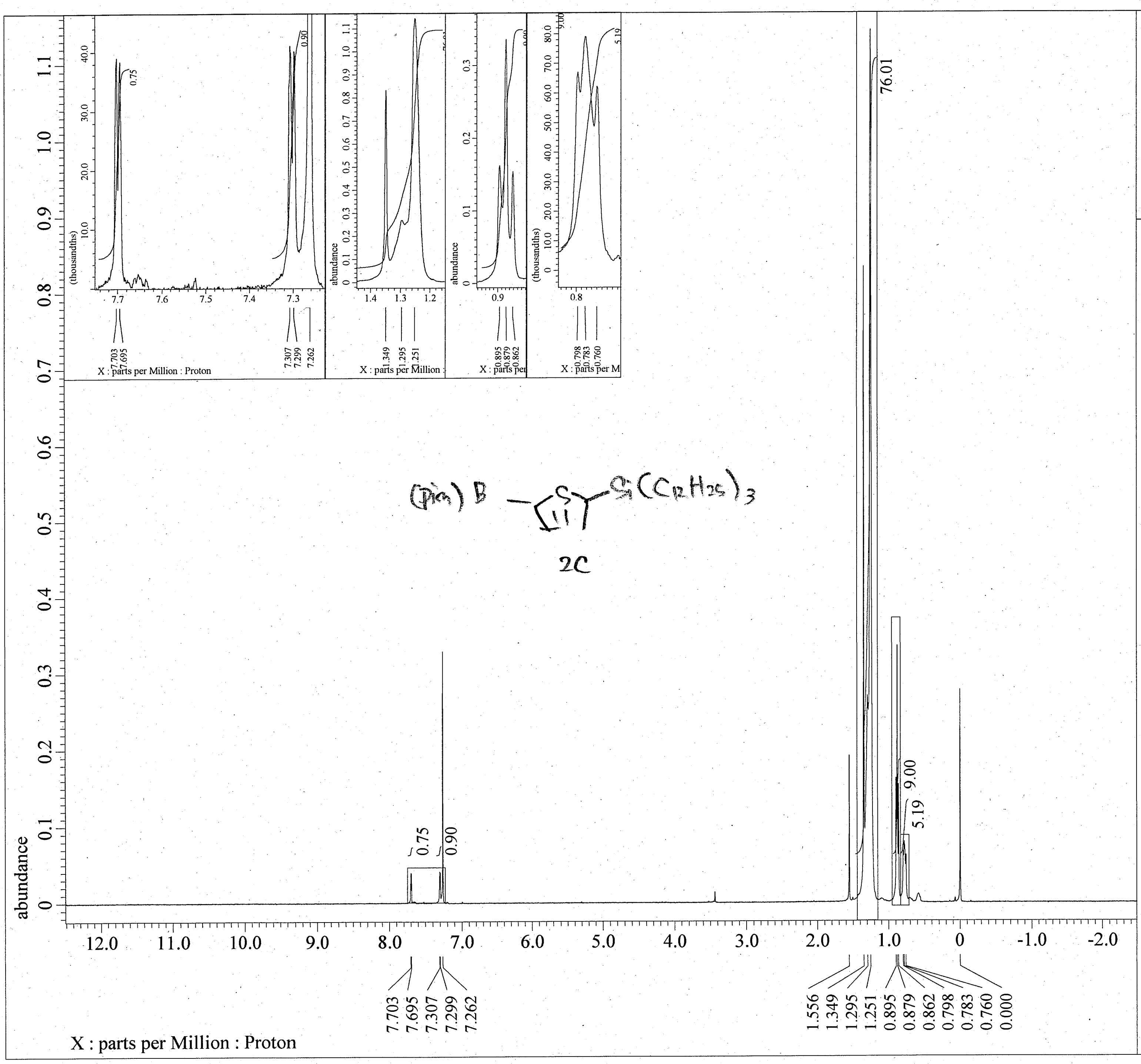

---- PROCESSING PARAMETERS ----
sexp(0.2[Hz], 0.0[s])
trapezoid(0[%], 0[%], 80[%], 100[%])
zerofill(1)
fft(1, TRUE, TRUE)
machinephase
ppm

Derived from: KND_916_pure_Proton-1-1.jdf

Filename	= KND_916_pure_Proton-1
Author	= element
Experiment	= proton_auto.jxp
Sample_Id	= KND_916_pure
Solvent	= CHLOROFORM-D
Actual_Start_Time	= 21-JAN-2023 18:47:27
Revision_Time	= 7-SEP-2023 17:21:19
Comment	= single_pulse
Data_Format	= 1D COMPLEX
Dim_Size	= 13107
X_Domain	= Proton
Dim_Title	= Proton
Dim_Units	= [ppm]
Dimensions	= X
Spectrometer	= DELTA2_NMR
Field_Strength	= 9.2982153[T] (400[MHz
X_Acq_Duration	= 2.20725248[s]
X_Domain	= Proton
X_Freq	= 395.88430144[MHz]
X_Offset	= 5[ppm]
X_Points	= 16384
X_Prescans	= 1
X_Resolution	= 0.45305193[Hz]
X_Sweep	= 7.42280285[kHz]
X_Sweep_Clipped	= 5.93824228[kHz]
Irr_Domain	= Proton
Irr_Freq	= 395.88430144[MHz]
Irr_Offset	= 5[ppm]
Tri_Domain	= Proton
Tri_Freq	= 395.88430144[MHz]
Tri_Offset	= 5[ppm]
Blanking	= 2[us]
Clipped	= TRUE
Scans	= 8
Total_Scans	= 8
Relaxation_Delay	= 5[s]
Recvr_Gain	= 56
Temp_Get	= 17.5[dC]
X_90_Width	= 9.5[us]
X_Acq_Time	= 2.20725248[s]
X_Angle	= 45[deg]
X_Atn	= 3.4[dB]
X_Pulse	= 4.75[us]
Irr_Mode	= Off
Tri_Mode	= Off
Dante_Loop	= 500
Dante_Presat	= FALSE
Decimation_Rate	= 0
Experiment_Path	= C:\Program Files\JEOL
Initial_Wait	= 1[s]
Phase	= {0, 90, 270, 180, 180
Presat_Time	= 5[s]

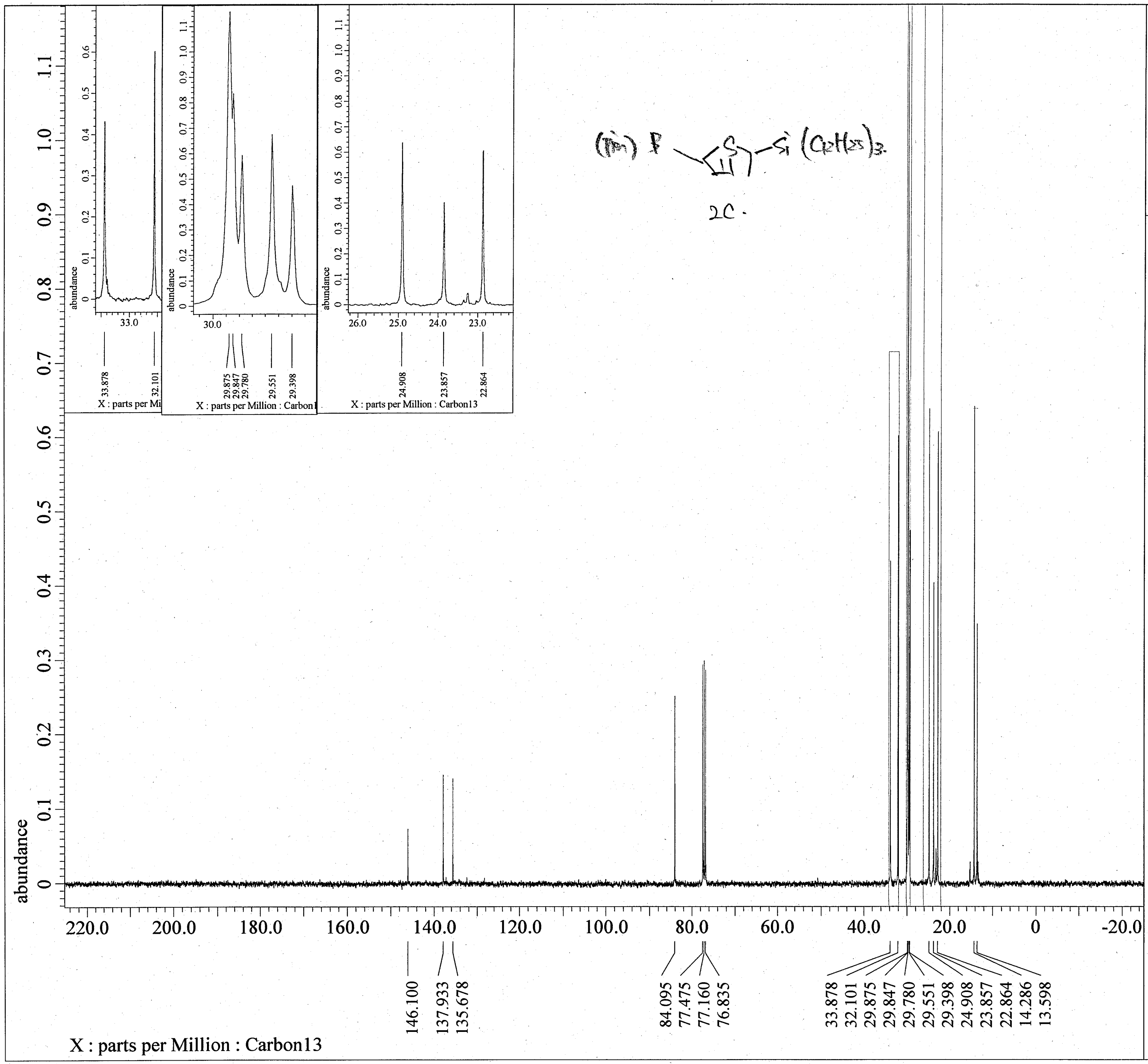


```
---- PROCESSING PARAMETERS ----
dc_balance( 0, FALSE )
sexp( 2.0[Hz], 0.0[s] )
trapezoid( 0[%], 0[%], 80[%], 100[%] )
zerofill( 1 )
fft( 1, TRUE, TRUE )
machinephase
ppm

Derived from: KND_916_13C_Carbon-1-1.jdf
```

```
Filename            = KND_916_13C_Carbon-1-2.jd
Author              = element
Experiment          = carbon.jxp
Sample_Id           = KND_916_13C
Solvent             = CHLOROFORM-D
Actual_Start_Time   = 21-JAN-2023 20:02:39
Revision_Time       = 21-JAN-2023 19:37:44

Comment             = single pulse decoupled ga
Data_Format         = 1D COMPLEX
Dim_Size            = 26214
X_Domain            = Carbon
Dim_Title           = Carbon13
Dim_Units           = [ppm]
Dimensions          = X
Site                = JNM-ECS400
Spectrometer        = DELTA2_NMR

Field_Strength      = 9.37221[T] (400[MHz])
X_Acq_Duration      = 1.04333312[s]
X_Domain            = 13C
X_Freq              = 100.33735165[MHz]
X_Offset            = 100.0[ppm]
X_Points            = 32768
X_Prescans          = 4
X_Resolution        = 0.95846665[Hz]
X_Sweep             = 31.40703518[kHz]
X_Sweep_Clipped     = 25.12562814[kHz]
Irr_Domain          = Proton
Irr_Freq            = 399.03472754[MHz]
Irr_Offset          = 5.0[ppm]
Clipped             = FALSE
Scans               = 150
Total_Scans         = 150

Relaxation_Delay    = 2[s]
Recvr_Gain          = 50
Temp_Get            = 19.4[dC]
X_90_Width          = 10.9[us]
X_Acq_Time          = 1.04333312[s]
X_Angle             = 30[deg]
X_Atn               = 5.4[dB]
X_Pulse             = 3.63333333[us]
Irr_Atn_Dec         = 25.823[dB]
Irr_Atn_Noe         = 25.823[dB]
Irr_Noise           = WALTZ
Irr_Pwidth          = 0.115[ms]
Decoupling          = TRUE
Initial_Wait        = 1[s]
Noe                 = TRUE
Noe_Time            = 2[s]
Repetition_Time     = 3.04333312[s]
```

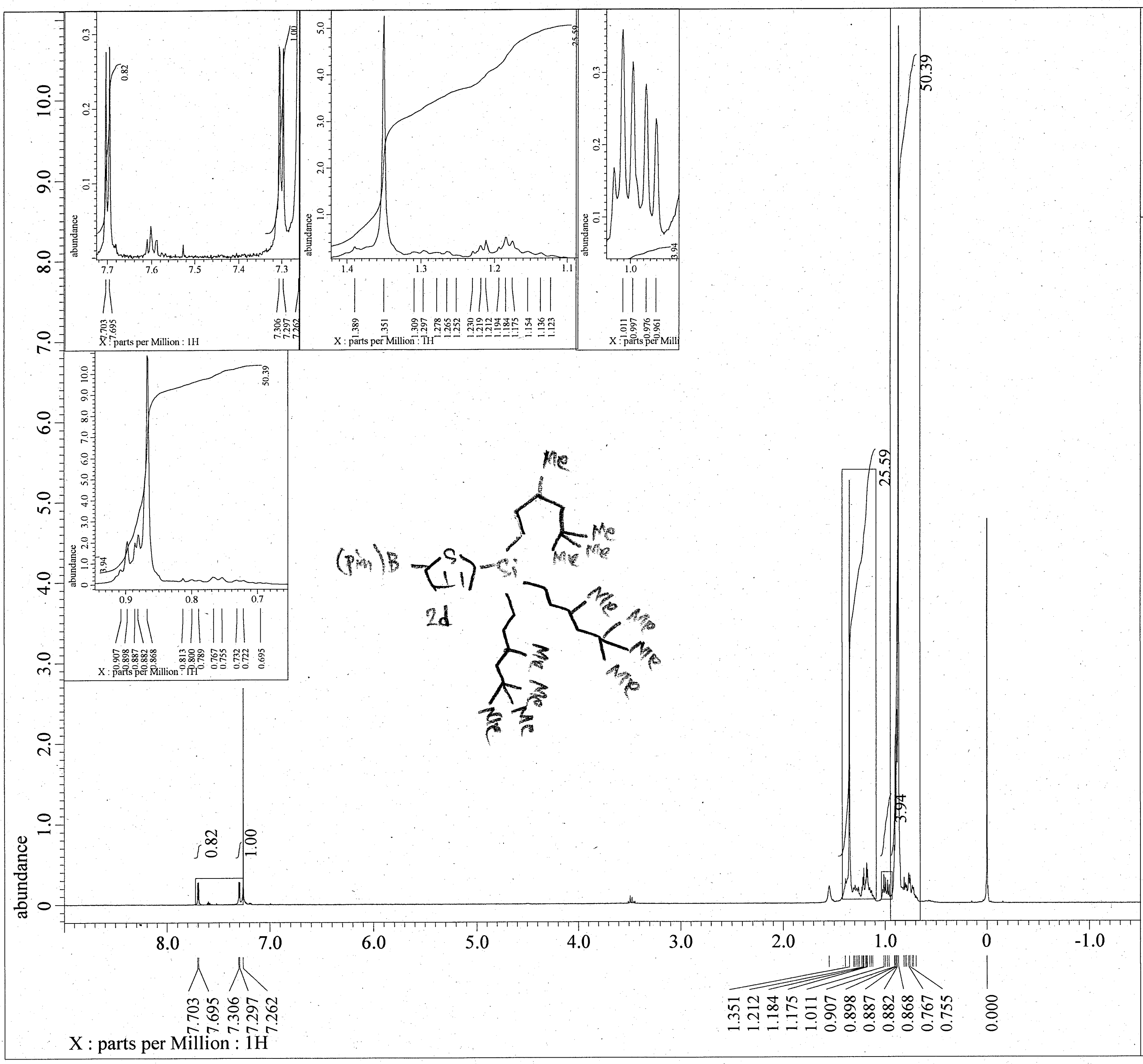


```
---- PROCESSING PARAMETERS ----
dc_balance( 0, FALSE )
sexp( 0.2[Hz], 0.0[s] )
trapezoid3( 0[%], 80[%], 100[%] )
zerofill( 1 )
fft( 1, TRUE, TRUE )
machinephase
ppm

Derived from: KND_675_pure.jdf
```

```
Filename            = KND_675_pure-1.jdf
Author              = element
Experiment          = single_pulse.ex2
Sample_Id           = S#458512
Solvent             = CHLOROFORM-D
Actual_Start_Time   = 29-NOV-2022 21:42:21
Revision_Time       =  7-SEP-2023 17:43:52

Comment             = single_pulse
Data_Format         = 1D COMPLEX
Dim_Size            = 13107
X_Domain            = 1H
Dim_Title           = 1H
Dim_Units           = [ppm]
Dimensions          = X
Site                = ECS 400
Spectrometer        = JNM-ECS400

Field_Strength      = 9.20197068[T] (390[MHz])
X_Acq_Duration      = 2.228224[s]
X_Domain            = 1H
X_Freq              = 391.78655441[MHz]
X_Offset            = 5[ppm]
X_Points            = 16384
X_Prescans          = 1
X_Resolution        = 0.44878791[Hz]
X_Sweep             = 7.35294118[kHz]
Irr_Domain          = 1H
Irr_Freq            = 391.78655441[MHz]
Irr_Offset          = 5[ppm]
Tri_Domain          = 1H
Tri_Freq            = 391.78655441[MHz]
Tri_Offset          = 5[ppm]
Clipped             = FALSE
Scans               = 8
Total_Scans         = 8

Relaxation_Delay    = 5[s]
Recvr_Gain          = 48
Temp_Get            = 18[dC]
X_90_Width          = 11.2[us]
X_Acq_Time          = 2.228224[s]
X_Angle             = 45[deg]
X_Atn               = 1.9[dB]
X_Pulse             = 5.6[us]
Irr_Mode            = Off
Tri_Mode            = Off
Dante_Presat        = FALSE
Initial_Wait        = 1[s]
Repetition_Time     = 7.228224[s]
```

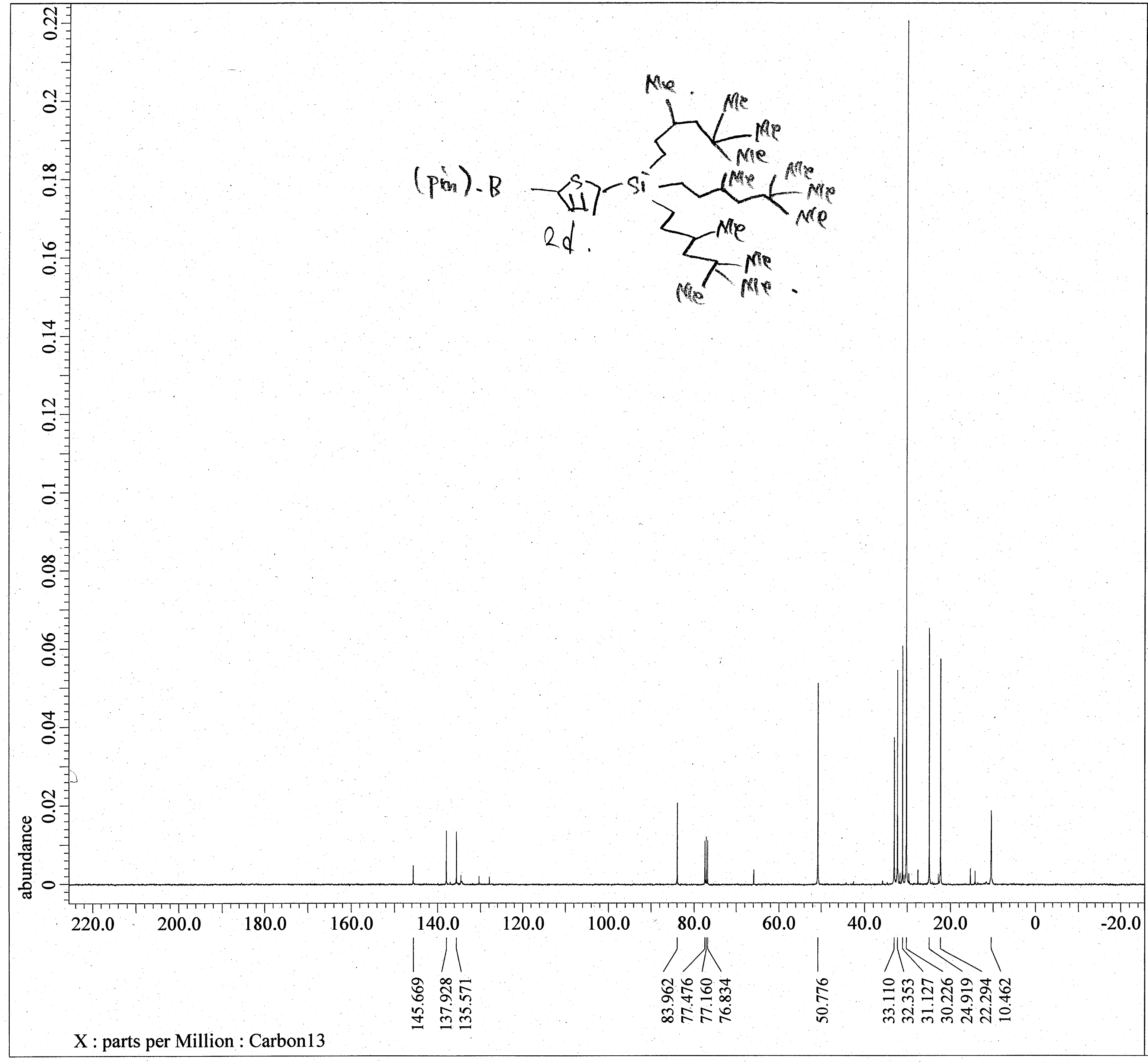


```
---- PROCESSING PARAMETERS ----
sexp( 2.0[Hz], 0.0[s] )
trapezoid( 0[%], 0[%], 80[%], 100[%] )
zerofill( 1 )
fft( 1, TRUE, TRUE )
machinephase
ppm

Derived from: KND_675_13C_Carbon-1-1.jdf
```

```
Filename                  = KND_675_13C_Carbon
Author                    = element
Experiment                = carbon_auto.jxp
Sample_Id                 = KND_675_13C
Solvent                   = CHLOROFORM-D
Actual_Start_Time         = 29-NOV-2022 13:09:
Revision_Time             = 15-JAN-2023 13:36:

Comment                   = single pulse decou
Data_Format               = 1D COMPLEX
Dim_Size                  = 26214
X_Domain                  = Carbon13
Dim_Title                 = Carbon13
Dim_Units                 = [ppm]
Dimensions                = X
Spectrometer              = DELTA2_NMR

Field_Strength            = 9.2982153[T] (400[
X_Acq_Duration            = 1.048576[s]
X_Domain                  = Carbon13
X_Freq                    = 99.54517646[MHz]
X_Offset                  = 100[ppm]
X_Points                  = 32768
X_Prescans                = 4
X_Resolution              = 0.95367432[Hz]
X_Sweep                   = 31.25[kHz]
X_Sweep_Clipped           = 25[kHz]
Irr_Domain                = Proton
Irr_Freq                  = 395.88430144[MHz]
Irr_Offset                = 5[ppm]
Blanking                  = 5[us]
Clipped                   = TRUE
Scans                     = 100
Total_Scans               = 100

Relaxation_Delay          = 2[s]
Recvr_Gain                = 50
Temp_Get                  = 17.1[dC]
X_90_Width                = 9.65[us]
X_Acq_Time                = 1.048576[s]
X_Angle                   = 30[deg]
X_Atn                     = 8[dB]
X_Pulse                   = 3.21666667[us]
Irr_Atn_Dec               = 25.059[dB]
Irr_Atn_Dec_Calc          = 25.059[dB]
Irr_Atn_Dec_Default_Calc  = 25.059[dB]
Irr_Atn_Noe               = 25.059[dB]
Irr_Dec_Bandwidth_Hz      = 4.7826087[kHz]
Irr_Dec_Bandwidth_Ppm     = 12.08082432[ppm]
Irr_Dec_Freq              = 395.88430144[MHz]
Irr_Dec_Merit_Factor      = 2.2
Irr_Decoupling            = TRUE
Irr_Noe                   = TRUE
Irr_Noise                 = WALTZ
Irr_Offset_Default        = 5[ppm]
```

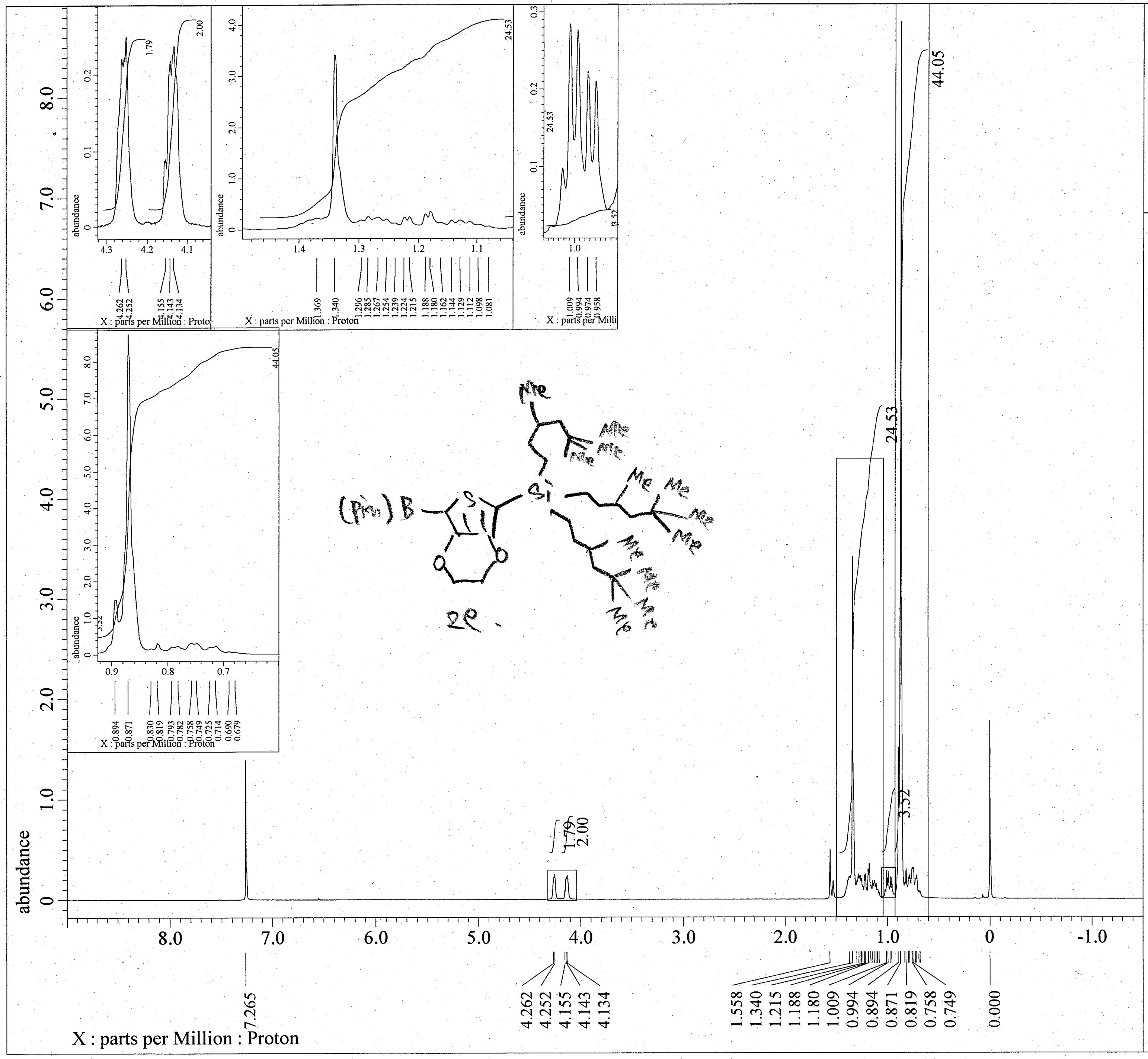


```
---- PROCESSING PARAMETERS ----
dc_balance( 0, FALSE )
sexp( 0.2[Hz], 0.0[s] )
trapezoid( 0[%], 0[%], 80[%], 100[%] )
zerofill( 1 )
fft( 1, TRUE, TRUE )
machinephase
ppm

Derived from: KND_554_pure_Proton-1-1.jdf
```

```
Filename           = KND_554_pure_Proton-1-2.j
Author             = element
Experiment         = proton.jxp
Sample_Id          = KND_554_pure
Solvent            = CHLOROFORM-D
Actual_Start_Time  = 30-AUG-2022 21:39:16
Revision_Time      =  7-SEP-2023 17:53:43

Comment            = single_pulse
Data_Format        = 1D COMPLEX
Dim_Size           = 13107
X_Domain           = Proton
Dim_Title          = Proton
Dim_Units          = [ppm]
Dimensions         = X
Site               = JNM-ECS400
Spectrometer       = DELTA2_NMR

Field_Strength     = 9.37221[T] (400[MHz])
X_Acq_Duration     = 2.1889024[s]
X_Domain           = 1H
X_Freq             = 399.03472754[MHz]
X_Offset           = 5.0[ppm]
X_Points           = 16384
X_Prescans         = 1
X_Resolution       = 0.45684997[Hz]
X_Sweep            = 7.48502994[kHz]
X_Sweep_Clipped    = 5.98802395[kHz]
Irr_Domain         = Proton
Irr_Freq           = 399.03472754[MHz]
Irr_Offset         = 5.0[ppm]
Tri_Domain         = Proton
Tri_Freq           = 399.03472754[MHz]
Tri_Offset         = 5.0[ppm]
Clipped            = FALSE
Scans              = 8
Total_Scans        = 8

Relaxation_Delay   = 5[s]
Recvr_Gain         = 40
Temp_Get           = 20.6[dC]
X_90_Width         = 6.6[us]
X_Acq_Time         = 2.1889024[s]
X_Angle            = 45[deg]
X_Atn              = 1[dB]
X_Pulse            = 3.3[us]
Irr_Mode           = Off
Tri_Mode           = Off
Dante_Presat       = FALSE
Initial_Wait       = 1[s]
Repetition_Time    = 7.1889024[s]
```

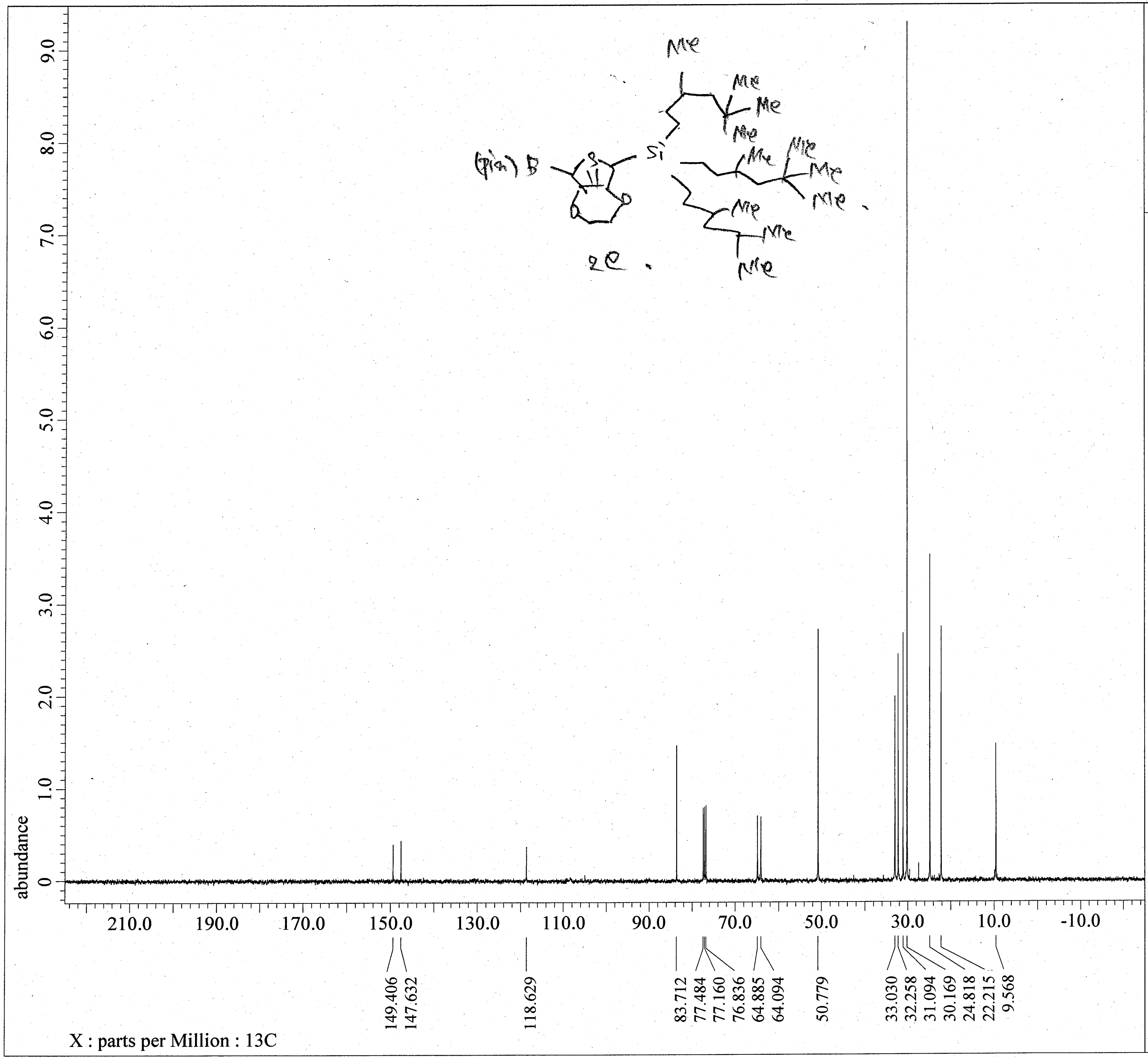


```
---- PROCESSING PARAMETERS ----
dc_balance( 0, FALSE )
sexp( 2.0[Hz], 0.0[s] )
trapezoid3( 0[%], 80[%], 100[%] )
zerofill( 1 )
fft( 1, TRUE, TRUE )
machinephase
ppm

Derived from: KND_554_13C-1.jdf

Filename            = KND_554_13C-5.jdf
Author              = element
Experiment          = single_pulse_dec
Sample_Id           = 1
Solvent             = CHLOROFORM-D
Actual_Start_Time   = 31-AUG-2022 03:38:02
Revision_Time       = 17-OCT-2022 13:21:27

Comment             = single pulse decoupled ga
Data_Format         = 1D COMPLEX
Dim_Size            = 26214
X_Domain            = 13C
Dim_Title           = 13C
Dim_Units           = [ppm]
Dimensions          = X
Site                = ECS 400
Spectrometer        = JNM-ECS400

Field_Strength      = 9.20197068[T] (390[MHz])
X_Acq_Duration      = 1.06430464[s]
X_Domain            = 13C
X_Freq              = 98.51479726[MHz]
X_Offset            = 100[ppm]
X_Points            = 32768
X_Prescans          = 4
X_Resolution        = 0.93958061[Hz]
X_Sweep             = 30.78817734[kHz]
Irr_Domain          = 1H
Irr_Freq            = 391.78655441[MHz]
Irr_Offset          = 5[ppm]
Clipped             = FALSE
Scans               = 34
Total_Scans         = 34

Relaxation_Delay    = 2[s]
Recvr_Gain          = 60
Temp_Get            = 21.3[dC]
X_90_Width          = 11[us]
X_Acq_Time          = 1.06430464[s]
X_Angle             = 30[deg]
X_Atn               = 4.9[dB]
X_Pulse             = 3.66666667[us]
Irr_Atn_Dec         = 22.05[dB]
Irr_Atn_Noe         = 22.05[dB]
Irr_Noise           = WALTZ
Decoupling          = TRUE
Initial_Wait        = 1[s]
Noe                 = TRUE
Noe_Time            = 2[s]
Repetition_Time     = 3.06430464[s]
```

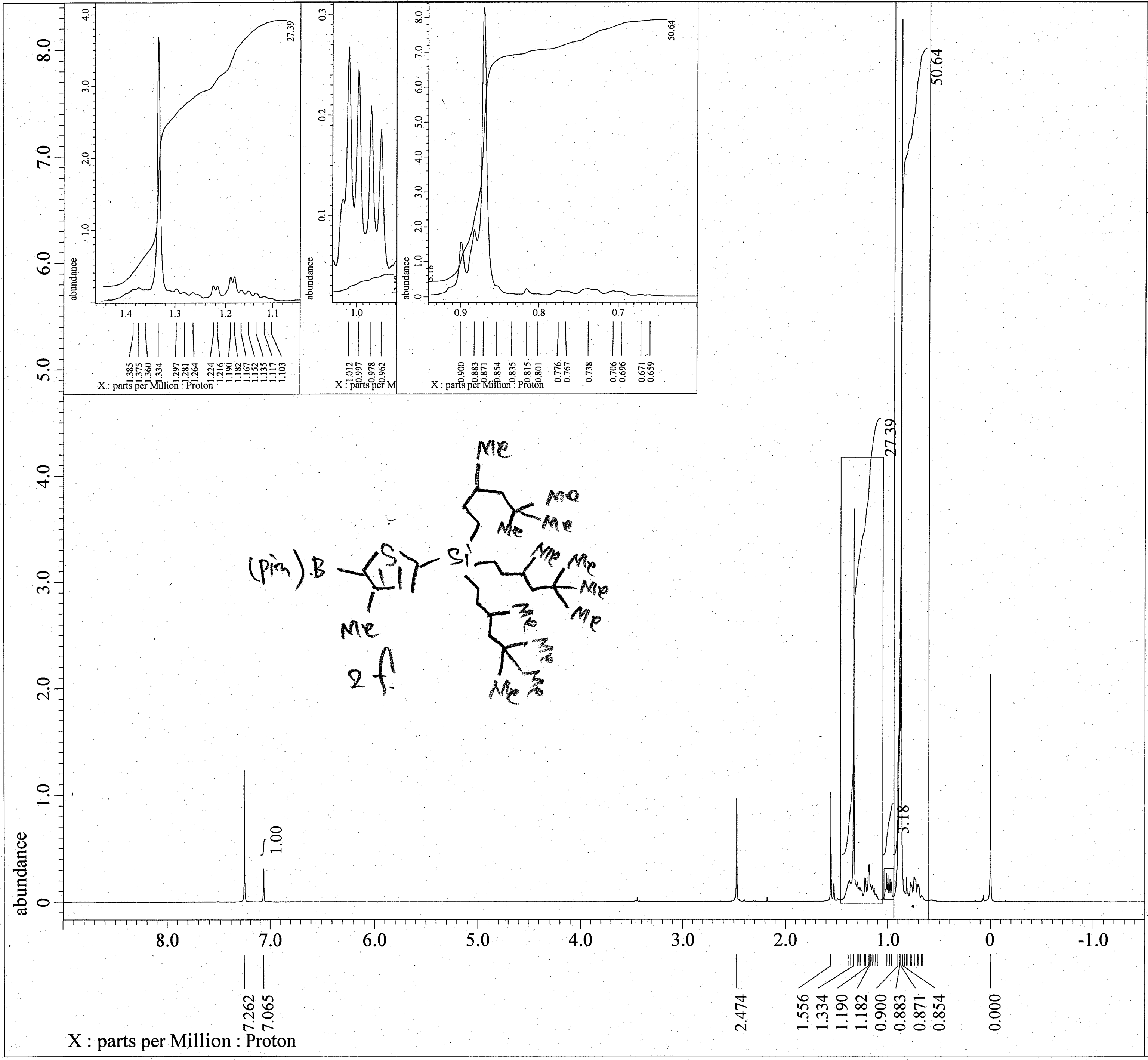


```
---- PROCESSING PARAMETERS ----
dc_balance( 0, FALSE )
sexp( 0.2[Hz], 0.0[s] )
trapezoid( 0[%], 0[%], 80[%], 100[%] )
zerofill( 1 )
fft( 1, TRUE, TRUE )
machinephase
ppm

Derived from: KND_553_pure3_Proton-1-1.jdf

Filename            = KND_553_pure3_Proton-1-2.
Author              = element
Experiment          = proton.jxp
Sample_Id           = KND_553_pure
Solvent             = CHLOROFORM-D
Actual_Start_Time   =  2-OCT-2022 16:08:39
Revision_Time       =  7-SEP-2023 18:02:31

Comment             = single_pulse
Data_Format         = 1D COMPLEX
Dim_Size            = 13107
X_Domain            = Proton
Dim_Title           = Proton
Dim_Units           = [ppm]
Dimensions          = X
Site                = JNM-ECS400
Spectrometer        = DELTA2_NMR

Field_Strength      = 9.37221[T] (400[MHz])
X_Acq_Duration      = 2.1889024[s]
X_Domain            = 1H
X_Freq              = 399.03472754[MHz]
X_Offset            = 5.0[ppm]
X_Points            = 16384
X_Prescans          = 1
X_Resolution        = 0.45684997[Hz]
X_Sweep             = 7.48502994[kHz]
X_Sweep_Clipped     = 5.98802395[kHz]
Irr_Domain          = Proton
Irr_Freq            = 399.03472754[MHz]
Irr_Offset          = 5.0[ppm]
Tri_Domain          = Proton
Tri_Freq            = 399.03472754[MHz]
Tri_Offset          = 5.0[ppm]
Clipped             = FALSE
Scans               = 8
Total_Scans         = 8

Relaxation_Delay    = 5[s]
Recvr_Gain          = 40
Temp_Get            = 19.6[dC]
X_90_Width          = 6.6[us]
X_Acq_Time          = 2.1889024[s]
X_Angle             = 45[deg]
X_Atn               = 1[dB]
X_Pulse             = 3.3[us]
Irr_Mode            = Off
Tri_Mode            = Off
Dante_Presat        = FALSE
Initial_Wait        = 1[s]
Repetition_Time     = 7.1889024[s]
```

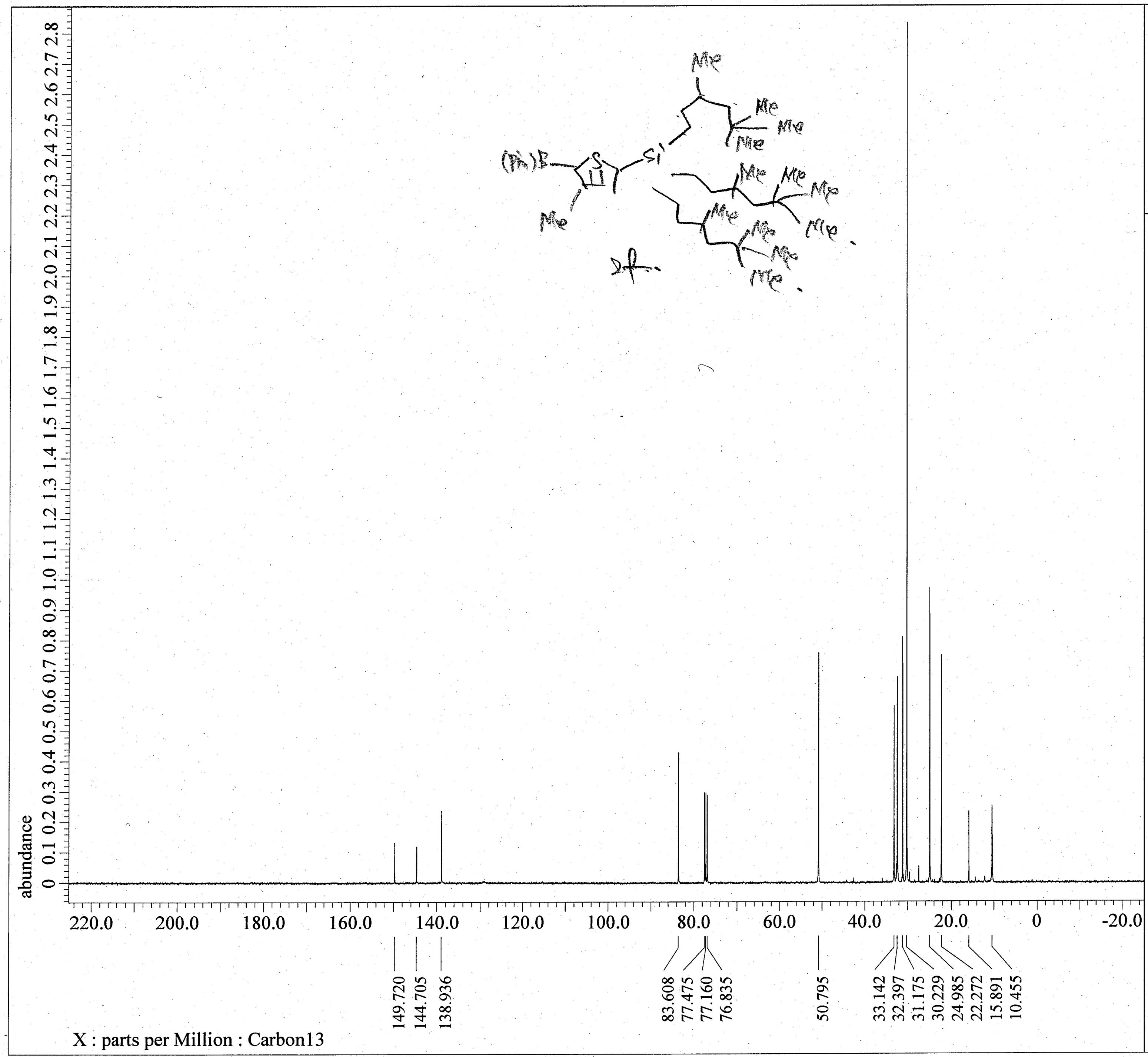

---- PROCESSING PARAMETERS ----
dc_balance(0, FALSE)
sexp(2.0[Hz], 0.0[s])
trapezoid(0[%], 0[%], 80[%], 100[%])
zerofill(1)
fft(1, TRUE, TRUE)
machinephase
ppm

Derived from: KND_553_13C_Carbon-1-1.jdf

Filename	= KND_553_13C_Carbon-1-2.jd
Author	= element
Experiment	= carbon.jxp
Sample_Id	= KND_553_13C
Solvent	= CHLOROFORM-D
Actual_Start_Time	= 2-OCT-2022 16:31:45
Revision_Time	= 8-OCT-2022 16:49:38
Comment	= single pulse decoupled ga
Data_Format	= 1D COMPLEX
Dim_Size	= 26214
X_Domain	= Carbon
Dim_Title	= Carbon13
Dim_Units	= [ppm]
Dimensions	= X
Site	= JNM-ECS400
Spectrometer	= DELTA2_NMR
Field_Strength	= 9.37221[T] (400[MHz])
X_Acq_Duration	= 1.04333312[s]
X_Domain	= 13C
X_Freq	= 100.33735165[MHz]
X_Offset	= 100.0[ppm]
X_Points	= 32768
X_Prescans	= 4
X_Resolution	= 0.95846665[Hz]
X_Sweep	= 31.40703518[kHz]
X_Sweep_Clipped	= 25.12562814[kHz]
Irr_Domain	= Proton
Irr_Freq	= 399.03472754[MHz]
Irr_Offset	= 5.0[ppm]
Clipped	= FALSE
Scans	= 200
Total_Scans	= 200
Relaxation_Delay	= 2[s]
Recvr_Gain	= 50
Temp_Get	= 19.6[dC]
X_90_Width	= 10.9[us]
X_Acq_Time	= 1.04333312[s]
X_Angle	= 30[deg]
X_Atn	= 5.4[dB]
X_Pulse	= 3.63333333[us]
Irr_Atn_Dec	= 25.823[dB]
Irr_Atn_Noe	= 25.823[dB]
Irr_Noise	= WALTZ
Irr_Pwidth	= 0.115[ms]
Decoupling	= TRUE
Initial_Wait	= 1[s]
Noe	= TRUE
Noe_Time	= 2[s]
Repetition_Time	= 3.04333312[s]

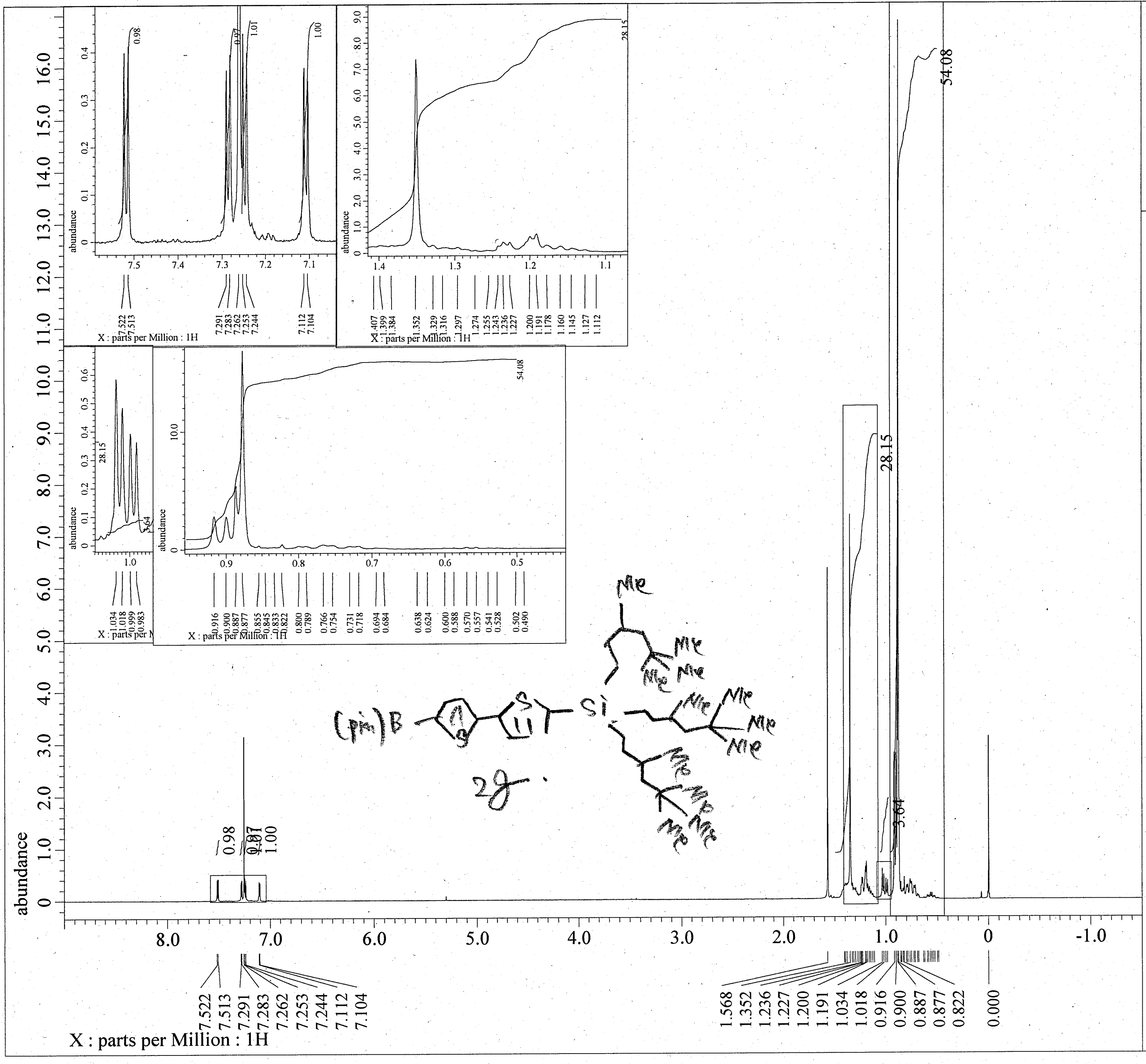


```
---- PROCESSING PARAMETERS ----
dc_balance( 0, FALSE )
sexp( 0.2[Hz], 0.0[s] )
trapezoid3( 0[%], 80[%], 100[%] )
zerofill( 1 )
fft( 1, TRUE, TRUE )
machinephase
ppm

Derived from: KND_556_pure-1.jdf
```

```
Filename            = KND_556_pure-2.jdf
Author              = element
Experiment          = single_pulse.ex2
Sample_Id           = S#795456
Solvent             = CHLOROFORM-D
Actual_Start_Time   =  9-SEP-2022 04:50:40
Revision_Time       =  7-SEP-2023 18:20:18

Comment             = single_pulse
Data_Format         = 1D COMPLEX
Dim_Size            = 13107
X_Domain            = 1H
Dim_Title           = 1H
Dim_Units           = [ppm]
Dimensions          = X
Site                = ECS 400
Spectrometer        = JNM-ECS400

Field_Strength      = 9.20197068[T] (390[MHz])
X_Acq_Duration      = 2.228224[s]
X_Domain            = 1H
X_Freq              = 391.78655441[MHz]
X_Offset            = 5[ppm]
X_Points            = 16384
X_Prescans          = 1
X_Resolution        = 0.44878791[Hz]
X_Sweep             = 7.35294118[kHz]
Irr_Domain          = 1H
Irr_Freq            = 391.78655441[MHz]
Irr_Offset          = 5[ppm]
Tri_Domain          = 1H
Tri_Freq            = 391.78655441[MHz]
Tri_Offset          = 5[ppm]
Clipped             = FALSE
Scans               = 8
Total_Scans         = 8

Relaxation_Delay    = 5[s]
Recvr_Gain          = 46
Temp_Get            = 20.6[dC]
X_90_Width          = 10.8[us]
X_Acq_Time          = 2.228224[s]
X_Angle             = 45[deg]
X_Atn               = 1.9[dB]
X_Pulse             = 5.4[us]
Irr_Mode            = Off
Tri_Mode            = Off
Dante_Presat        = FALSE
Initial_Wait        = 1[s]
Repetition_Time     = 7.228224[s]
```

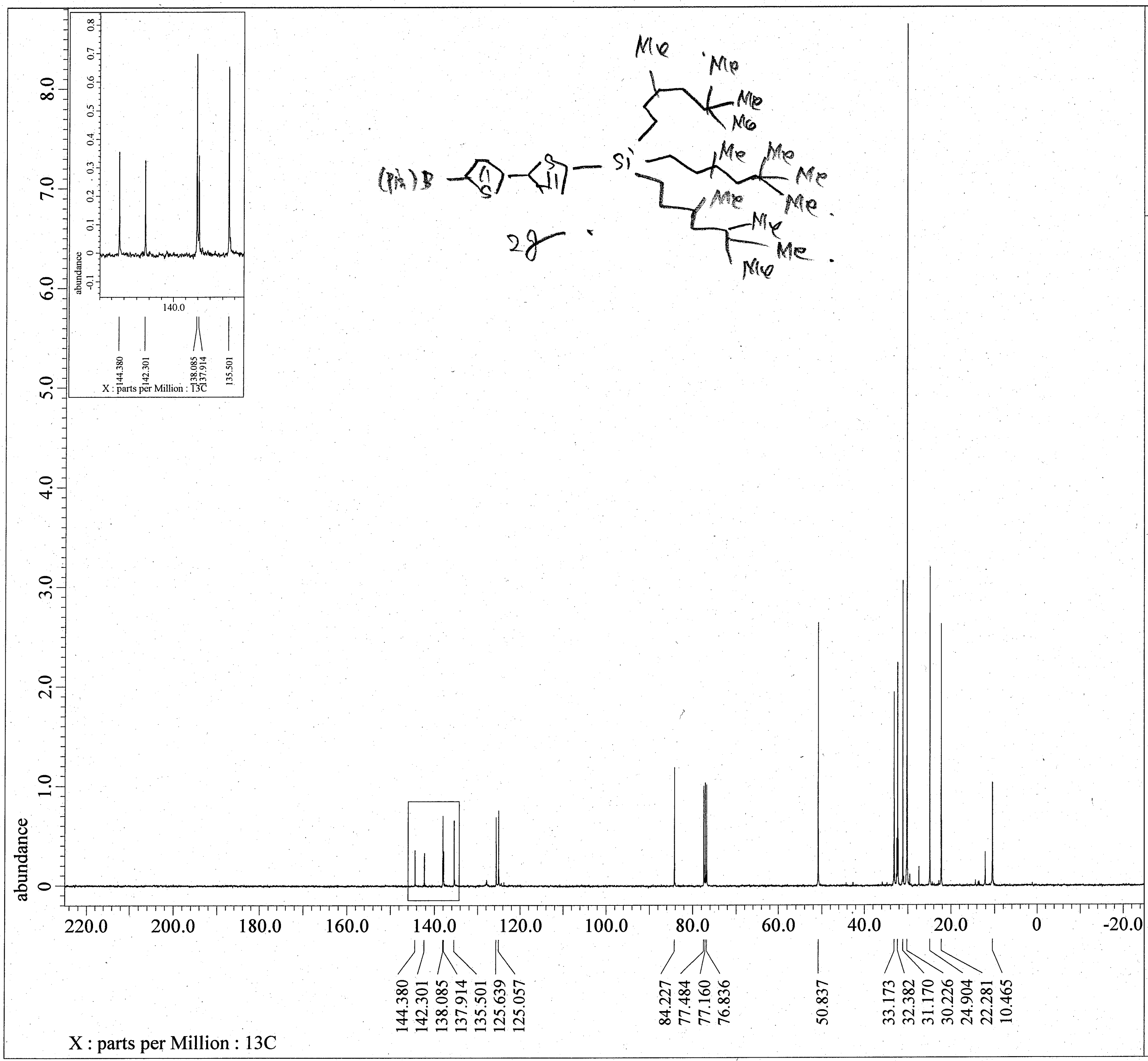


```
---- PROCESSING PARAMETERS ----
dc_balance( 0, FALSE )
sexp( 2.0[Hz], 0.0[s] )
trapezoid3( 0[%], 80[%], 100[%] )
zerofill( 1 )
fft( 1, TRUE, TRUE )
machinephase
ppm

Derived from: KND_556_13C-1.jdf
```

```
Filename            = KND_556_13C-2.jdf
Author              = element
Experiment          = single_pulse_dec
Sample_Id           = 1
Solvent             = CHLOROFORM-D
Actual_Start_Time   =  9-SEP-2022 05:03:03
Revision_Time       = 17-OCT-2022 14:05:56

Comment             = single pulse decoupled ga
Data_Format         = 1D COMPLEX
Dim_Size            = 26214
X_Domain            = 13C
Dim_Title           = 13C
Dim_Units           = [ppm]
Dimensions          = X
Site                = ECS 400
Spectrometer        = JNM-ECS400

Field_Strength      = 9.20197068[T]  (390[MHz])
X_Acq_Duration      = 1.06430464[s]
X_Domain            = 13C
X_Freq              = 98.51479726[MHz]
X_Offset            = 100[ppm]
X_Points            = 32768
X_Prescans          = 4
X_Resolution        = 0.93958061[Hz]
X_Sweep             = 30.78817734[kHz]
Irr_Domain          = 1H
Irr_Freq            = 391.78655441[MHz]
Irr_Offset          = 5[ppm]
Clipped             = FALSE
Scans               = 200
Total_Scans         = 200

Relaxation_Delay    = 2[s]
Recvr_Gain          = 60
Temp_Get            = 20.8[dC]
X_90_Width          = 8.7[us]
X_Acq_Time          = 1.06430464[s]
X_Angle             = 30[deg]
X_Atn               = 4.9[dB]
X_Pulse             = 2.9[us]
Irr_Atn_Dec         = 22.45[dB]
Irr_Atn_Noe         = 22.45[dB]
Irr_Noise           = WALTZ
Decoupling          = TRUE
Initial_Wait        = 1[s]
Noe                 = TRUE
Noe_Time            = 2[s]
Repetition_Time     = 3.06430464[s]
```

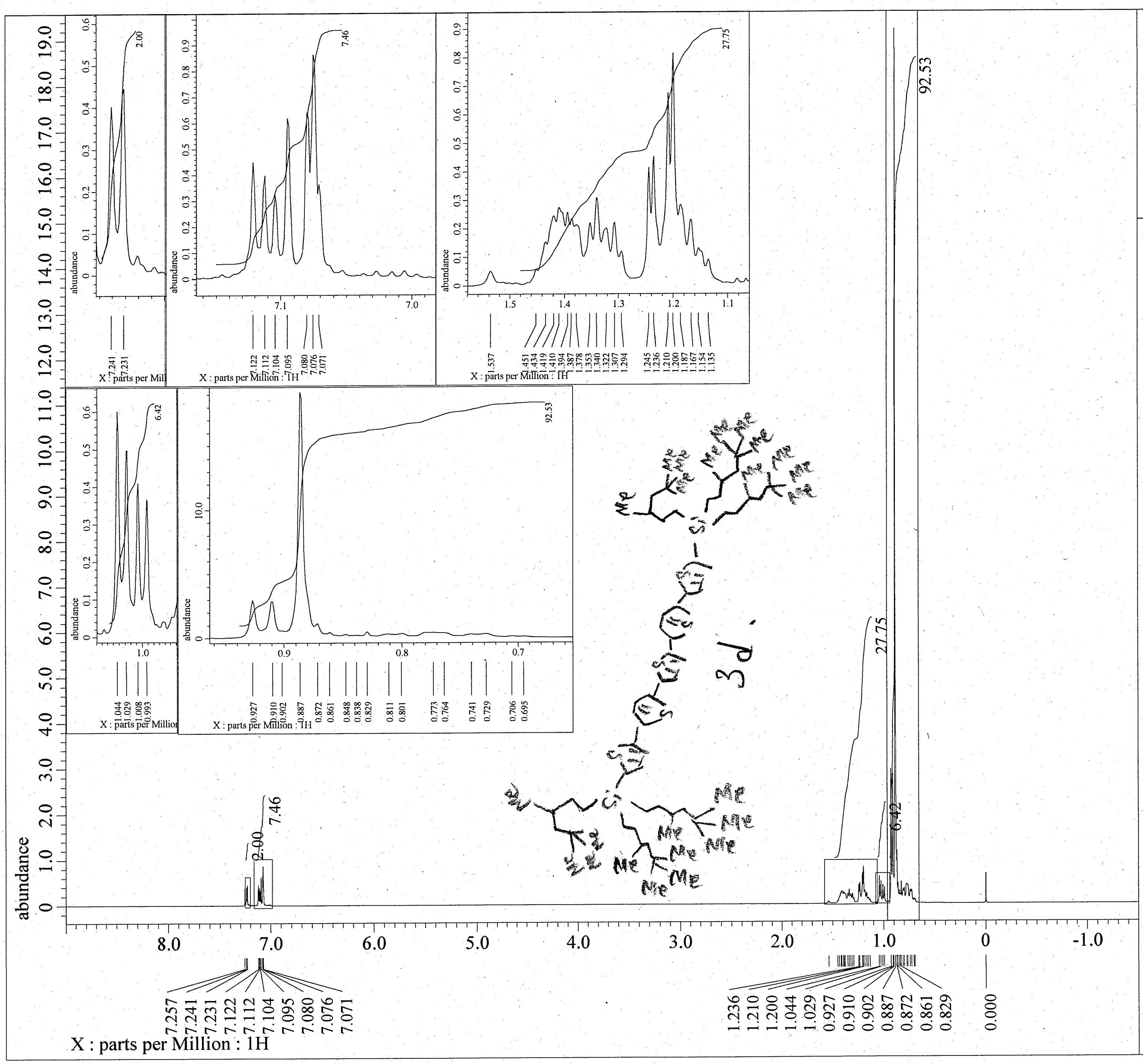


```
---- PROCESSING PARAMETERS ----
dc_balance( 0, FALSE )
sexp( 0.2[Hz], 0.0[s] )
trapezoid3( 0[%], 80[%], 100[%] )
zerofill( 1 )
fft( 1, TRUE, TRUE )
machinephase
ppm

Derived from: KND_682_pure-1.jdf
```

```
Filename            = KND_682_pure-2.jdf
Author              = element
Experiment          = single_pulse.ex2
Sample_Id           = S#587200
Solvent             = CHLOROFORM-D
Actual_Start_Time   = 25-NOV-2022 01:17:31
Revision_Time       =  7-SEP-2023 18:34:52

Comment             = single_pulse
Data_Format         = 1D COMPLEX
Dim_Size            = 13107
X_Domain            = 1H
Dim_Title           = 1H
Dim_Units           = [ppm]
Dimensions          = X
Site                = ECS 400
Spectrometer        = JNM-ECS400

Field_Strength      = 9.20197068[T] (390[MHz])
X_Acq_Duration      = 2.228224[s]
X_Domain            = 1H
X_Freq              = 391.78655441[MHz]
X_Offset            = 5[ppm]
X_Points            = 16384
X_Prescans          = 1
X_Resolution        = 0.44878791[Hz]
X_Sweep             = 7.35294118[kHz]
Irr_Domain          = 1H
Irr_Freq            = 391.78655441[MHz]
Irr_Offset          = 5[ppm]
Tri_Domain          = 1H
Tri_Freq            = 391.78655441[MHz]
Tri_Offset          = 5[ppm]
Clipped             = FALSE
Scans               = 8
Total_Scans         = 8

Relaxation_Delay    = 5[s]
Recvr_Gain          = 32
Temp_Get            = 18.5[dC]
X_90_Width          = 11.2[us]
X_Acq_Time          = 2.228224[s]
X_Angle             = 45[deg]
X_Atn               = 1.9[dB]
X_Pulse             = 5.6[us]
Irr_Mode            = Off
Tri_Mode            = Off
Dante_Presat        = FALSE
Initial_Wait        = 1[s]
Repetition_Time     = 7.228224[s]
```

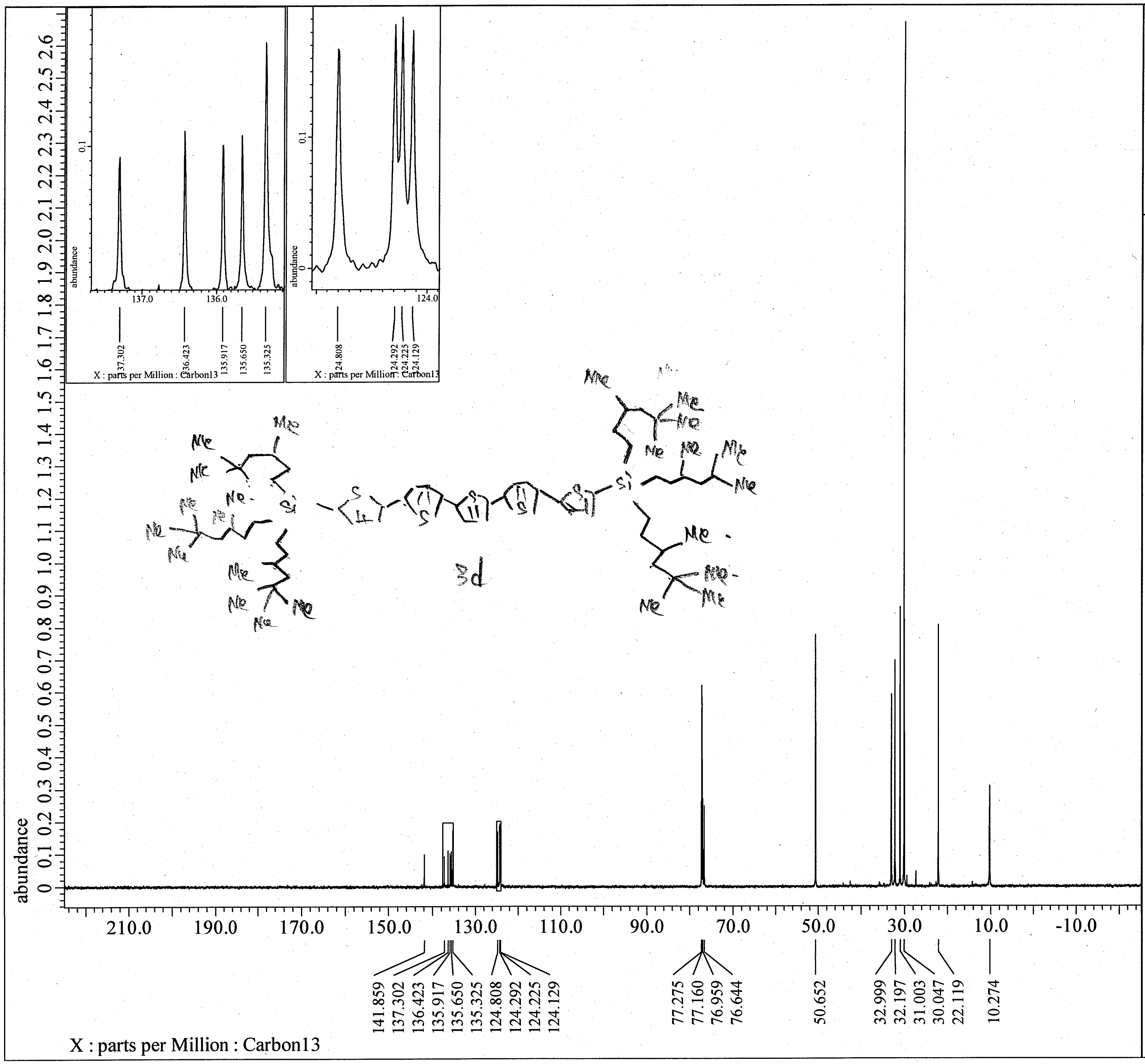

---- PROCESSING PARAMETERS ----
dc_balance(0, FALSE)
sexp(2.0[Hz], 0.0[s])
trapezoid(0[%], 0[%], 80[%], 100[%])
zerofill(1)
fft(1, TRUE, TRUE)
machinephase
ppm

Derived from: KND_682_13C_Carbon-1-1.jdf

Filename	= KND_682_13C_Carbon-1-2.jd
Author	= element
Experiment	= carbon.jxp
Sample_Id	= KND_631_13C
Solvent	= CHLOROFORM-D
Actual_Start_Time	= 24-NOV-2022 20:44:39
Revision_Time	= 25-NOV-2022 09:18:46
Comment	= single pulse decoupled ga
Data_Format	= 1D COMPLEX
Dim_Size	= 26214
X_Domain	= Carbon
Dim_Title	= Carbon13
Dim_Units	= [ppm]
Dimensions	= X
Site	= JNM-ECS400
Spectrometer	= DELTA2_NMR
Field_Strength	= 9.37221[T] (400[MHz])
X_Acq_Duration	= 1.04333312[s]
X_Domain	= 13C
X_Freq	= 100.33735165[MHz]
X_Offset	= 100.0[ppm]
X_Points	= 32768
X_Prescans	= 4
X_Resolution	= 0.95846665[Hz]
X_Sweep	= 31.40703518[kHz]
X_Sweep_Clipped	= 25.12562814[kHz]
Irr_Domain	= Proton
Irr_Freq	= 399.03472754[MHz]
Irr_Offset	= 5.0[ppm]
Clipped	= FALSE
Scans	= 200
Total_Scans	= 200
Relaxation_Delay	= 2[s]
Recvr_Gain	= 50
Temp_Get	= 18.3[dC]
X_90_Width	= 10.9[us]
X_Acq_Time	= 1.04333312[s]
X_Angle	= 30[deg]
X_Atn	= 5.4[dB]
X_Pulse	= 3.63333333[us]
Irr_Atn_Dec	= 25.823[dB]
Irr_Atn_Noe	= 25.823[dB]
Irr_Noise	= WALTZ
Irr_Pwidth	= 0.115[ms]
Decoupling	= TRUE
Initial_Wait	= 1[s]
Noe	= TRUE
Noe_Time	= 2[s]
Repetition_Time	= 3.04333312[s]

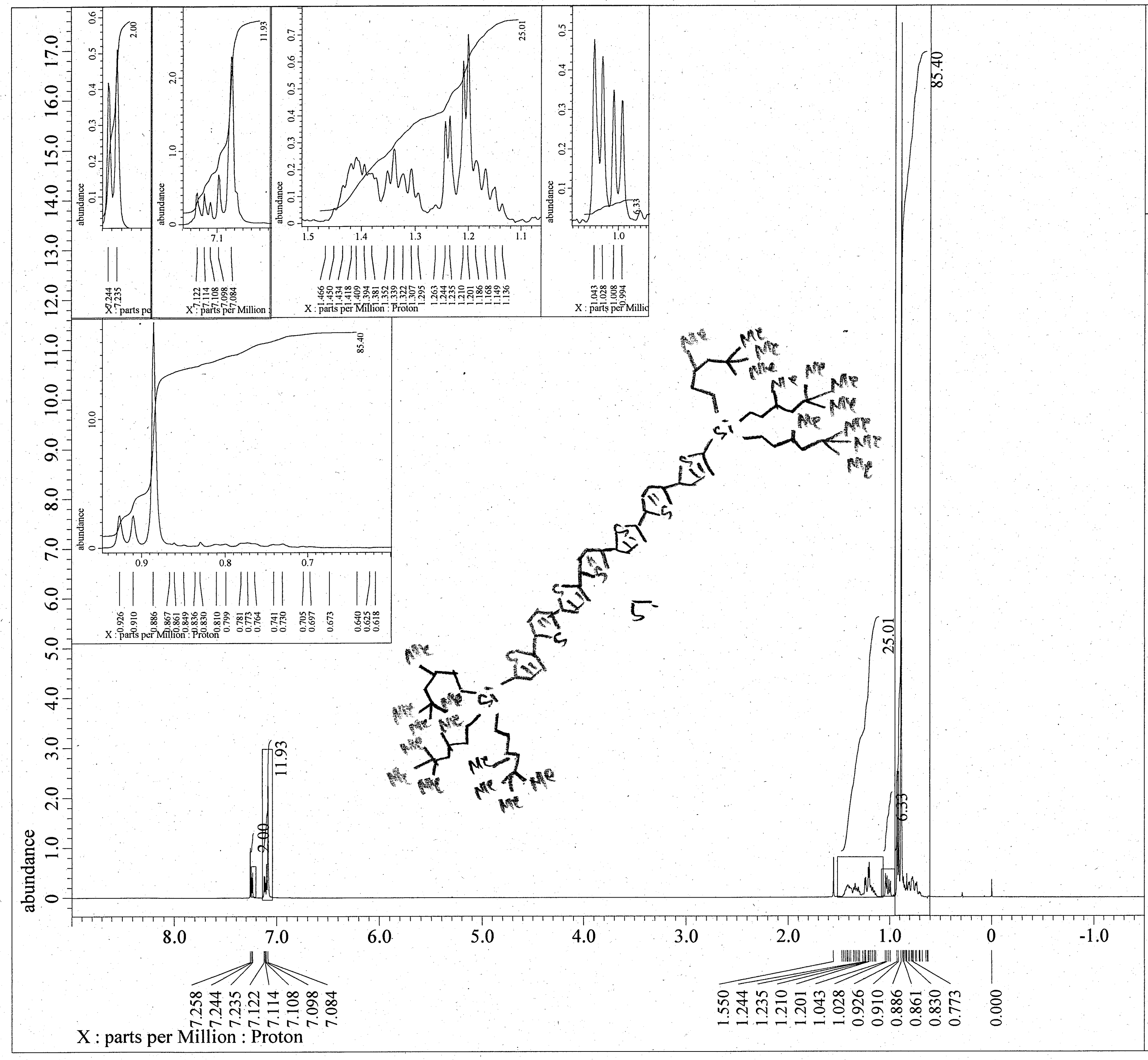


```
---- PROCESSING PARAMETERS ----
dc_balance( 0, FALSE )
sexp( 0.2[Hz], 0.0[s] )
trapezoid( 0[%], 0[%], 80[%], 100[%] )
zerofill( 1 )
fft( 1, TRUE, TRUE )
machinephase
ppm

Derived from: KND_298_pure_Proton-1-1.jdf
```

```
Filename            = KND_298_pure_Proton-1-2.j
Author              = element
Experiment          = proton.jxp
Sample_Id           = KND_298_pure
Solvent             = CHLOROFORM-D
Actual_Start_Time   = 24-MAR-2022 11:39:57
Revision_Time       =  7-SEP-2023 18:42:35

Comment             = single_pulse
Data_Format         = 1D COMPLEX
Dim_Size            = 13107
X_Domain            = Proton
Dim_Title           = Proton
Dim_Units           = [ppm]
Dimensions          = X
Spectrometer        = DELTA2_NMR

Field_Strength      = 9.4073814[T] (400[MHz])
X_Acq_Duration      = 2.18103808[s]
X_Domain            = 1H
X_Freq              = 400.53219825[MHz]
X_Offset            = 5[ppm]
X_Points            = 16384
X_Prescans          = 1
X_Resolution        = 0.45849727[Hz]
X_Sweep             = 7.51201923[kHz]
X_Sweep_Clipped     = 6.00961538[kHz]
Irr_Domain          = Proton
Irr_Freq            = 400.53219825[MHz]
Irr_Offset          = 5[ppm]
Tri_Domain          = Proton
Tri_Freq            = 400.53219825[MHz]
Tri_Offset          = 5[ppm]
Clipped             = FALSE
Scans               = 8
Total_Scans         = 8

Relaxation_Delay    = 5[s]
Recvr_Gain          = 42
Temp_Get            = 18.1[dC]
X_90_Width          = 6[us]
X_Acq_Time          = 2.18103808[s]
X_Angle             = 45[deg]
X_Atn               = 0.8[dB]
X_Pulse             = 3[us]
Irr_Mode            = Off
Tri_Mode            = Off
Dante_Presat        = FALSE
Initial_Wait        = 1[s]
Repetition_Time     = 7.18103808[s]
```

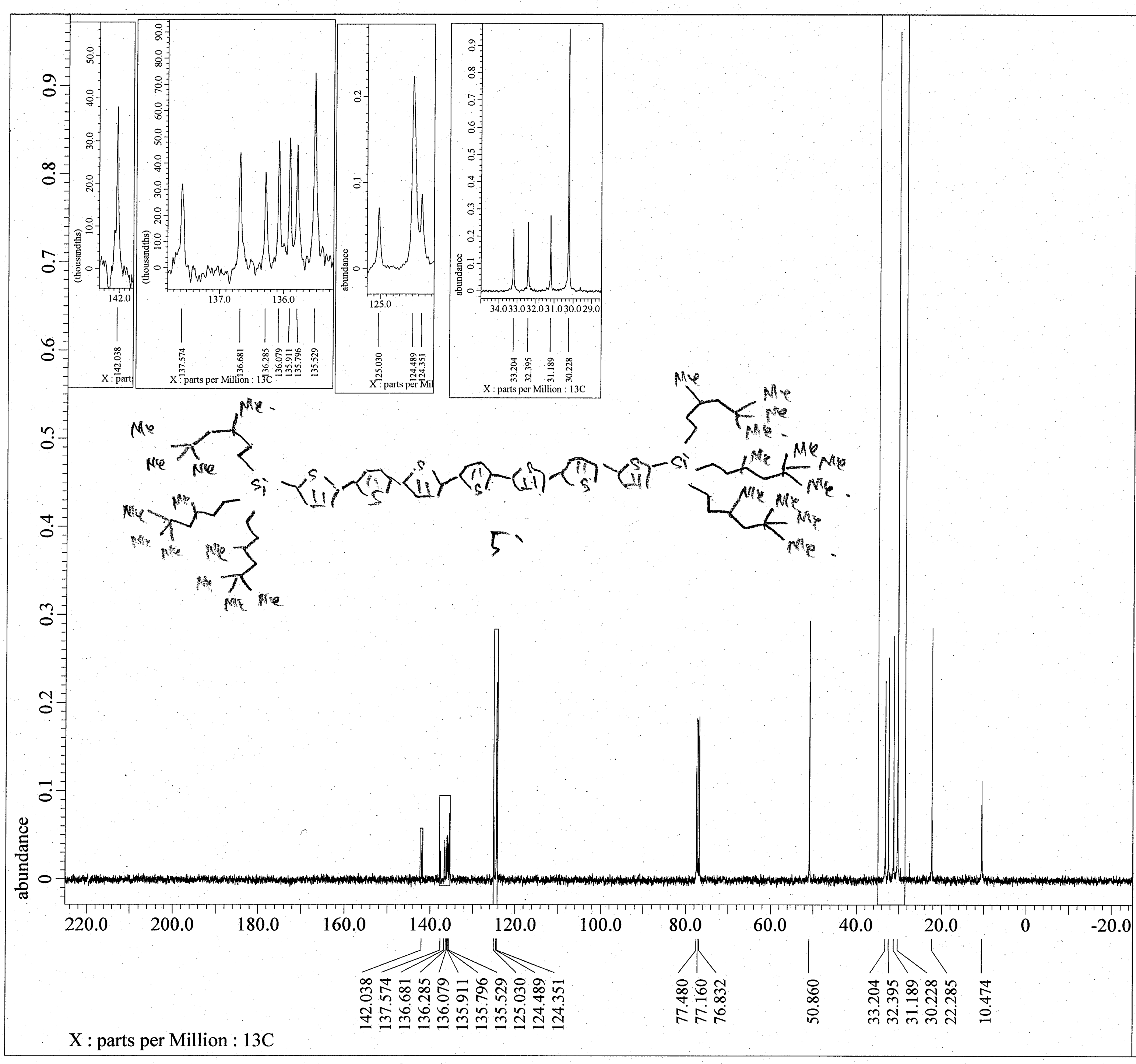


```
---- PROCESSING PARAMETERS ----
dc_balance( 0, FALSE )
sexp( 2.0[Hz], 0.0[s] )
trapezoid3( 0[%], 80[%], 100[%] )
zerofill( 1 )
fft( 1, TRUE, TRUE )
machinephase
ppm

Derived from: KND_298_13C-1.jdf

Filename            = KND_298_13C-2.jdf
Author              = element
Experiment          = single_pulse_dec
Sample_Id           = 1
Solvent             = CHLOROFORM-D
Actual_Start_Time   = 20-AUG-2022 03:04:37
Revision_Time       =  8-OCT-2022 11:33:40

Comment             = single pulse decoupled ga
Data_Format         = 1D COMPLEX
Dim_Size            = 32768
X_Domain            = 13C
Dim_Title           = 13C
Dim_Units           = [ppm]
Dimensions          = X
Site                = ECS 400
Spectrometer        = JNM-ECS400

Field_Strength      = 9.20197068[T] (390[MHz])
X_Acq_Duration      = 1.3303808[s]
X_Domain            = 13C
X_Freq              = 98.51479726[MHz]
X_Offset            = 100[ppm]
X_Points            = 32768
X_Prescans          = 4
X_Resolution        = 0.75166449[Hz]
X_Sweep             = 24.63054187[kHz]
Irr_Domain          = 1H
Irr_Freq            = 391.78655441[MHz]
Irr_Offset          = 5[ppm]
Clipped             = FALSE
Scans               = 49
Total_Scans         = 49

Relaxation_Delay    = 2[s]
Recvr_Gain          = 50
Temp_Get            = 21.6[dC]
X_90_Width          = 11[us]
X_Acq_Time          = 1.3303808[s]
X_Angle             = 30[deg]
X_Atn               = 4.9[dB]
X_Pulse             = 3.66666667[us]
Irr_Atn_Dec         = 22.05[dB]
Irr_Atn_Noe         = 22.05[dB]
Irr_Noise           = WALTZ
Decoupling          = TRUE
Initial_Wait        = 1[s]
Noe                 = TRUE
Noe_Time            = 2[s]
Repetition_Time     = 3.3303808[s]
```

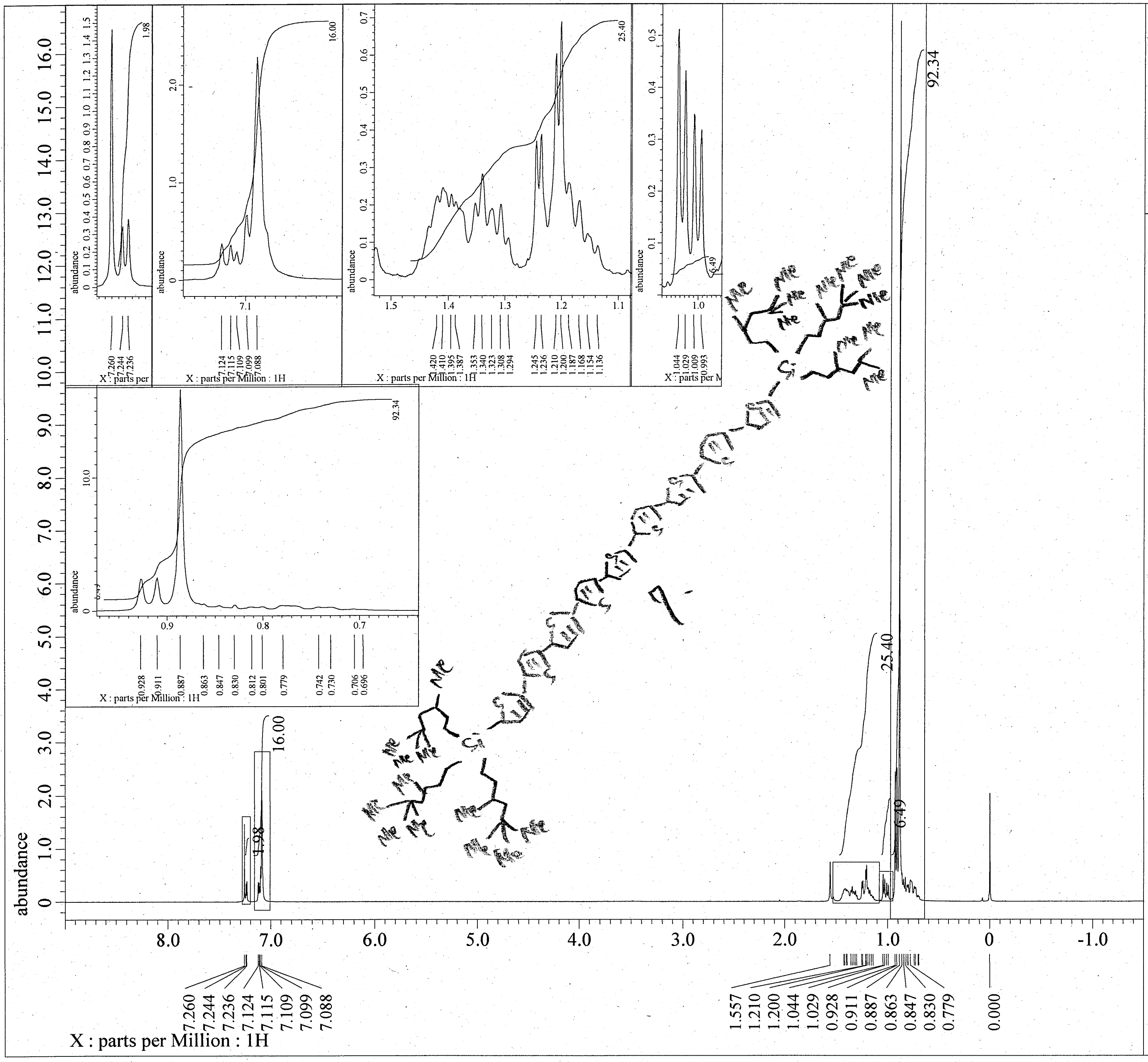

---- PROCESSING PARAMETERS ----
dc_balance(0, FALSE)
sexp(0.2[Hz], 0.0[s])
trapezoid3(0[%], 80[%], 100[%])
zerofill(1)
fft(1, TRUE, TRUE)
machinephase
ppm

Derived from: KND_272_pure3-1.jdf

Filename	=	KND_272_pure3-2.jdf
Author	=	element
Experiment	=	single_pulse.ex2
Sample_Id	=	S#345654
Solvent	=	CHLOROFORM-D
Actual_Start_Time	=	28-OCT-2022 16:19:19
Revision_Time	=	7-SEP-2023 18:51:44
Comment	=	single_pulse
Data_Format	=	1D COMPLEX
Dim_Size	=	13107
X_Domain	=	1H
Dim_Title	=	1H
Dim_Units	=	[ppm]
Dimensions	=	X
Site	=	ECS 400
Spectrometer	=	JNM-ECS400
Field_Strength	=	9.20197068[T] (390[MHz])
X_Acq_Duration	=	2.228224[s]
X_Domain	=	1H
X_Freq	=	391.78655441[MHz]
X_Offset	=	5[ppm]
X_Points	=	16384
X_Prescans	=	1
X_Resolution	=	0.44878791[Hz]
X_Sweep	=	7.35294118[kHz]
Irr_Domain	=	1H
Irr_Freq	=	391.78655441[MHz]
Irr_Offset	=	5[ppm]
Tri_Domain	=	1H
Tri_Freq	=	391.78655441[MHz]
Tri_Offset	=	5[ppm]
Clipped	=	FALSE
Scans	=	8
Total_Scans	=	8
Relaxation_Delay	=	5[s]
Recvr_Gain	=	44
Temp_Get	=	18.1[dC]
X_90_Width	=	10.8[us]
X_Acq_Time	=	2.228224[s]
X_Angle	=	45[deg]
X_Atn	=	1.9[dB]
X_Pulse	=	5.4[us]
Irr_Mode	=	Off
Tri_Mode	=	Off
Dante_Presat	=	FALSE
Initial_Wait	=	1[s]
Repetition_Time	=	7.228224[s]

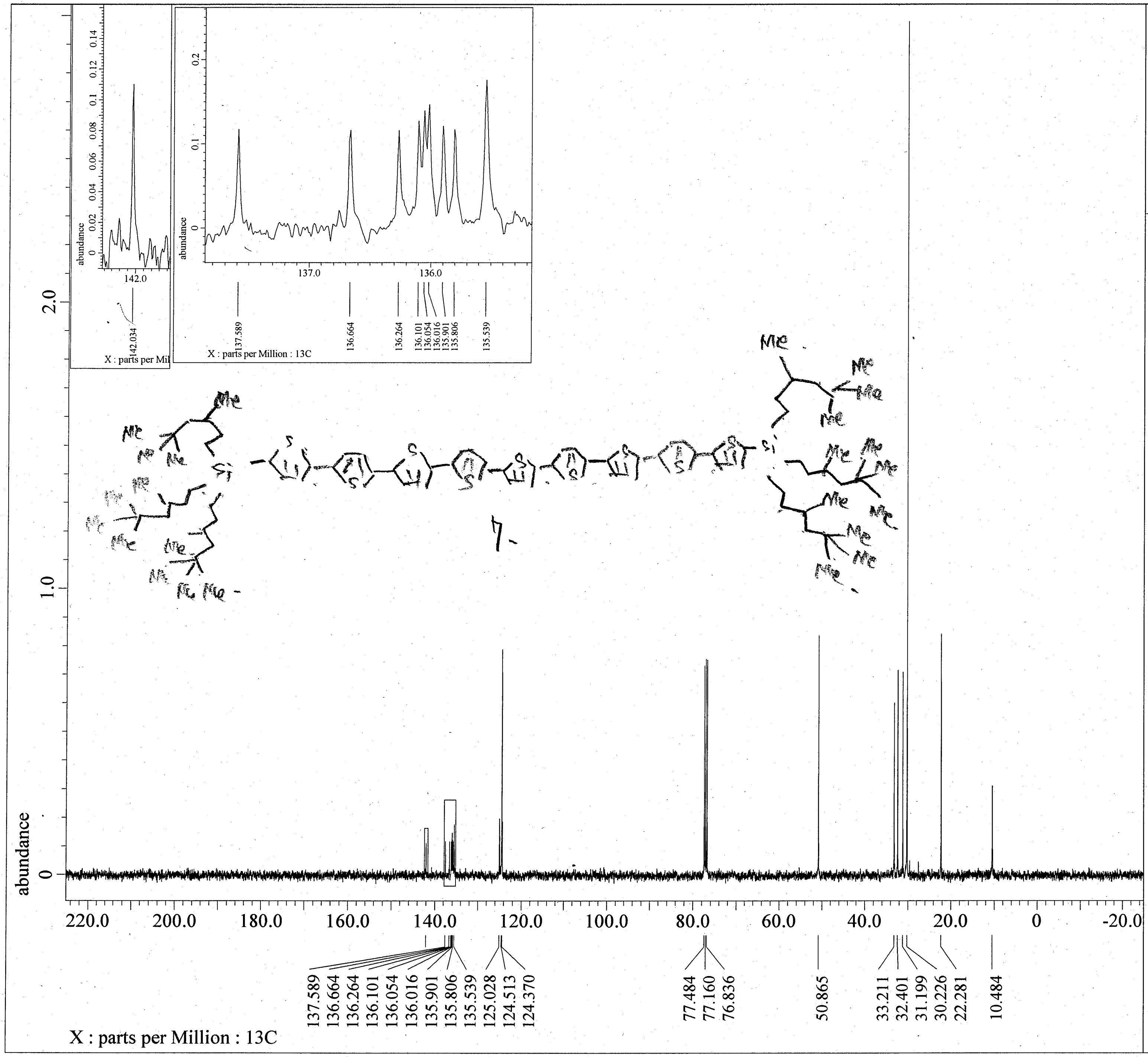

---- PROCESSING PARAMETERS ----
dc_balance(0, FALSE)
sexp(2.0[Hz], 0.0[s])
trapezoid3(0[%], 80[%], 100[%])
zerofill(1)
fft(1, TRUE, TRUE)
machinephase
ppm

Derived from: KND_272_13C-1.jdf

Filename	= KND_272_13C-2.jdf
Author	= element
Experiment	= single_pulse_dec
Sample_Id	= 1
Solvent	= CHLOROFORM-D
Actual_Start_Time	= 21-AUG-2022 01:56:37
Revision_Time	= 28-OCT-2022 09:56:00
Comment	= single pulse decoupled ga
Data_Format	= 1D COMPLEX
Dim_Size	= 26214
X_Domain	= 13C
Dim_Title	= 13C
Dim_Units	= [ppm]
Dimensions	= X
Site	= ECS 400
Spectrometer	= JNM-ECS400
Field_Strength	= 9.20197068[T] (390[MHz])
X_Acq_Duration	= 1.06430464[s]
X_Domain	= 13C
X_Freq	= 98.51479726[MHz]
X_Offset	= 100[ppm]
X_Points	= 32768
X_Prescans	= 4
X_Resolution	= 0.93958061[Hz]
X_Sweep	= 30.78817734[kHz]
Irr_Domain	= 1H
Irr_Freq	= 391.78655441[MHz]
Irr_Offset	= 5[ppm]
Clipped	= FALSE
Scans	= 66
Total_Scans	= 66
Relaxation_Delay	= 2[s]
Recvr_Gain	= 60
Temp_Get	= 21.5[dC]
X_90_Width	= 11[us]
X_Acq_Time	= 1.06430464[s]
X_Angle	= 30[deg]
X_Atn	= 4.9[dB]
X_Pulse	= 3.66666667[us]
Irr_Atn_Dec	= 22.05[dB]
Irr_Atn_Noe	= 22.05[dB]
Irr_Noise	= WALTZ
Decoupling	= TRUE
Initial_Wait	= 1[s]
Noe	= TRUE
Noe_Time	= 2[s]
Repetition_Time	= 3.06430464[s]

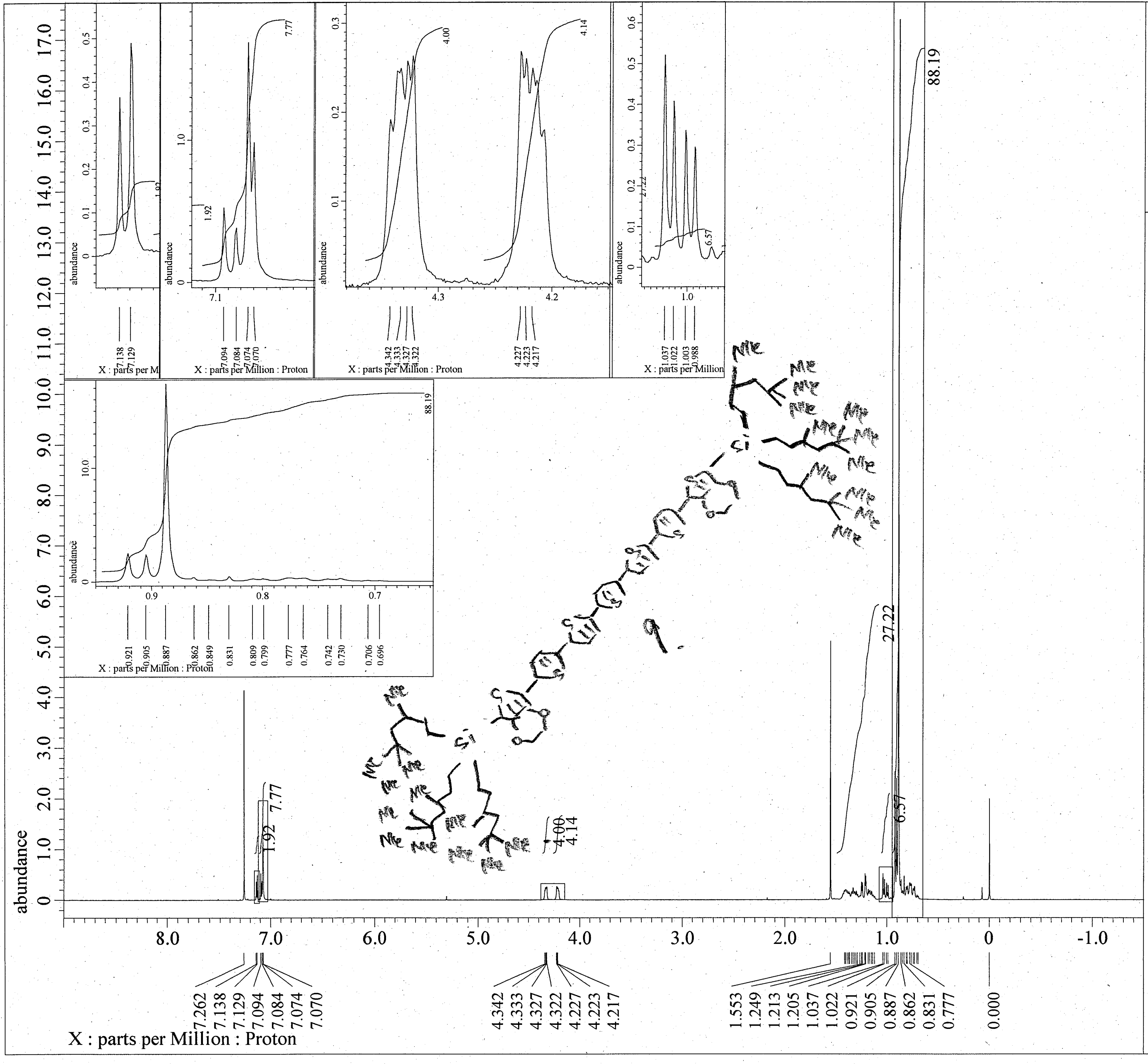


```
---- PROCESSING PARAMETERS ----
dc_balance( 0, FALSE )
sexp( 0.2[Hz], 0.0[s] )
trapezoid( 0[%], 0[%], 80[%], 100[%] )
zerofill( 1 )
fft( 1, TRUE, TRUE )
machinephase
ppm

Derived from: KND_296_pure_Proton-1-1.jdf

Filename            = KND_296_pure_Proton-1-2.j
Author              = element
Experiment          = proton.jxp
Sample_Id           = KND_296_pure
Solvent             = CHLOROFORM-D
Actual_Start_Time   = 23-MAR-2022 11:41:40
Revision_Time       =  7-SEP-2023 19:00:08

Comment             = single_pulse
Data_Format         = 1D COMPLEX
Dim_Size            = 13107
X_Domain            = Proton
Dim_Title           = Proton
Dim_Units           = [ppm]
Dimensions          = X
Spectrometer        = DELTA2_NMR

Field_Strength      = 9.4073814[T] (400[MHz])
X_Acq_Duration      = 2.18103808[s]
X_Domain            = 1H
X_Freq              = 400.53219825[MHz]
X_Offset            = 5[ppm]
X_Points            = 16384
X_Prescans          = 1
X_Resolution        = 0.45849727[Hz]
X_Sweep             = 7.51201923[kHz]
X_Sweep_Clipped     = 6.00961538[kHz]
Irr_Domain          = Proton
Irr_Freq            = 400.53219825[MHz]
Irr_Offset          = 5[ppm]
Tri_Domain          = Proton
Tri_Freq            = 400.53219825[MHz]
Tri_Offset          = 5[ppm]
Clipped             = FALSE
Scans               = 8
Total_Scans         = 8

Relaxation_Delay    = 5[s]
Recvr_Gain          = 50
Temp_Get            = 18.7[dC]
X_90_Width          = 6[us]
X_Acq_Time          = 2.18103808[s]
X_Angle             = 45[deg]
X_Atn               = 0.8[dB]
X_Pulse             = 3[us]
Irr_Mode            = Off
Tri_Mode            = Off
Dante_Presat        = FALSE
Initial_Wait        = 1[s]
Repetition_Time     = 7.18103808[s]
```

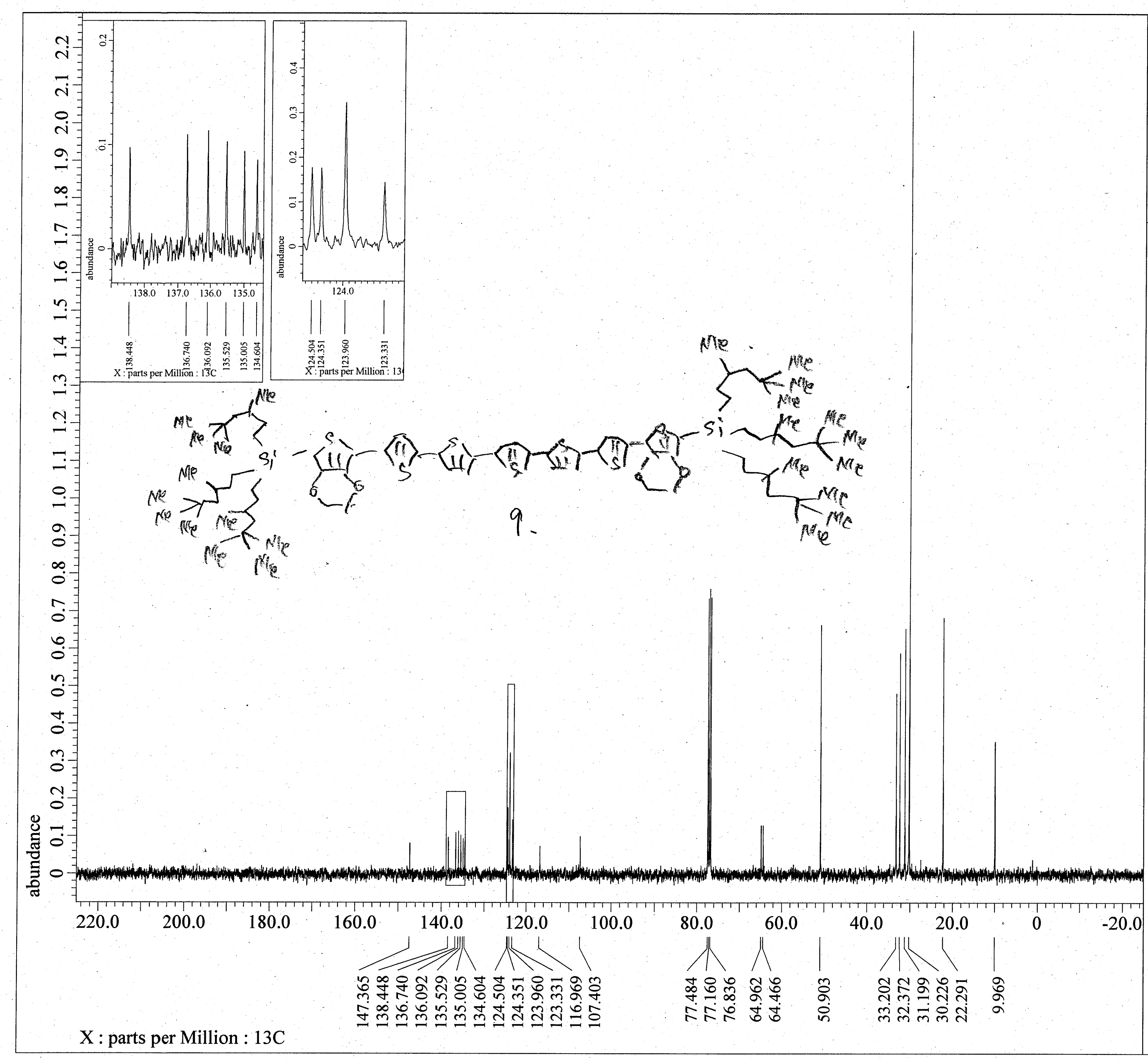

---- PROCESSING PARAMETERS ----
dc_balance(0, FALSE)
sexp(2.0[Hz], 0.0[s])
trapezoid3(0[%], 80[%], 100[%])
zerofill(1)
fft(1, TRUE, TRUE)
machinephase
ppm

Derived from: KND_296_13C-1.jdf

Filename	= KND_296_13C-2.jdf
Author	= element
Experiment	= single_pulse_dec
Sample_Id	= 1
Solvent	= CHLOROFORM-D
Actual_Start_Time	= 21-AUG-2022 02:20:31
Revision_Time	= 8-OCT-2022 15:43:30
Comment	= single pulse decoupled ga
Data_Format	= 1D COMPLEX
Dim_Size	= 26214
X_Domain	= 13C
Dim_Title	= 13C
Dim_Units	= [ppm]
Dimensions	= X
Site	= ECS 400
Spectrometer	= JNM-ECS400
Field_Strength	= 9.20197068[T] (390[MHz])
X_Acq_Duration	= 1.06430464[s]
X_Domain	= 13C
X_Freq	= 98.51479726[MHz]
X_Offset	= 100[ppm]
X_Points	= 32768
X_Prescans	= 4
X_Resolution	= 0.93958061[Hz]
X_Sweep	= 30.78817734[kHz]
Irr_Domain	= 1H
Irr_Freq	= 391.78655441[MHz]
Irr_Offset	= 5[ppm]
Clipped	= FALSE
Scans	= 64
Total_Scans	= 64
Relaxation_Delay	= 2[s]
Recvr_Gain	= 60
Temp_Get	= 21.6[dC]
X_90_Width	= 11[us]
X_Acq_Time	= 1.06430464[s]
X_Angle	= 30[deg]
X_Atn	= 4.9[dB]
X_Pulse	= 3.66666667[us]
Irr_Atn_Dec	= 22.05[dB]
Irr_Atn_Noe	= 22.05[dB]
Irr_Noise	= WALTZ
Decoupling	= TRUE
Initial_Wait	= 1[s]
Noe	= TRUE
Noe_Time	= 2[s]
Repetition_Time	= 3.06430464[s]

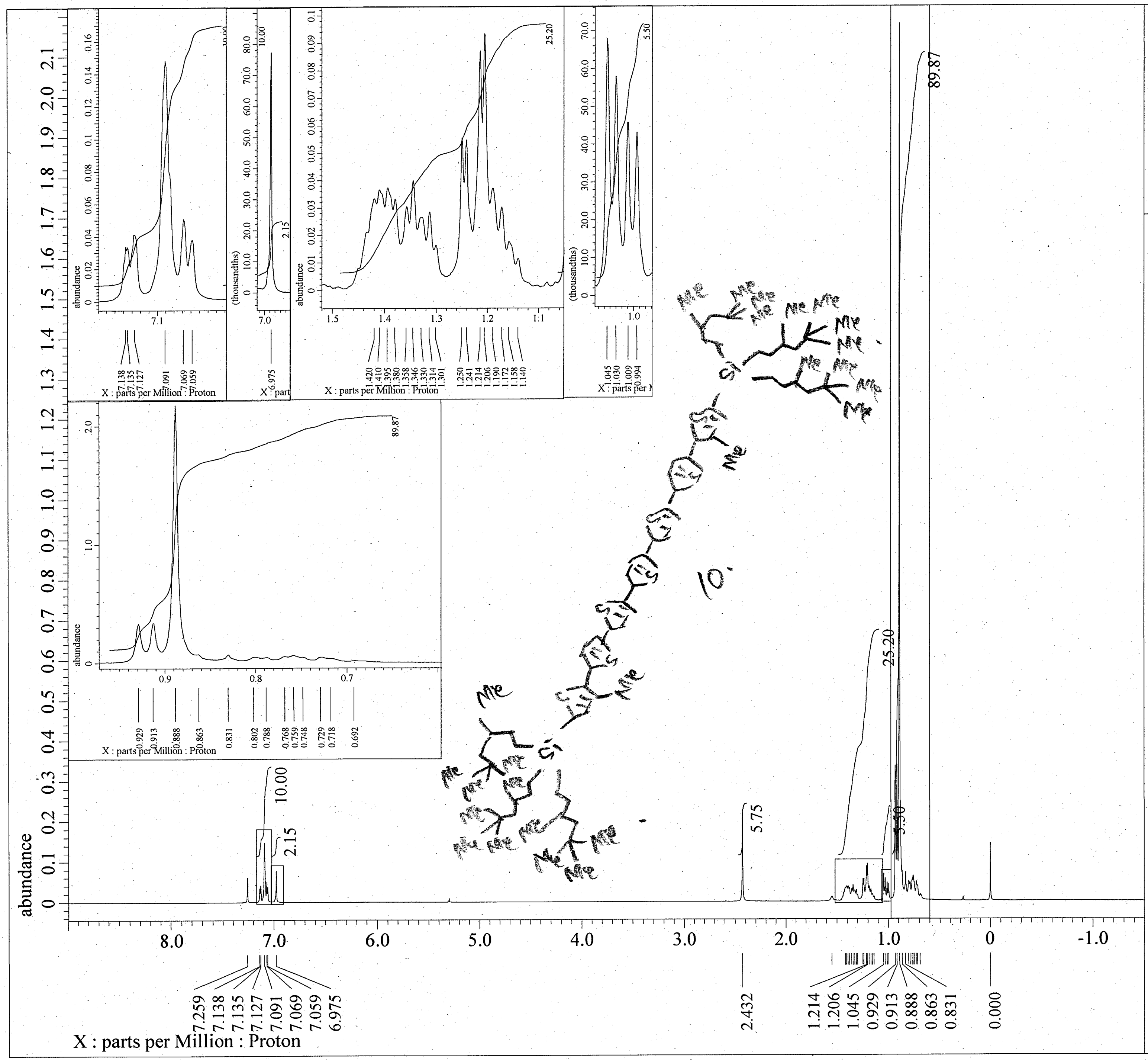


```
---- PROCESSING PARAMETERS ----
sexp( 0.2[Hz], 0.0[s] )
trapezoid( 0[%], 0[%], 80[%], 100[%] )
zerofill( 1 )
fft( 1, TRUE, TRUE )
machinephase
ppm

Derived from: KND297pure_Proton-1-1.jdf
```

```
Filename            = KND297pure_Proton-1-2
Author              = element
Experiment          = proton_auto.jxp
Sample_Id           = KND297pure
Solvent             = CHLOROFORM-D
Actual_Start_Time   =  7-NOV-2022 17:41:24
Revision_Time       =  7-SEP-2023 19:10:57

Comment             = single_pulse
Data_Format         = 1D COMPLEX
Dim_Size            = 13107
X_Domain            = Proton
Dim_Title           = Proton
Dim_Units           = [ppm]
Dimensions          = X
Spectrometer        = DELTA2_NMR

Field_Strength      = 9.2982153[T] (400[MHz
X_Acq_Duration      = 2.20725248[s]
X_Domain            = Proton
X_Freq              = 395.88430144[MHz]
X_Offset            = 5[ppm]
X_Points            = 16384
X_Prescans          = 1
X_Resolution        = 0.45305193[Hz]
X_Sweep             = 7.42280285[kHz]
X_Sweep_Clipped     = 5.93824228[kHz]
Irr_Domain          = Proton
Irr_Freq            = 395.88430144[MHz]
Irr_Offset          = 5[ppm]
Tri_Domain          = Proton
Tri_Freq            = 395.88430144[MHz]
Tri_Offset          = 5[ppm]
Blanking            = 2[us]
Clipped             = FALSE
Scans               = 8
Total_Scans         = 8

Relaxation_Delay    = 5[s]
Recvr_Gain          = 46
Temp_Get            = 18.4[dC]
X_90_Width          = 9.5[us]
X_Acq_Time          = 2.20725248[s]
X_Angle             = 45[deg]
X_Atn               = 3.4[dB]
X_Pulse             = 4.75[us]
Irr_Mode            = Off
Tri_Mode            = Off
Dante_Loop          = 500
Dante_Presat        = FALSE
Decimation_Rate     = 0
Experiment_Path     = C:\Program Files\JEOL
Initial_Wait        = 1[s]
Phase               = {0, 90, 270, 180, 180
Presat_Time         = 5[s]
```

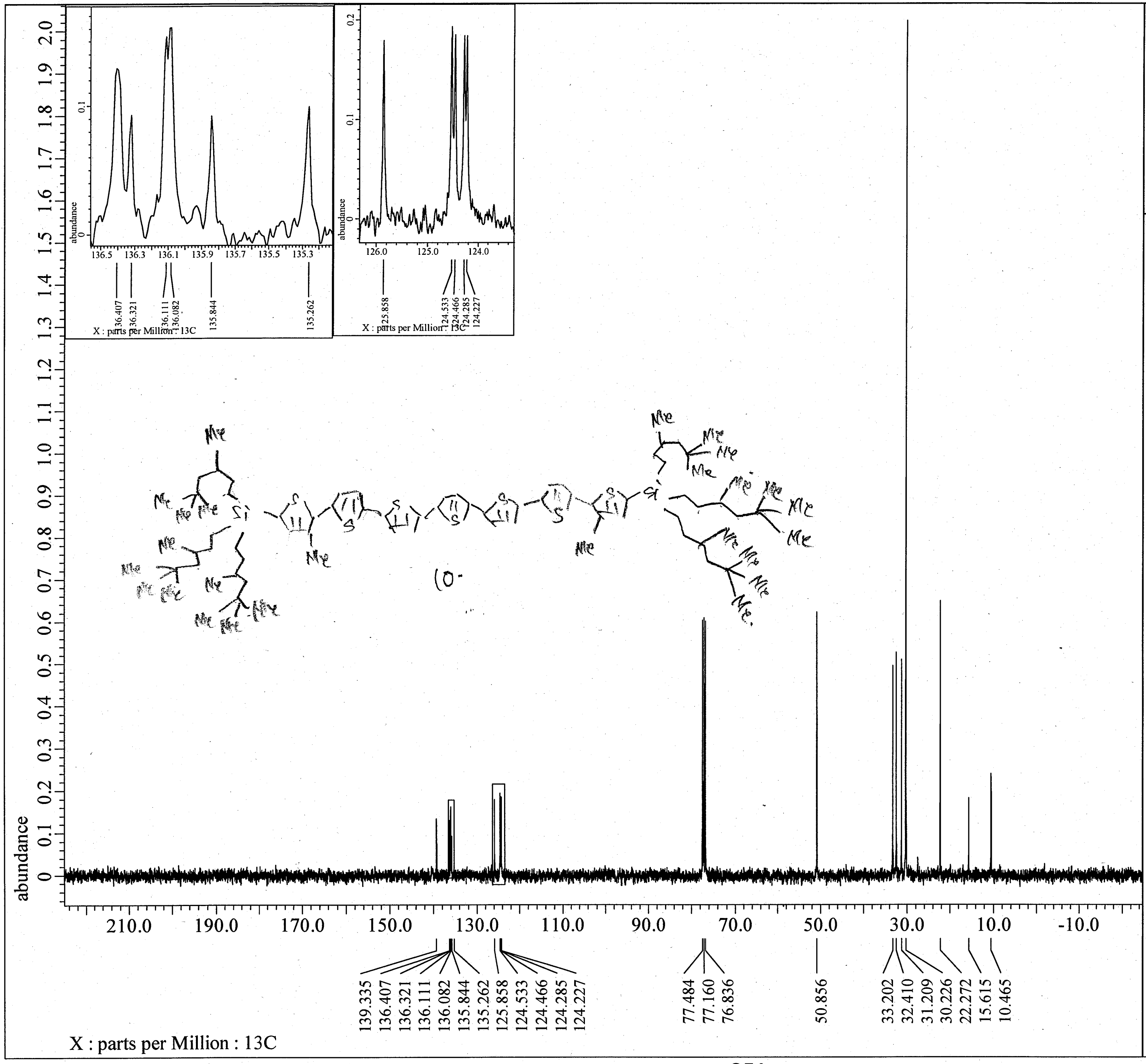


```
---- PROCESSING PARAMETERS ----
dc_balance( 0, FALSE )
sexp( 2.0[Hz], 0.0[s] )
trapezoid3( 0[%], 80[%], 100[%] )
zerofill( 1 )
fft( 1, TRUE, TRUE )
machinephase
ppm

Derived from: KND_297_13C-1.jdf
```

```
Filename            = KND_297_13C-2.jdf
Author              = element
Experiment          = single_pulse_dec
Sample_Id           = 1
Solvent             = CHLOROFORM-D
Actual_Start_Time   = 26-AUG-2022 01:48:39
Revision_Time       = 24-NOV-2022 14:00:57

Comment             = single pulse decoupled ga
Data_Format         = 1D COMPLEX
Dim_Size            = 26214
X_Domain            = 13C
Dim_Title           = 13C
Dim_Units           = [ppm]
Dimensions          = X
Site                = ECS 400
Spectrometer        = JNM-ECS400

Field_Strength      = 9.20197068[T] (390[MHz])
X_Acq_Duration      = 1.06430464[s]
X_Domain            = 13C
X_Freq              = 98.51479726[MHz]
X_Offset            = 100[ppm]
X_Points            = 32768
X_Prescans          = 4
X_Resolution        = 0.93958061[Hz]
X_Sweep             = 30.78817734[kHz]
Irr_Domain          = 1H
Irr_Freq            = 391.78655441[MHz]
Irr_Offset          = 5[ppm]
Clipped             = FALSE
Scans               = 77
Total_Scans         = 77

Relaxation_Delay    = 2[s]
Recvr_Gain          = 60
Temp_Get            = 21.5[dC]
X_90_Width          = 11[us]
X_Acq_Time          = 1.06430464[s]
X_Angle             = 30[deg]
X_Atn               = 4.9[dB]
X_Pulse             = 3.66666667[us]
Irr_Atn_Dec         = 22.05[dB]
Irr_Atn_Noe         = 22.05[dB]
Irr_Noise           = WALTZ
Decoupling          = TRUE
Initial_Wait        = 1[s]
Noe                 = TRUE
Noe_Time            = 2[s]
Repetition_Time     = 3.06430464[s]
```

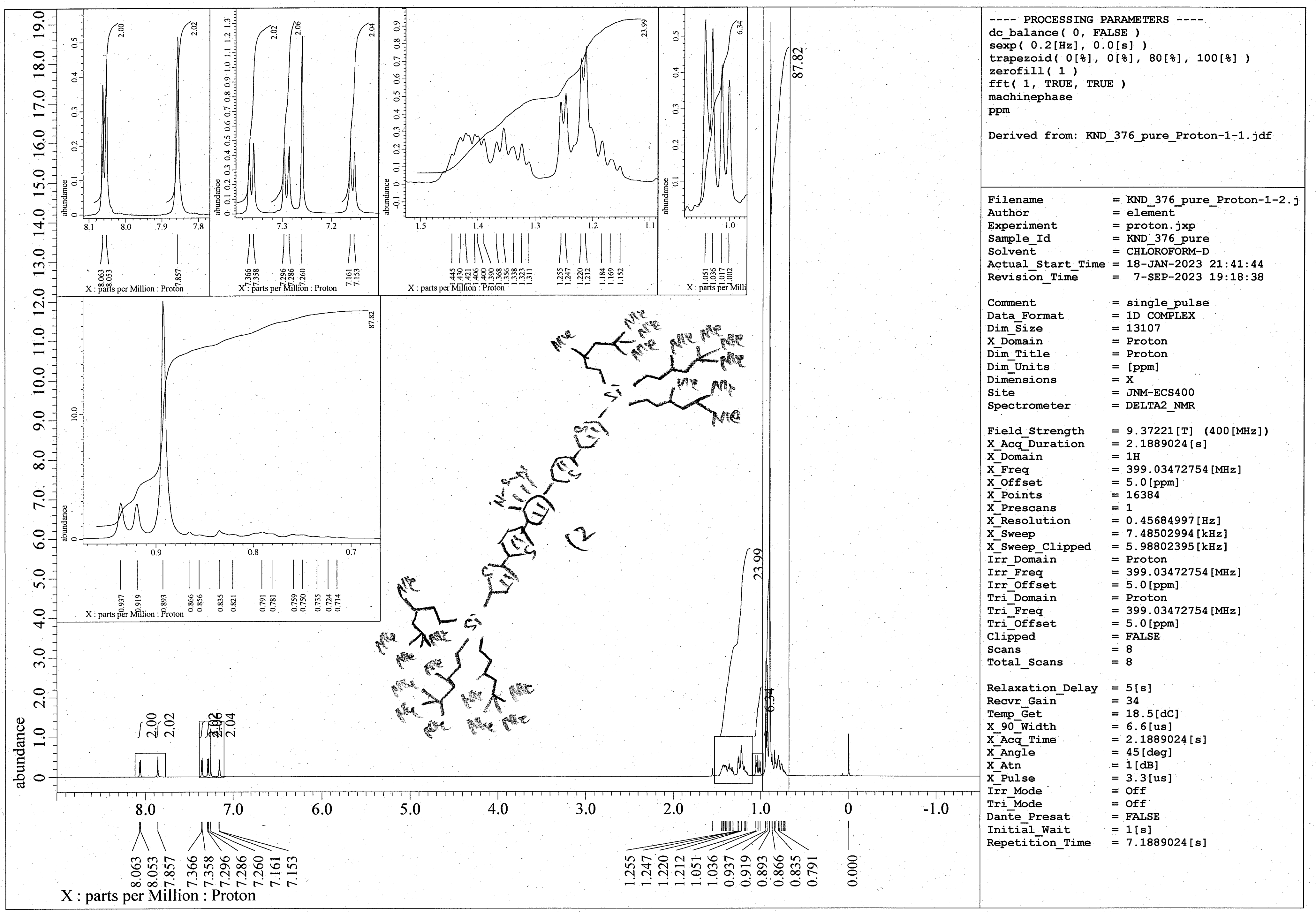

---- PROCESSING PARAMETERS ----
dc_balance(0, FALSE)
sexp(0.2[Hz], 0.0[s])
trapezoid(0[%], 0[%], 80[%], 100[%])
zerofill(1)
fft(1, TRUE, TRUE)
machinephase
ppm
Derived from: KND_376_pure_Proton-1-1.jdf
Filename = KND_376_pure_Proton-1-2.j
Author = element
Experiment = proton.jxp
Sample_Id = KND_376_pure
Solvent = CHLOROFORM-D
Actual_Start_Time = 18-JAN-2023 21:41:44
Revision_Time = 7-SEP-2023 19:18:38
Comment = single_pulse
Data_Format = 1D COMPLEX
Dim_Size = 13107
X_Domain = Proton
Dim_Title = Proton
Dim_Units = [ppm]
Dimensions = X
Site = JNM-ECS400
Spectrometer = DELTA2_NMR
Field_Strength = 9.37221[T] (400[MHz])
X_Acq_Duration = 2.1889024[s]
X_Domain = 1H
X_Freq = 399.03472754[MHz]
X_Offset = 5.0[ppm]
X_Points = 16384
X_Prescans = 1
X_Resolution = 0.45684997[Hz]
X_Sweep = 7.48502994[kHz]
X_Sweep_Clipped = 5.98802395[kHz]
Irr_Domain = Proton
Irr_Freq = 399.03472754[MHz]
Irr_Offset = 5.0[ppm]
Tri_Domain = Proton
Tri_Freq = 399.03472754[MHz]
Tri_Offset = 5.0[ppm]
Clipped = FALSE
Scans = 8
Total_Scans = 8
Relaxation_Delay = 5[s]
Recvr_Gain = 34
Temp_Get = 18.5[dC]
X_90_Width = 6.6[us]
X_Acq_Time = 2.1889024[s]
X_Angle = 45[deg]
X_Atn = 1[dB]
X_Pulse = 3.3[us]
Irr_Mode = Off
Tri_Mode = Off
Dante_Presat = FALSE
Initial_Wait = 1[s]
Repetition_Time = 7.1889024[s]
abundance
X : parts per Million : Proton
8.063 8.053 7.857 7.366 7.358 7.296 7.286 7.260 7.161 7.153
1.255 1.247 1.220 1.212 1.051 1.036 0.937 0.919 0.893 0.866 0.835 0.791 0.000
2.00 2.02 2.02 2.06 2.04 23.99 6.34 87.82

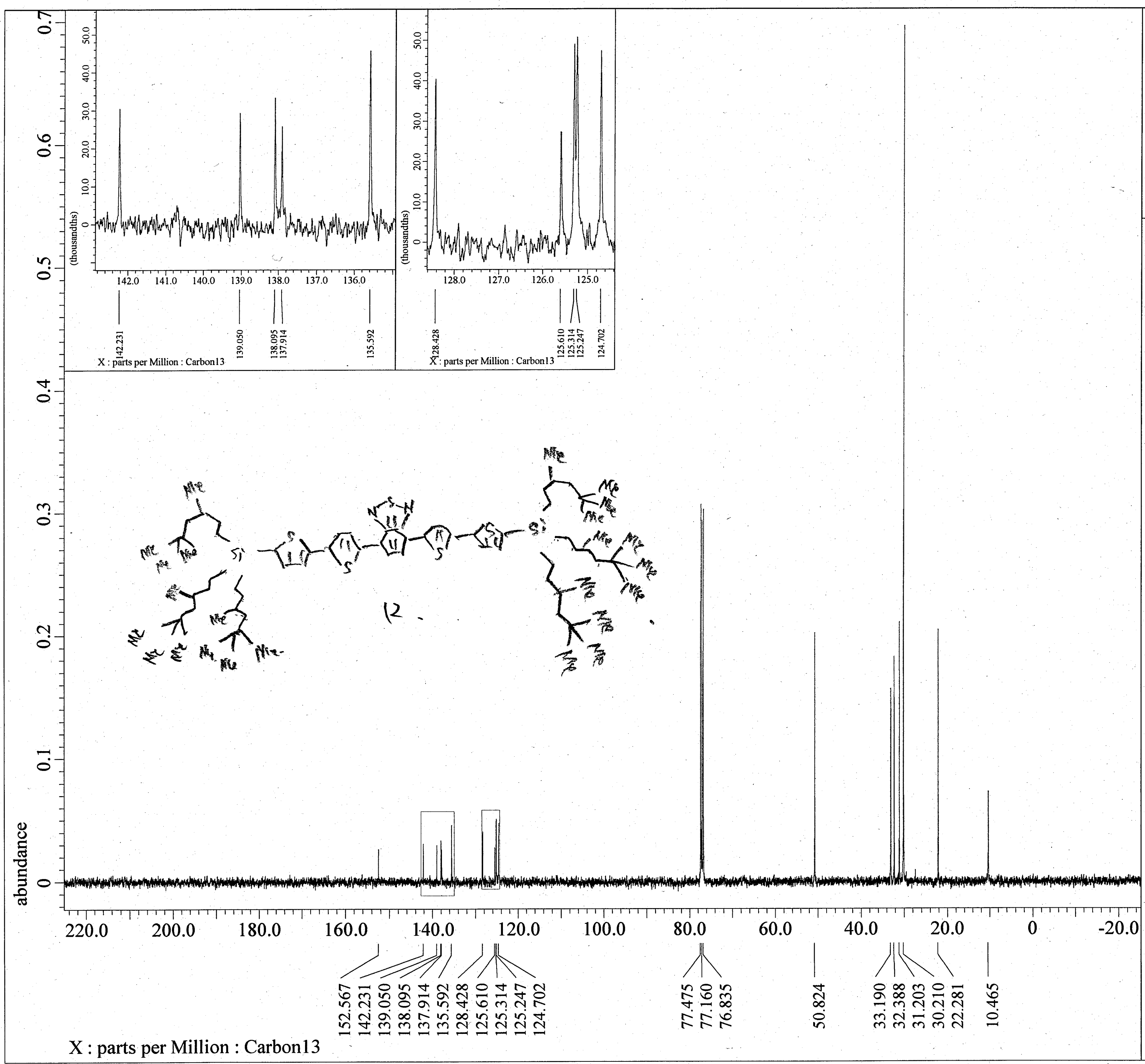


```
---- PROCESSING PARAMETERS ----
dc_balance( 0, FALSE )
sexp( 2.0[Hz], 0.0[s] )
trapezoid( 0[%], 0[%], 80[%], 100[%] )
zerofill( 1 )
fft( 1, TRUE, TRUE )
machinephase
ppm

Derived from: KND_376_13C_Carbon-1-1.jdf
```

```
Filename            = KND_376_13C_Carbon-1-2.jd
Author              = element
Experiment          = carbon.jxp
Sample_Id           = KND_376_13C
Solvent             = CHLOROFORM-D
Actual_Start_Time   = 18-JAN-2023 21:58:02
Revision_Time       = 20-JAN-2023 20:29:26

Comment             = single pulse decoupled ga
Data_Format         = 1D COMPLEX
Dim_Size            = 26214
X_Domain            = Carbon
Dim_Title           = Carbon13
Dim_Units           = [ppm]
Dimensions          = X
Site                = JNM-ECS400
Spectrometer        = DELTA2_NMR

Field_Strength      = 9.37221[T] (400[MHz])
X_Acq_Duration      = 1.04333312[s]
X_Domain            = 13C
X_Freq              = 100.33735165[MHz]
X_Offset            = 100.0[ppm]
X_Points            = 32768
X_Prescans          = 4
X_Resolution        = 0.95846665[Hz]
X_Sweep             = 31.40703518[kHz]
X_Sweep_Clipped     = 25.12562814[kHz]
Irr_Domain          = Proton
Irr_Freq            = 399.03472754[MHz]
Irr_Offset          = 5.0[ppm]
Clipped             = FALSE
Scans               = 150
Total_Scans         = 150

Relaxation_Delay    = 2[s]
Recvr_Gain          = 50
Temp_Get            = 18.1[dC]
X_90_Width          = 10.9[us]
X_Acq_Time          = 1.04333312[s]
X_Angle             = 30[deg]
X_Atn               = 5.4[dB]
X_Pulse             = 3.63333333[us]
Irr_Atn_Dec         = 25.823[dB]
Irr_Atn_Noe         = 25.823[dB]
Irr_Noise           = WALTZ
Irr_Pwidth          = 0.115[ms]
Decoupling          = TRUE
Initial_Wait        = 1[s]
Noe                 = TRUE
Noe_Time            = 2[s]
Repetition_Time     = 3.04333312[s]
```

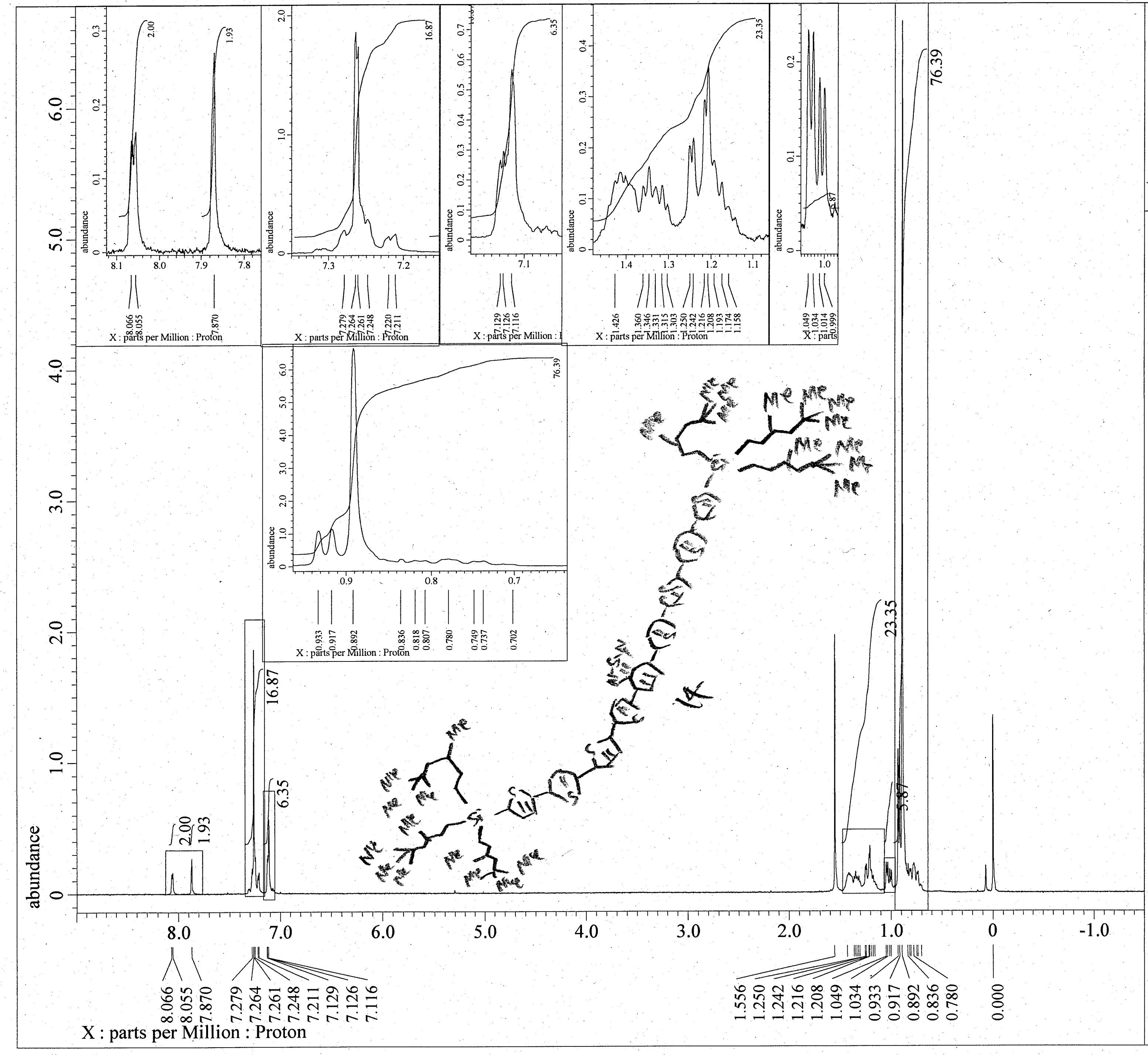


```
---- PROCESSING PARAMETERS ----
dc_balance( 0, FALSE )
sexp( 0.2[Hz], 0.0[s] )
trapezoid( 0[%], 0[%], 80[%], 100[%] )
zerofill( 1 )
fft( 1, TRUE, TRUE )
machinephase
ppm

Derived from: KND_281_pure3_Proton-1-1.jdf

Filename            = KND_281_pure3_Proton-1-2.
Author              = element
Experiment          = proton.jxp
Sample_Id           = KND_281_pure3
Solvent             = CHLOROFORM-D
Actual_Start_Time   = 19-MAR-2022 17:08:21
Revision_Time       =  7-SEP-2023 19:31:14

Comment             = single_pulse
Data_Format         = 1D COMPLEX
Dim_Size            = 13107
X_Domain            = Proton
Dim_Title           = Proton
Dim_Units           = [ppm]
Dimensions          = X
Spectrometer        = DELTA2_NMR

Field_Strength      = 9.4073814[T] (400[MHz])
X_Acq_Duration      = 2.18103808[s]
X_Domain            = 1H
X_Freq              = 400.53219825[MHz]
X_Offset            = 5[ppm]
X_Points            = 16384
X_Prescans          = 1
X_Resolution        = 0.45849727[Hz]
X_Sweep             = 7.51201923[kHz]
X_Sweep_Clipped     = 6.00961538[kHz]
Irr_Domain          = Proton
Irr_Freq            = 400.53219825[MHz]
Irr_Offset          = 5[ppm]
Tri_Domain          = Proton
Tri_Freq            = 400.53219825[MHz]
Tri_Offset          = 5[ppm]
Clipped             = FALSE
Scans               = 8
Total_Scans         = 8

Relaxation_Delay    = 5[s]
Recvr_Gain          = 48
Temp_Get            = 18.9[dC]
X_90_Width          = 6[us]
X_Acq_Time          = 2.18103808[s]
X_Angle             = 45[deg]
X_Atn               = 0.8[dB]
X_Pulse             = 3[us]
Irr_Mode            = Off
Tri_Mode            = Off
Dante_Presat        = FALSE
Initial_Wait        = 1[s]
Repetition_Time     = 7.18103808[s]
```

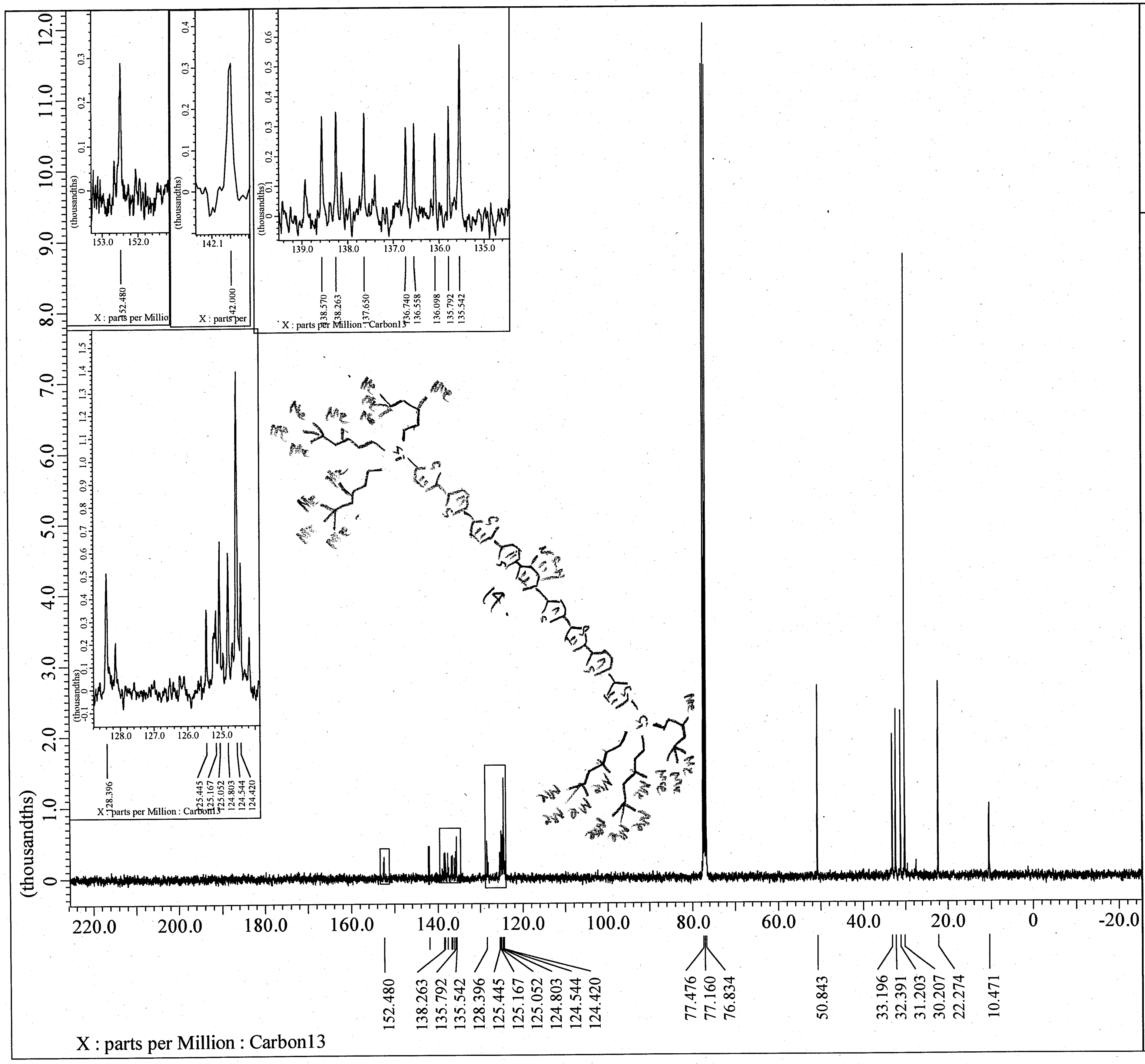

---- PROCESSING PARAMETERS ----
sexp(2.0[Hz], 0.0[s])
trapezoid(0[%], 0[%], 80[%], 100[%])
zerofill(1)
fft(1, TRUE, TRUE)
machinephase
ppm

Derived from: KND_281_13C_Carbon-1-1.jdf

Filename	= KND_281_13C_Carbon
Author	= element
Experiment	= carbon_auto.jxp
Sample_Id	= KND_281_13C
Solvent	= CHLOROFORM-D
Actual_Start_Time	= 19-MAR-2022 17:37:
Revision_Time	= 24-NOV-2022 15:55:
Comment	= single pulse decou
Data_Format	= 1D COMPLEX
Dim_Size	= 26214
X_Domain	= Carbon13
Dim_Title	= Carbon13
Dim_Units	= [ppm]
Dimensions	= X
Spectrometer	= DELTA2_NMR
Field_Strength	= 9.2982153[T] (400[
X_Acq_Duration	= 1.048576[s]
X_Domain	= Carbon13
X_Freq	= 99.54517646[MHz]
X_Offset	= 100[ppm]
X_Points	= 32768
X_Prescans	= 4
X_Resolution	= 0.95367432[Hz]
X_Sweep	= 31.25[kHz]
X_Sweep_Clipped	= 25[kHz]
Irr_Domain	= Proton
Irr_Freq	= 395.88430144[MHz]
Irr_Offset	= 5[ppm]
Blanking	= 5[us]
Clipped	= TRUE
Scans	= 1024
Total_Scans	= 1024
Relaxation_Delay	= 2[s]
Recvr_Gain	= 50
Temp_Get	= 19.1[dC]
X_90_Width	= 9.65[us]
X_Acq_Time	= 1.048576[s]
X_Angle	= 30[deg]
X_Atn	= 8[dB]
X_Pulse	= 3.21666667[us]
Irr_Atn_Dec	= 25.059[dB]
Irr_Atn_Dec_Calc	= 25.059[dB]
Irr_Atn_Dec_Default_Calc	= 25.059[dB]
Irr_Atn_Noe	= 25.059[dB]
Irr_Dec_Bandwidth_Hz	= 4.7826087[kHz]
Irr_Dec_Bandwidth_Ppm	= 12.08082432[ppm]
Irr_Dec_Freq	= 395.88430144[MHz]
Irr_Dec_Merit_Factor	= 2.2
Irr_Decoupling	= TRUE
Irr_Noe	= TRUE
Irr_Noise	= WALTZ
Irr_Offset_Default	= 5[ppm]

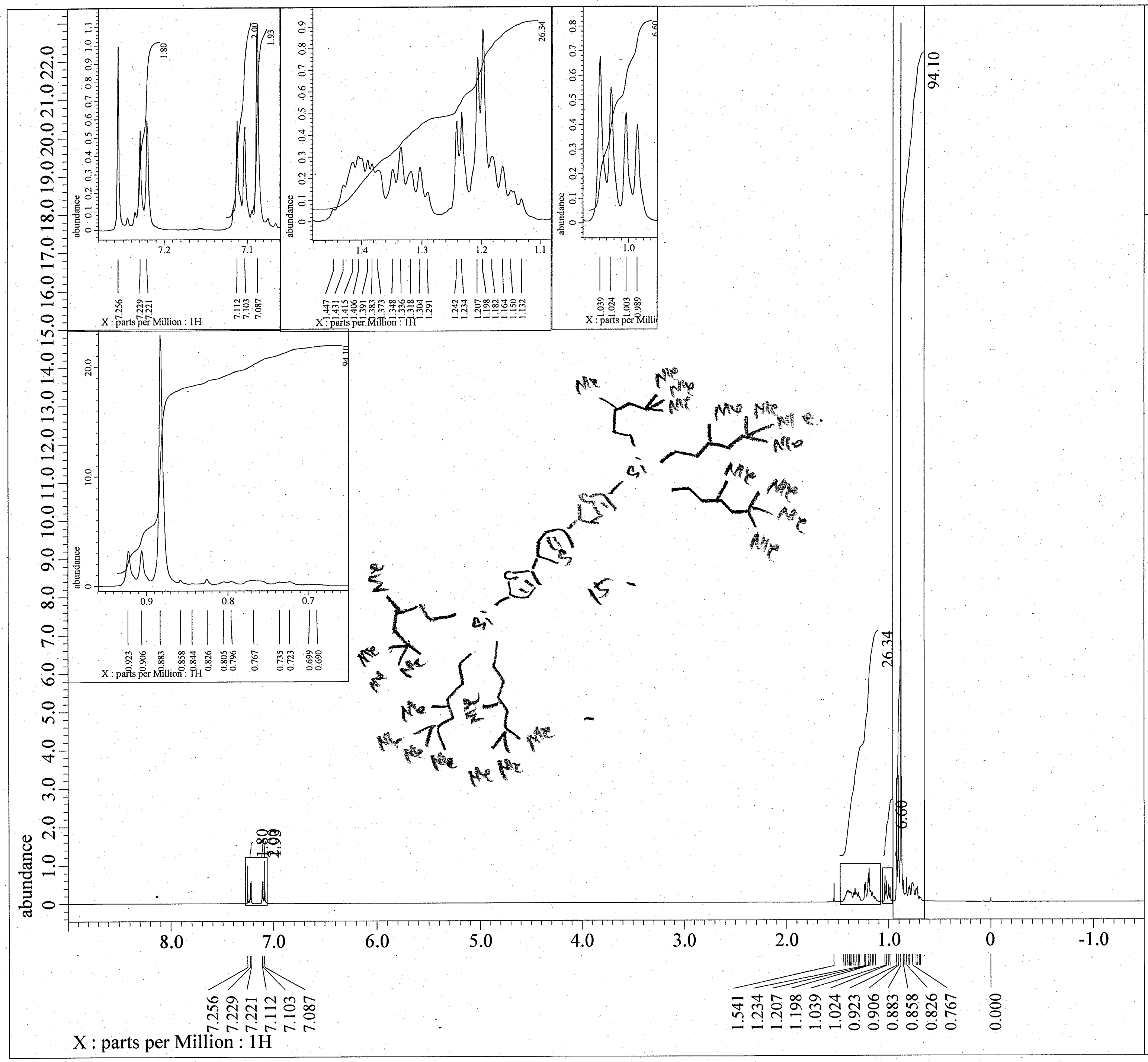


```
---- PROCESSING PARAMETERS ----
dc_balance( 0, FALSE )
sexp( 0.2[Hz], 0.0[s] )
trapezoid3( 0[%], 80[%], 100[%] )
zerofill( 1 )
fft( 1, TRUE, TRUE )
machinephase
ppm

Derived from: KND_214_pure_2-1.jdf
```

```
Filename            = KND_214_pure_2-2.jdf
Author              = element
Experiment          = single_pulse.ex2
Sample_Id           = S#468698
Solvent             = CHLOROFORM-D
Actual_Start_Time   = 27-JAN-2022 19:52:22
Revision_Time       =  7-SEP-2023 19:40:18

Comment             = single_pulse
Data_Format         = 1D COMPLEX
Dim_Size            = 13107
X_Domain            = 1H
Dim_Title           = 1H
Dim_Units           = [ppm]
Dimensions          = X
Site                = ECS 400
Spectrometer        = JNM-ECS400

Field_Strength      = 9.20197068[T] (390[MHz])
X_Acq_Duration      = 2.228224[s]
X_Domain            = 1H
X_Freq              = 391.78655441[MHz]
X_Offset            = 5[ppm]
X_Points            = 16384
X_Prescans          = 1
X_Resolution        = 0.44878791[Hz]
X_Sweep             = 7.35294118[kHz]
Irr_Domain          = 1H
Irr_Freq            = 391.78655441[MHz]
Irr_Offset          = 5[ppm]
Tri_Domain          = 1H
Tri_Freq            = 391.78655441[MHz]
Tri_Offset          = 5[ppm]
Clipped             = FALSE
Scans               = 8
Total_Scans         = 8

Relaxation_Delay    = 5[s]
Recvr_Gain          = 32
Temp_Get            = 19.6[dC]
X_90_Width          = 10.8[us]
X_Acq_Time          = 2.228224[s]
X_Angle             = 45[deg]
X_Atn               = 1.9[dB]
X_Pulse             = 5.4[us]
Irr_Mode            = Off
Tri_Mode            = Off
Dante_Presat        = FALSE
Initial_Wait        = 1[s]
Repetition_Time     = 7.228224[s]
```

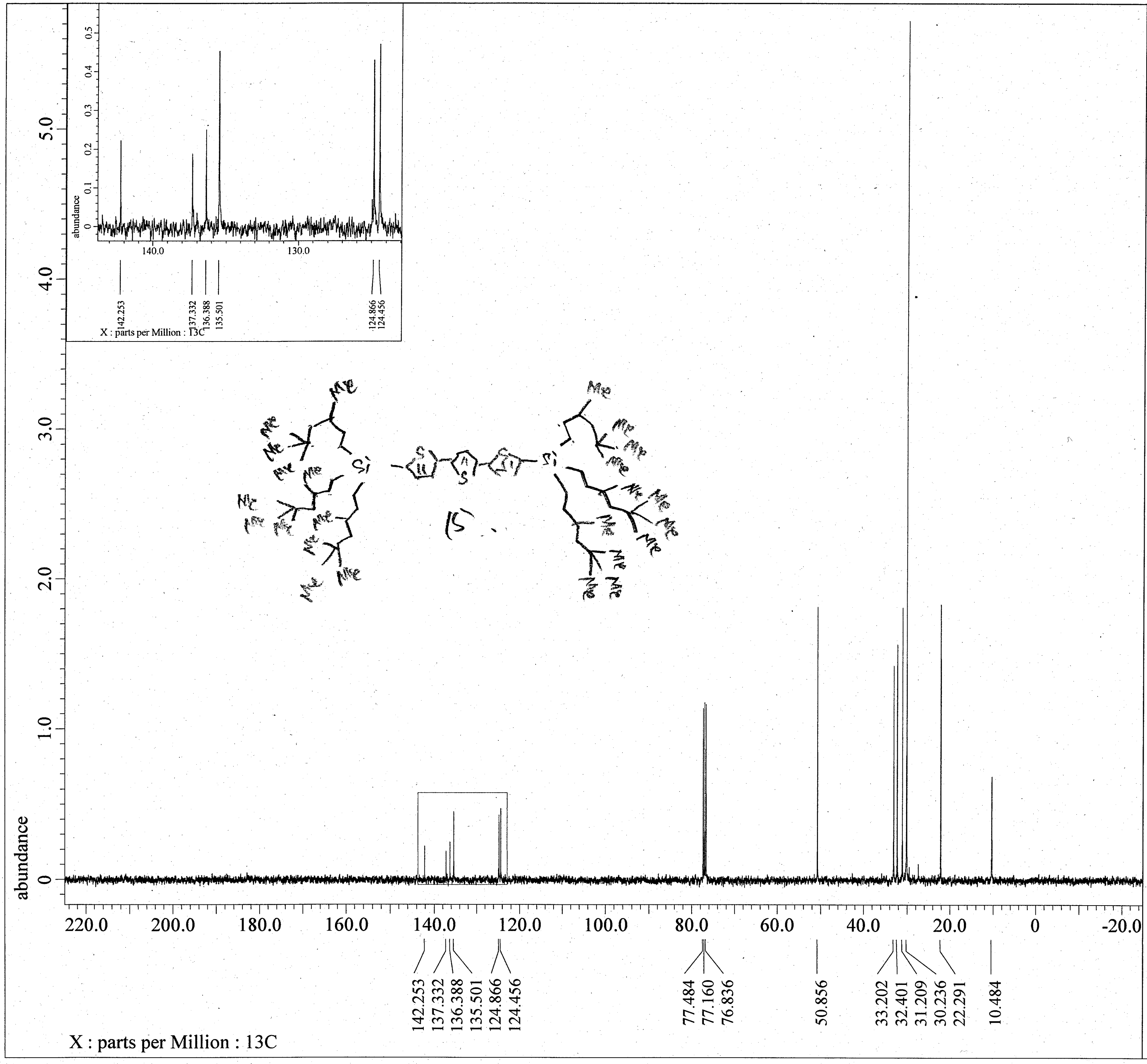


```
---- PROCESSING PARAMETERS ----
dc_balance( 0, FALSE )
sexp( 2.0[Hz], 0.0[s] )
trapezoid3( 0[%], 80[%], 100[%] )
zerofill( 1 )
fft( 1, TRUE, TRUE )
machinephase
ppm

Derived from: KND_214_13C-2.jdf

Filename            = KND_214_13C-3.jdf
Author              = element
Experiment          = single_pulse_dec
Sample_Id           = 1
Solvent             = CHLOROFORM-D
Actual_Start_Time   = 27-JAN-2022 20:15:51
Revision_Time       = 19-FEB-2023 17:25:43

Comment             = single pulse decoupled ga
Data_Format         = 1D COMPLEX
Dim_Size            = 26214
X_Domain            = 13C
Dim_Title           = 13C
Dim_Units           = [ppm]
Dimensions          = X
Site                = ECS 400
Spectrometer        = JNM-ECS400

Field_Strength      = 9.20197068[T] (390[MHz])
X_Acq_Duration      = 1.06430464[s]
X_Domain            = 13C
X_Freq              = 98.51479726[MHz]
X_Offset            = 100[ppm]
X_Points            = 32768
X_Prescans          = 4
X_Resolution        = 0.93958061[Hz]
X_Sweep             = 30.78817734[kHz]
Irr_Domain          = 1H
Irr_Freq            = 391.78655441[MHz]
Irr_Offset          = 5[ppm]
Clipped             = FALSE
Scans               = 26
Total_Scans         = 26

Relaxation_Delay    = 2[s]
Recvr_Gain          = 60
Temp_Get            = 19.7[dC]
X_90_Width          = 8.7[us]
X_Acq_Time          = 1.06430464[s]
X_Angle             = 30[deg]
X_Atn               = 4.9[dB]
X_Pulse             = 2.9[us]
Irr_Atn_Dec         = 22.45[dB]
Irr_Atn_Noe         = 22.45[dB]
Irr_Noise           = WALTZ
Decoupling          = TRUE
Initial_Wait        = 1[s]
Noe                 = TRUE
Noe_Time            = 2[s]
Repetition_Time     = 3.06430464[s]
```

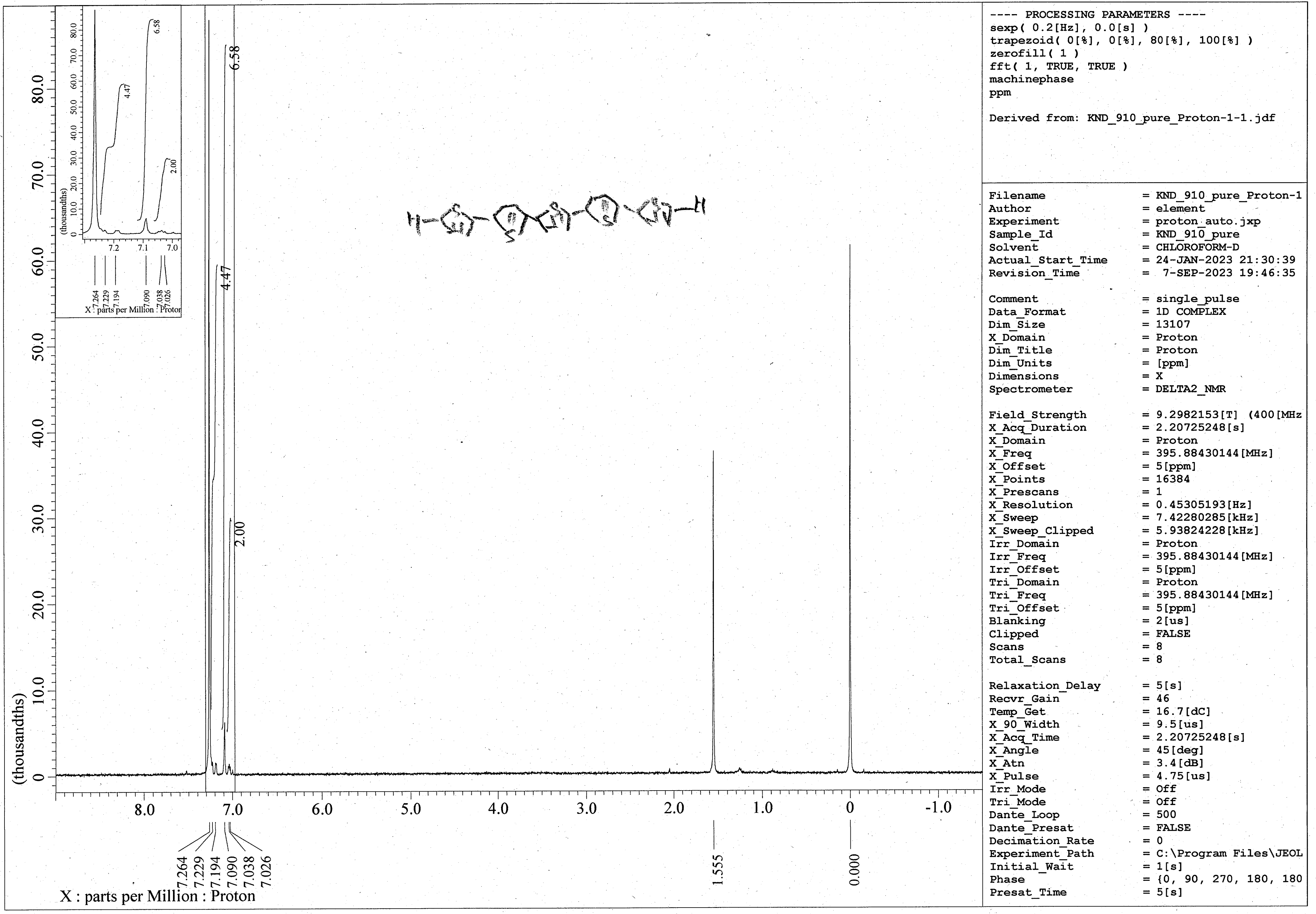
---- PROCESSING PARAMETERS ----
sexp(0.2[Hz], 0.0[s])
trapezoid(0[%], 0[%], 80[%], 100[%])
zerofill(1)
fft(1, TRUE, TRUE)
machinephase
ppm
Derived from: KND_910_pure_Proton-1-1.jdf
Filename = KND_910_pure_Proton-1
Author = element
Experiment = proton auto.jxp
Sample_Id = KND_910_pure
Solvent = CHLOROFORM-D
Actual_Start_Time = 24-JAN-2023 21:30:39
Revision_Time = 7-SEP-2023 19:46:35
Comment = single_pulse
Data_Format = 1D COMPLEX
Dim_Size = 13107
X_Domain = Proton
Dim_Title = Proton
Dim_Units = [ppm]
Dimensions = X
Spectrometer = DELTA2_NMR
Field_Strength = 9.2982153[T] (400[MHz
X_Acq_Duration = 2.20725248[s]
X_Domain = Proton
X_Freq = 395.88430144[MHz]
X_Offset = 5[ppm]
X_Points = 16384
X_Prescans = 1
X_Resolution = 0.45305193[Hz]
X_Sweep = 7.42280285[kHz]
X_Sweep_Clipped = 5.93824228[kHz]
Irr_Domain = Proton
Irr_Freq = 395.88430144[MHz]
Irr_Offset = 5[ppm]
Tri_Domain = Proton
Tri_Freq = 395.88430144[MHz]
Tri_Offset = 5[ppm]
Blanking = 2[us]
Clipped = FALSE
Scans = 8
Total_Scans = 8
Relaxation_Delay = 5[s]
Recvr_Gain = 46
Temp_Get = 16.7[dC]
X_90_Width = 9.5[us]
X_Acq_Time = 2.20725248[s]
X_Angle = 45[deg]
X_Atn = 3.4[dB]
X_Pulse = 4.75[us]
Irr_Mode = Off
Tri_Mode = Off
Dante_Loop = 500
Dante_Presat = FALSE
Decimation_Rate = 0
Experiment_Path = C:\Program Files\JEOL
Initial_Wait = 1[s]
Phase = {0, 90, 270, 180, 180
Presat_Time = 5[s]
(thousandths)
X : parts per Million : Proton
8.0 7.0 6.0 5.0 4.0 3.0 2.0 1.0 0 -1.0
7.264 7.229 7.194 7.090 7.038 7.026 1.555 0.000
6.58 4.47 2.00

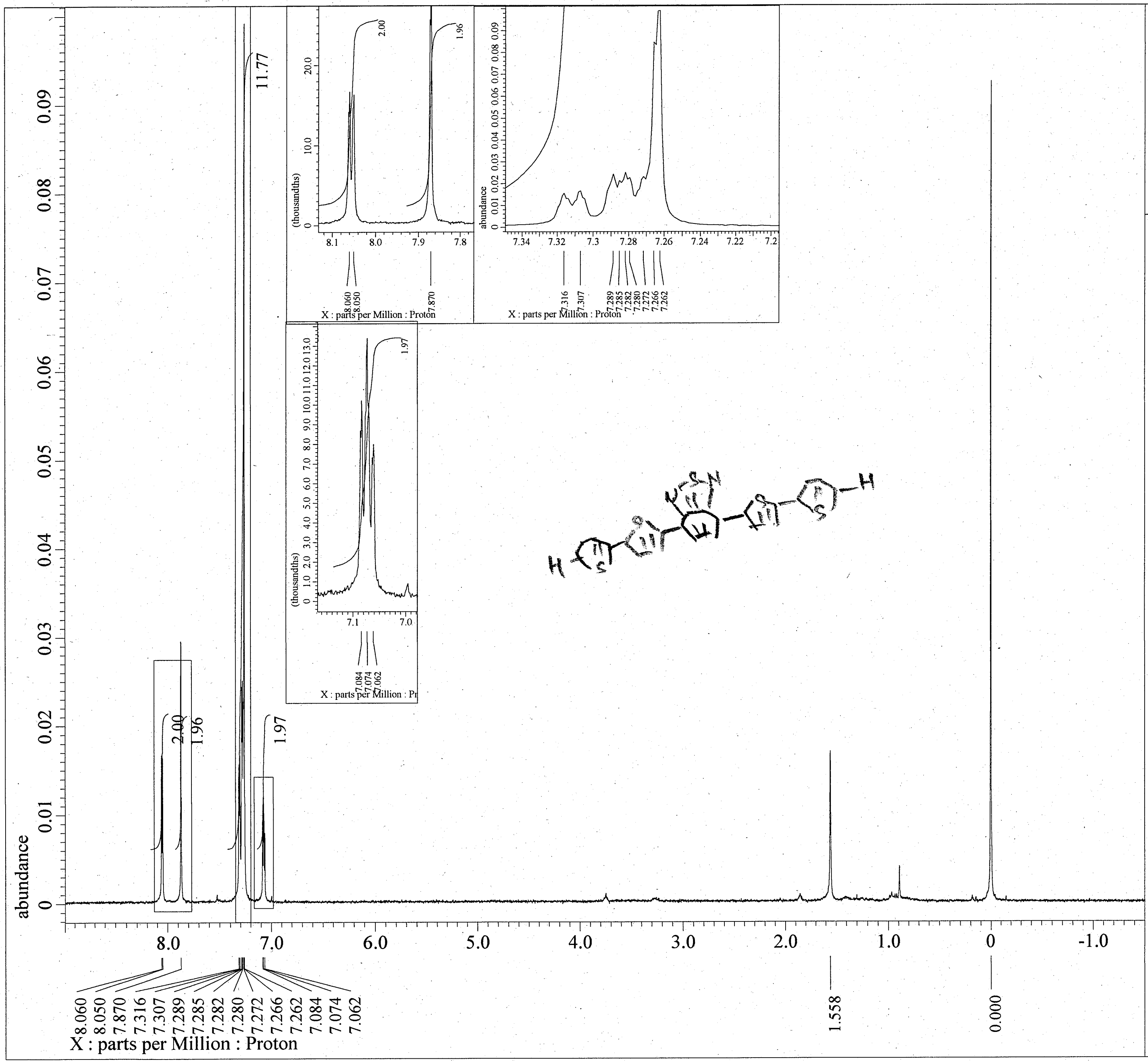


```
---- PROCESSING PARAMETERS ----
sexp( 0.2[Hz], 0.0[s] )
trapezoid( 0[%], 0[%], 80[%], 100[%] )
zerofill( 1 )
fft( 1, TRUE, TRUE )
machinephase
ppm

Derived from: KND_911_pure_Proton-1-1.jdf
```

```
Filename              = KND_911_pure_Proton-1
Author                = element
Experiment            = proton_auto.jxp
Sample_Id             = KND_911_pure
Solvent               = CHLOROFORM-D
Actual_Start_Time     = 19-JAN-2023 14:01:12
Revision_Time         =  7-SEP-2023 19:50:18

Comment               = single_pulse
Data_Format           = 1D COMPLEX
Dim_Size              = 13107
X_Domain              = Proton
Dim_Title             = Proton
Dim_Units             = [ppm]
Dimensions            = X
Spectrometer          = DELTA2_NMR

Field_Strength        = 9.2982153[T] (400[MHz
X_Acq_Duration        = 2.20725248[s]
X_Domain              = Proton
X_Freq                = 395.88430144[MHz]
X_Offset              = 5[ppm]
X_Points              = 16384
X_Prescans            = 1
X_Resolution          = 0.45305193[Hz]
X_Sweep               = 7.42280285[kHz]
X_Sweep_Clipped       = 5.93824228[kHz]
Irr_Domain            = Proton
Irr_Freq              = 395.88430144[MHz]
Irr_Offset            = 5[ppm]
Tri_Domain            = Proton
Tri_Freq              = 395.88430144[MHz]
Tri_Offset            = 5[ppm]
Blanking              = 2[us]
Clipped               = FALSE
Scans                 = 8
Total_Scans           = 8

Relaxation_Delay      = 5[s]
Recvr_Gain            = 46
Temp_Get              = 16.7[dC]
X_90_Width            = 9.5[us]
X_Acq_Time            = 2.20725248[s]
X_Angle               = 45[deg]
X_Atn                 = 3.4[dB]
X_Pulse               = 4.75[us]
Irr_Mode              = Off
Tri_Mode              = Off
Dante_Loop            = 500
Dante_Presat          = FALSE
Decimation_Rate       = 0
Experiment_Path       = C:\Program Files\JEOL
Initial_Wait          = 1[s]
Phase                 = {0, 90, 270, 180, 180
Presat_Time           = 5[s]
```

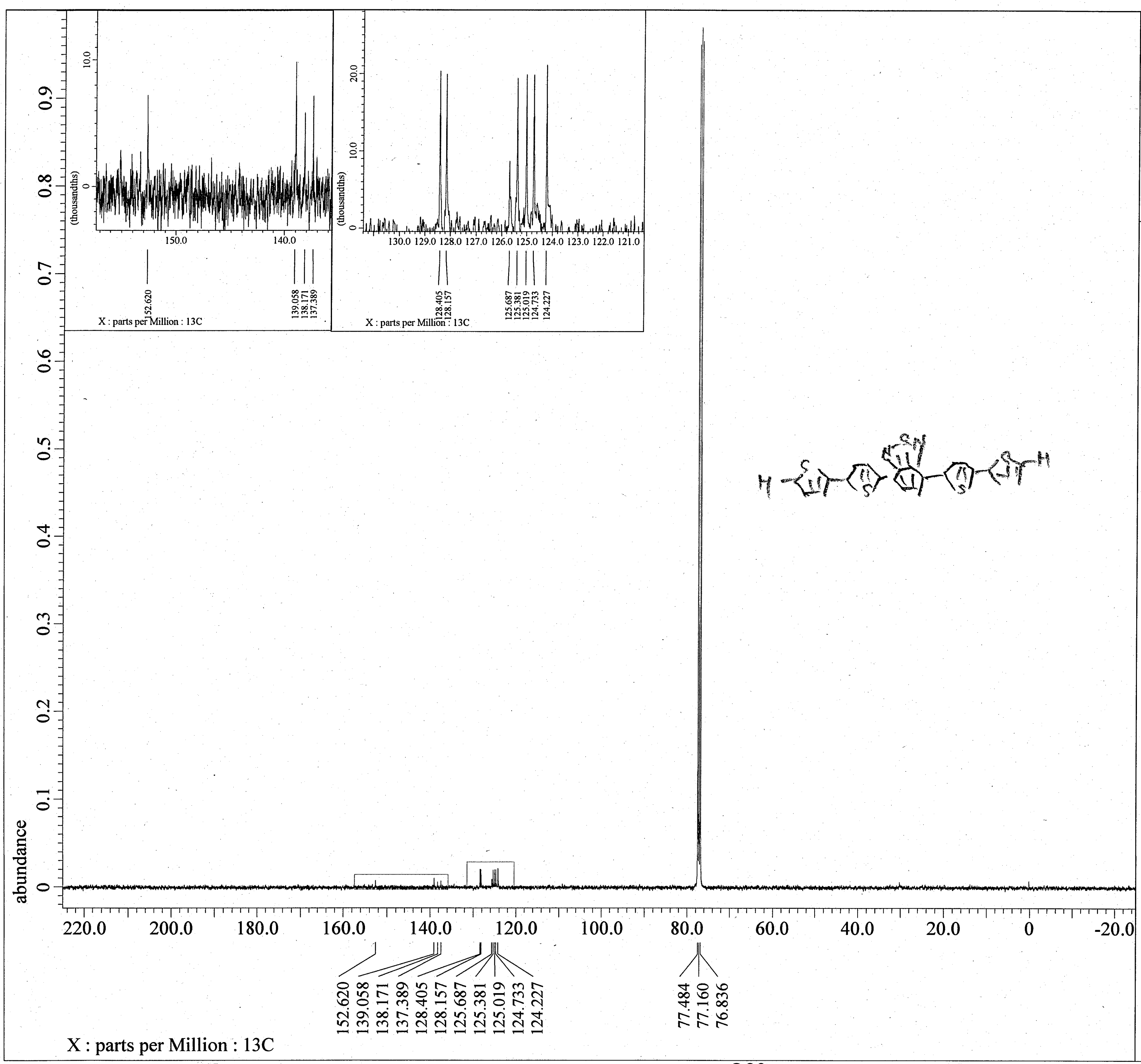


```
---- PROCESSING PARAMETERS ----
dc_balance( 0, FALSE )
sexp( 2.0[Hz], 0.0[s] )
trapezoid3( 0[%], 80[%], 100[%] )
zerofill( 1 )
fft( 1, TRUE, TRUE )
machinephase
ppm

Derived from: KND_911_13C2-1.jdf

Filename            = KND_911_13C2-2.jdf
Author              = element
Experiment          = single_pulse_dec
Sample_Id           = 1
Solvent             = CHLOROFORM-D
Actual_Start_Time   = 25-JAN-2023 08:17:13
Revision_Time       = 28-JAN-2023 20:38:44

Comment             = single pulse decoupled ga
Data_Format         = 1D COMPLEX
Dim_Size            = 26214
X_Domain            = 13C
Dim_Title           = 13C
Dim_Units           = [ppm]
Dimensions          = X
Site                = ECS 400
Spectrometer        = JNM-ECS400

Field_Strength      = 9.20197068[T] (390[MHz])
X_Acq_Duration      = 1.06430464[s]
X_Domain            = 13C
X_Freq              = 98.51479726[MHz]
X_Offset            = 100[ppm]
X_Points            = 32768
X_Prescans          = 4
X_Resolution        = 0.93958061[Hz]
X_Sweep             = 30.78817734[kHz]
Irr_Domain          = 1H
Irr_Freq            = 391.78655441[MHz]
Irr_Offset          = 5[ppm]
Clipped             = FALSE
Scans               = 3500
Total_Scans         = 3500

Relaxation_Delay    = 2[s]
Recvr_Gain          = 60
Temp_Get            = 18.6[dC]
X_90_Width          = 8.3[us]
X_Acq_Time          = 1.06430464[s]
X_Angle             = 30[deg]
X_Atn               = 4.9[dB]
X_Pulse             = 2.76666667[us]
Irr_Atn_Dec         = 22.45[dB]
Irr_Atn_Noe         = 22.45[dB]
Irr_Noise           = WALTZ
Decoupling          = TRUE
Initial_Wait        = 1[s]
Noe                 = TRUE
Noe_Time            = 2[s]
Repetition_Time     = 3.06430464[s]
```
